# Silicon-aryl cooperative activation of ammonia†

**DOI:** 10.1039/d4cc04617j

**Published:** 2024-10-01

**Authors:** Martin Ernst Doleschal, Arseni Kostenko, Jin Yu Liu, Shigeyoshi Inoue

**Affiliations:** a TUM School of Natural Sciences, Department of Chemistry, Catalysis Research Center and Wacker-Institute of Silicon Chemsitry, Technische Universität München (TUM) Lichtenbergstrasse 4 85748 Garching bei München Germany s.inoue@tum.de

## Abstract

Herein, we report the reactivity of N-heterocyclic carbene stabilized silylene-phosphinidene IDippPSi(TMS)_2_SiTol_3_ (IDipp = 1,3-bis(2,6-diisopropylphenyl)-imidazolin-2-ylidene) with ammonia, which results in an intermolecular 1,5-hydroamination and dearomatization of the NHC wingtip. DFT calculations reveal an unprecedented mechanism involving ammonia coordination to the silicon center, Meisenheimer-type complex formation, and a proton abstraction by the dearomatized aryl moiety.

## Abstract 2

The silicon-aryl cooperative N–H bond cleavage of ammonia was achieved at below room temperature, using an N-heterocyclic carbene-phosphinidine substituted acyclic silylene.

## Graphical abstract

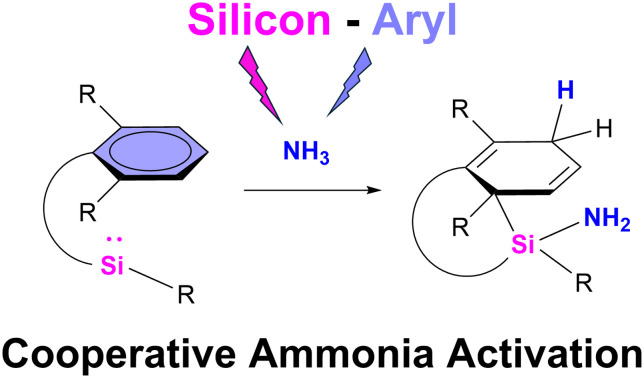

Ammonia has been playing an essential role in the chemical industry for decades, serving as a key reagent to produce various products. Among these, primary alkyl amines are particularly valued due to their wide range of applications.^1^ Their ideal synthetic route involves the hydroamination of unsaturated hydrocarbons with ammonia since this approach relies on inexpensive and abundant starting materials and ensures 100 per cent atom economy and minimal waste production. Consequently, enabling this reactivity is deemed the “holy grail” of catalysis.^2^ A particular challenge in this context is overcoming the N–H dissociation energy of 99.5 kcal mol^−1^.^3^ This can be achieved *via* activation at transition metal centres. A relatively common reactivity involves the deprotonation of coordinated ammonia (Scheme 1a), *e.g.* in metal-hydride species.^4–12^ Another approach of ammonia activation is oxidative addition to the metal (Scheme 1b).^13,14^ In recent years, multiple hydrogen atom transfer reactions from ammonia complexes have also been reported (Scheme 1c).^15–22^ Metal–ligand cooperativity, whereby a hydrogen atom is transferred to the ligand, has also proven effective for ammonia activation (Scheme 1d). This reactivity is most frequently enabled by unsaturated pyridine-based or carbene-based pincer ligands.^23–28^

**Scheme 1:**
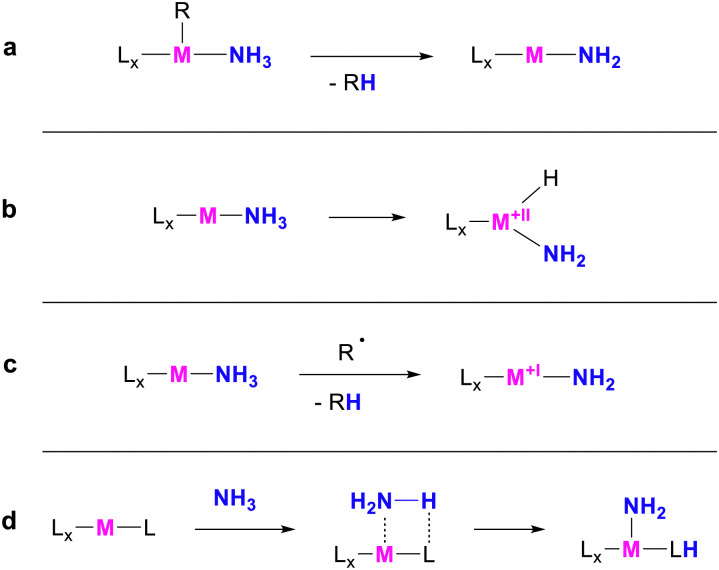
Common mechanisms for the activation of ammonia at transition metals. (a) Deprotonation (b) Oxidative Addition (c) Hydrogen Atom Transfer (d) Metal–Ligand Cooperativity.

Extensive efforts are focused on activating small molecules like ammonia using main group elements, aiming for transition-metal-free catalysis.^29–32^ Notably, the group of *Breher* recently reported the reversible activation of ammonia with an aluminium-carbon-based ambiphile and demonstrated several catalytic ammonia transfer reactions.^33^ Carbenes (R_2_C:) and their heavier analogues are promising candidates for main-group catalysis as they exhibit such an ambiphilic character due to the presence of a free electron pair and an empty p-orbital.^34^ At tetrylene centres, ammonia activation typically results in 1,1-oxidative addition, which has been observed both irreversibly and reversibly with carbenes.^35,36^ Similar findings have been reported for silylenes^37–44^ and germylenes.^45–47^ In the case of a diboryl-stannylene, isolation of its ammonia complex and its oxidative addition product was achieved.^48^ A few examples of cooperative 1,4-addition of ammonia have been reported with silylenes and germylenes; however, they are all based on 1,3-ketimine ligands.^49–51^ Computational and experimental studies revealed that these and other ammonia or hydrazine activation pathways at tetrylene centres proceed *via* intermolecular proton shuffling – mechanisms that involve a second equivalent of NH_3_.^52,53^

Herein, we report a unique N–H activation of a single ammonia molecule *via* silylene-aryl cooperativity. In previous work, we isolated NHC-stabilized silylene-phosphinidene 1, which predominantly displays the reactivity of acyclic silylenes.^54^ Exposure of 1 to 1 bar of ammonia rapidly furnished compound 2 in 74% isolated yield (Scheme 2). Its central silicon atom displays a ^29^Si NMR signal at 3.2 ppm (^1^*J*_Si-P_ = 91.8 Hz), which is in agreement with other aminosilanes.^42,43^ The ^31^P NMR signal at -133.1 ppm (^1^*J*_Si-P_ = 91.8 Hz) is observed in higher fields as opposed to its precursor (269.4 ppm, ^1^*J*_Si–P_ = 187.5 Hz) and falls within the range of common – especially hydrogen substituted – NHCPs.^55^ SiH-HMBC measurements revealed the amine signal (1.05 ppm) overlaps with those of the isopropyl groups. 2 demonstrates good thermal stability in solution up to 110 °C. The structure of 2 was confirmed by X-ray crystallography (Scheme 2). It displays a Si–P bond length of 2.228(1) Å, which is elongated compared to 1 (2.1311(7) Å), and within the typical range of Si–P single bonds.^56^ With 1.760(2) Å the C–P bond is slightly shorter in comparison to 1 (1.844(2) Å). C–C bond lengths in the dearomatized wingtip are consistent with the 1,4-dearomatization, revealing shortened bonds (1.323(3) Å, 1.346(3) Å) between the sp^2^-carbon centres. The Si–N bond (1.734(2) Å) lies within the upper range of bond lengths observed in comparable literature examples.^44,48^ Nitrogen congeners of 1 featuring N-heterocyclic imines (NHIs) or cyclic alkyl amino carbene (CAAC) imines have been reported; however, they exhibit no comparable reactivity towards ammonia.^57–59^

**Scheme 2:**
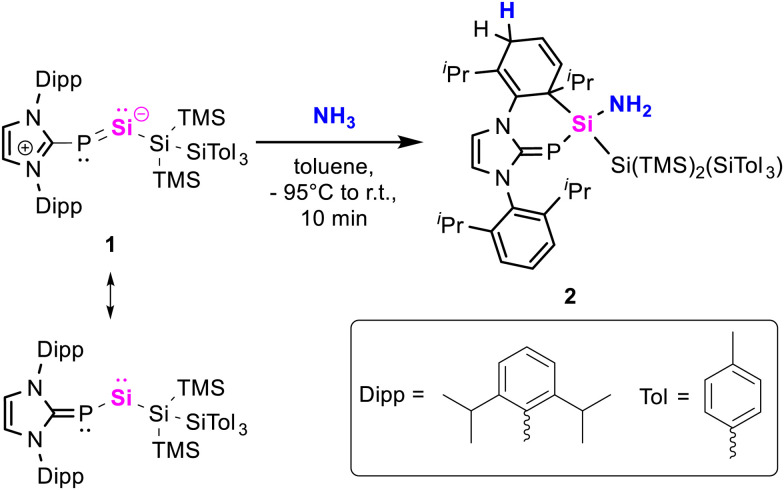
Silicon-aryl-cooperative hydroamination of silylene-phosphinidene 1.

The proposed mechanism for the reaction of 1 in the presence of ammonia to form 2 is presented in Fig. 2. Initially, ammonia coordinates to the silylene 1*via*TS(A-B) (at Δ*G* = 11.0 kcal mol^−1^) to give the silylene ammonia adduct intermediate A at −2.2 kcal mol^−1^. The ammonia-coordinated silylene A undergoes a nucleophilic addition to the Dipp substituent of the NHC *via*TS(A-B), dearomatizing the aryl and forming the corresponding Meisenheimer-type complex B. Similar intramolecular dearomatization processes of aryl substituents are well-known occurrences in silylene chemistry;^57,60^ however, in this case, the dearomatization step takes place in the presence of a coordinating ammonia molecule. In intermediate B, one of the protons of the coordinating ammonia is found in close proximity (2.159 Å) to C5 (Fig. 1), placing it in a favourable orientation for a formal 1,6-H shift from the nitrogen to the carbon atom. At this stage, the proton is abstracted from the nitrogen atom *via* the rate-determining transition state TS(B-2) (at 17.4 kcal mol^−1^) to form the final product 2. The reaction is exergonic by 11.9 kcal mol^−1^. Overall, the low energy barriers agree with the experimental observations of the reaction occurring almost immediately, even at low temperatures. The barrier for the reverse reaction TS(2-B) with Δ*G*^‡^ of 29.3 kcal mol^−1^ makes the whole process essentially irreversible at the reaction conditions. We also considered a scenario in which the dearomatization takes place prior to ammonia coordination, which would ultimately lead to formation of the corresponding silepin E (Fig. S7, ESI†). However, the transition state for this step TS(1-C) at 20.3 kcal mol^−1^ is 4.3 kcal mol^−1^ higher than the transition state for dearomatization of A, (*i.e.*TS(A-B), at 15.9). Furthermore, the rate-determining step for the formation of the silepin TS(D-E) (Fig. S7, ESI†) is 6.9 kcal mol^−1^ higher than the rate-determining step TS(B-2) for the formation of 2 (Fig. 2).

**Fig. 1:**
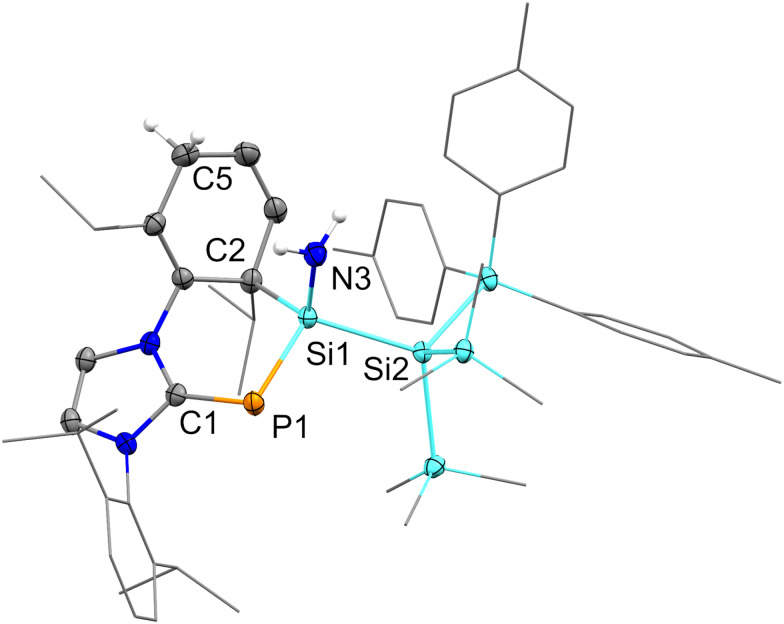
Molecular structure of 2. Thermal ellipsoids are set at 50% probability. Most hydrogen atoms are omitted for clarity. Dipp- Methyl- and Tolyl- substituents are depicted as wireframes. Selected bond distances (Å) and angles (°): C1–P1: 1.760(2), P1–Si1: 2.228(1), Si1–N3: 1.734(2), Si1–C2: 1.970(2), Si1–Si2: 2.3962(8), C1–P1–Si1: 97.84(8), C2–Si1–P1: 109.14(7), C2–Si1–N3: 107.6(1).

**Fig. 2:**
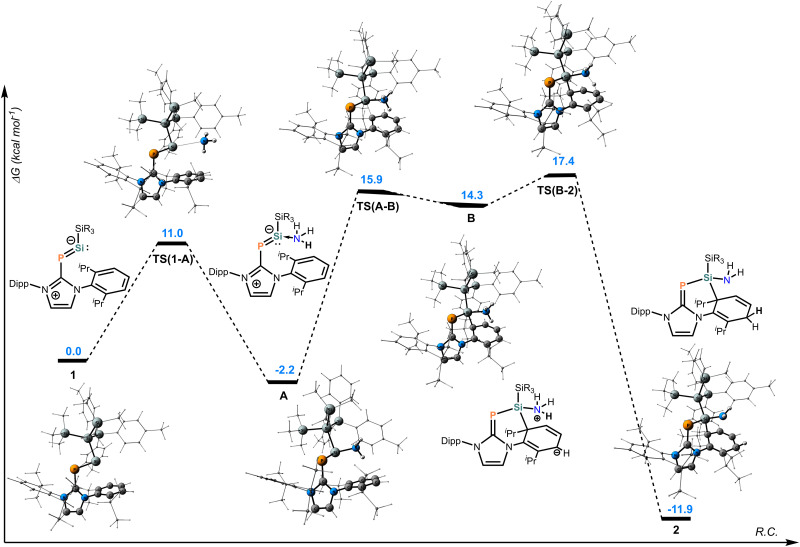
Free energy reaction coordinate diagram of the proposed mechanism for the 1 + NH_3_ → 2 transformation at the PW6B95-D4/def2-QZVPP(SMD = Benzene)//r^2^SCAN-3c level of theory.

An alternative reaction outcome, in which 1 undergoes the 1,1-oxidative addition at the silicon centre by insertion into the N–H bond of ammonia, which is a known process for silylenes,^37–44^ was also considered (Fig. S8, ESI†). Although this process is predicted to be highly exergonic (by 26.2 kcal mol^−1^ relative to the starting compounds), its barrier with Δ*G* = 35.1 kcal mol^−1^ is much higher than TS(B-2) and is unachievable under the reaction conditions. For completeness, we also calculated the ammonia activation by **1***via* proton shuffling for both 1,1-addition and the silicon-aryl cooperative pathways (Fig. S9 and S10, ESI†). Both of these pathways are kinetically less preferable than the 1,5-hydroamination presented in Fig. 2 by 4.6 and 10.4 kcal mol^−1^, respectively.

In summary, an unprecedented activation mechanism of ammonia by an NHC-stabilized silylene-phosphinidene has been shown. After the initial formation of a silylene-ammonia adduct, a Meisenheimer-type complex is formed, and ultimately, ammonia gets deprotonated by the aryl group, resulting in intramolecular hydroamination and dearomatization. This reactivity differs from literature-known activations of ammonia at tetrylene centres and represents a novel example of silicon-aryl cooperativity. In the resulting complex 2, the distance between the abstracted proton and the nitrogen atom of the NH_2_ moiety is 3.138 Å, according to the calculations. Such an arrangement can allow facile ammonia transfer to appropriate substrates, such as unsaturated organic compounds, *via* the aryl rearomatization. We are currently investigating the substituent effects on this ammonia activation and the potential utilization of complexes of type 2 as ammonia transfer reagents.

We thank the Wacker Chemie AG for their scientific and financial support. M. E. D. acknowledges Tobias Weng for LIFDI-MS measurements. The authors gratefully acknowledge the computational and data resources the Leibniz Supercomputing Centre provided and are grateful to the European Research Council (ALLOWE101001591) for financial support.

## Data availability

The data supporting this article have been included in the ESI.† Crystallographic data for 2 has been deposited at the Cambridge Crystallographic Data Centre (CCDC) under deposition number 2377544 and can be obtained from https://www.ccdc.cam.ac.uk/structures/.

## Conflicts of interest

There are no conflicts to declare.

## Supplementary Material

CC-060-D4CC04617J-s001

CC-060-D4CC04617J-s002
