# Supplementary material for: Silicon-aryl cooperative activation of ammonia
Source: Chem Commun (Camb). 2024 Oct 1;60(89):13020–3. doi: 10.1039/d4cc04617j (PMC11492217; doi:10.1039/d4cc04617j)
Supplement: CC-060-D4CC04617J-s001 [file CC-060-D4CC04617J-s001.pdf]

Supplementary Information (SI) for ChemComm.
This journal is © The Royal Society of Chemistry 2024

# Supporting Information

## Silicon-Aryl Cooperative Activation of Ammonia

*Martin Ernst Doleschal, Arseni Kostenko, Jin Yu Liu and Shigeyoshi Inoue**

*s.inoue@tum.de

# Table of Content

Experimental ........ 3
Spectra ........ 5
Computational Details ........ 8
Crystalographic Details ........ 42
References ........ 45

# Experimental

### General procedures

All experiments were performed under a dry argon (≥99.996%) atmosphere using standard *Schlenk* techniques or in a glovebox (MBraun GmbH). Non-deuterated solvents used were distilled over sodium/benzophenone and stored over molecular sieve prior to use. Ammonia (99.98%) was purchased from Westfalen AG. Liquid Injection Field Desorption Ionization Mass Spectrometry (LIFDI-MS) was measured directly from an inert atmosphere glovebox with a Thermo Fisher Scientific Exactive Plus Orbitrap equipped with an ion source from Linden CMS.[1] NMR spectra were recorded on *Bruker* AV-400 or AV-500C spectrometers at ambient temperature (300 K). $^{1}$H, $^{13}$C, and $^{29}$Si chemical shifts $\delta$ are reported in parts per million (ppm) relative to tetramethylsilane. $\delta$($^{1}$H) and $\delta$($^{13}$C) were referenced internally to the relevant residual solvent resonances. $\delta$($^{29}$Si) was referenced to the signal of tetramethylsilane (δ = 0 ppm) as external standard. For reported signals, the following abbreviations are used: s = singlet, d = doublet, hept = heptet, m = multiplet/signal overlap, br = broad signal, dd = doublet of doublets, dt = doublet of triplets. Melting Points (m.p.) were determined in sealed glass capillaries under an inert gas atmosphere using a Büchi B-540 melting point apparatus. Compound **1** was prepared according to literature procedures.[2]

**3-(2,6-diisopropylphenyl)-5-(1,1,1,3,3,3-hexamethyl-2-(tri-p-tolylsilyl)trisilan-2-yl)-5a,9-diisopropyl-3,5,5a,8-tetrahydrobenzo[e]imidazo[1,2-a][1,3,4]azaphosphasilin-5-amine** (**2**)

In a *J-Young* PTFE tube, IDippPSi(TMS)$_2$(SiTol$_3$) (**1**, 50 mg, 54.1 µmol) was dissolved in toluene and briefly immersed in liquid nitrogen until the solvent was frozen solid. The remaining argon atmosphere was removed *in vacuo* and ammonia (1 bar) was filled into the tube. While the solution was warmed to room temperature, its color changed from dark brown to yellow. The complete formation of **2** was confirmed *via* $^{31}$P NMR. Crystals of compound **2** could be obtained from a saturated pentane solution (37.5 mg, 74%).

$^1$H NMR (500 MHz, $C_6D_6$) δ 7.89 (d, *J* = 8.0 Hz, 6H), 7.19 – 7.13 (m, 2H), 7.10 (d, *J* = 7.5 Hz, 6H), 7.03 (dd, *J* = 5.7, 3.5 Hz, 1H), 6.20 (dd, *J* = 2.2, 1.2 Hz, 1H), 6.09 (dd, *J* = 2.2, 1.1 Hz, 1H), 5.50 (dt, *J* = 10.0, 3.4 Hz, 1H), 5.40 (dt, *J* = 9.9, 1.8 Hz, 1H), 3.06 (hept, *J* = 6.8 Hz, 1H), 2.82 (hept, *J* = 6.9 Hz, 1H), 2.75 – 2.65 (m, 2H), 2.11 (s, 9H), 2.12 – 1.99 (m, 2H), 1.51 (d, *J* = 6.8 Hz, 3H), 1.43 (d, *J* = 6.8 Hz, 3H), 1.11 (d, *J* = 6.9 Hz, 3H), 1.06 (d, *J* = 7.0 Hz, 3H), 1.05 (br, 2H), 1.03 (d, *J* = 7.0 Hz, 3H), 0.94 (dd, *J* = 9.3, 6.8 Hz, 6H), 0.82 (d, *J* = 6.9 Hz, 3H), 0.49 (d, *J* = 5.5 Hz, 18H).

$^{13}$C NMR (126 MHz, $C_6D_6$) δ 173.19 (d, J = 103.5 Hz), 147.58, 146.68, 138.80, 137.64, 136.07, 134.95, 133.90, 132.48, 130.32, 129.76, 128.82, 128.35, 124.75, 124.22, 120.64, 119.94 (d, J = 4.9 Hz), 118.87, 48.39 (d, J = 2.5 Hz), 31.37, 29.17, 28.81 (d, J = 7.0 Hz), 26.60, 24.78 (d, J = 6.5 Hz), 24.18, 24.04 (d, J = 3.5 Hz), 22.54, 21.53, 21.47, 21.44, 20.22, 4.87 (d, J = 5.5 Hz), 4.65 (d, J = 3.9 Hz).

$^{29}$Si NMR (99 MHz, $C_6D_6$) δ 3.23 (d, *J* = 91.8 Hz, P-*Si*), -9.30 (d, *J* = 67.3 Hz, *TMS*), -10.35 (d, *J* = 9.6 Hz, *Si*Tol$_3$), -121.07 (d, *J* = 27.4 Hz, *Si*(TMS)$_2$(SiTol$_3$)).

$^{31}$P NMR (162 MHz, $C_6D_6$) δ -133.09.

LIFDI-MS: calculated: 939.4780; found: 939.4768.

m.p.: 214.4 °C

# Spectra

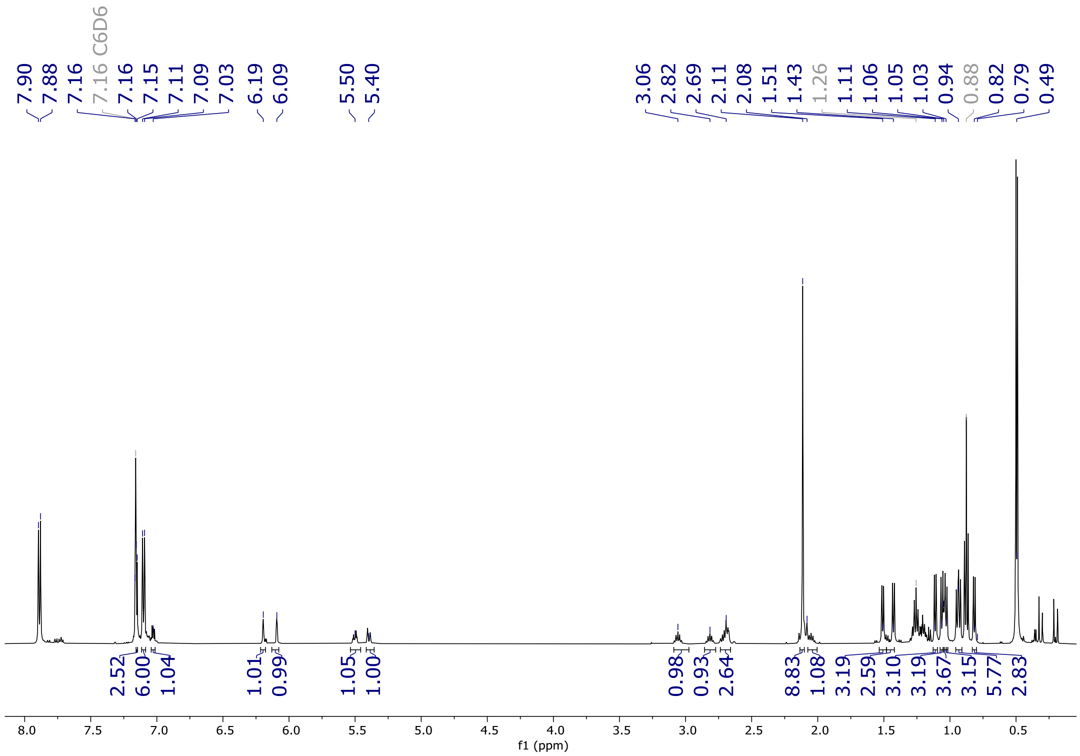


**Supplementary Figure 1:** $^{1}$H NMR of **2**. Signals marked in grey correspond to co-crystallized pentane.

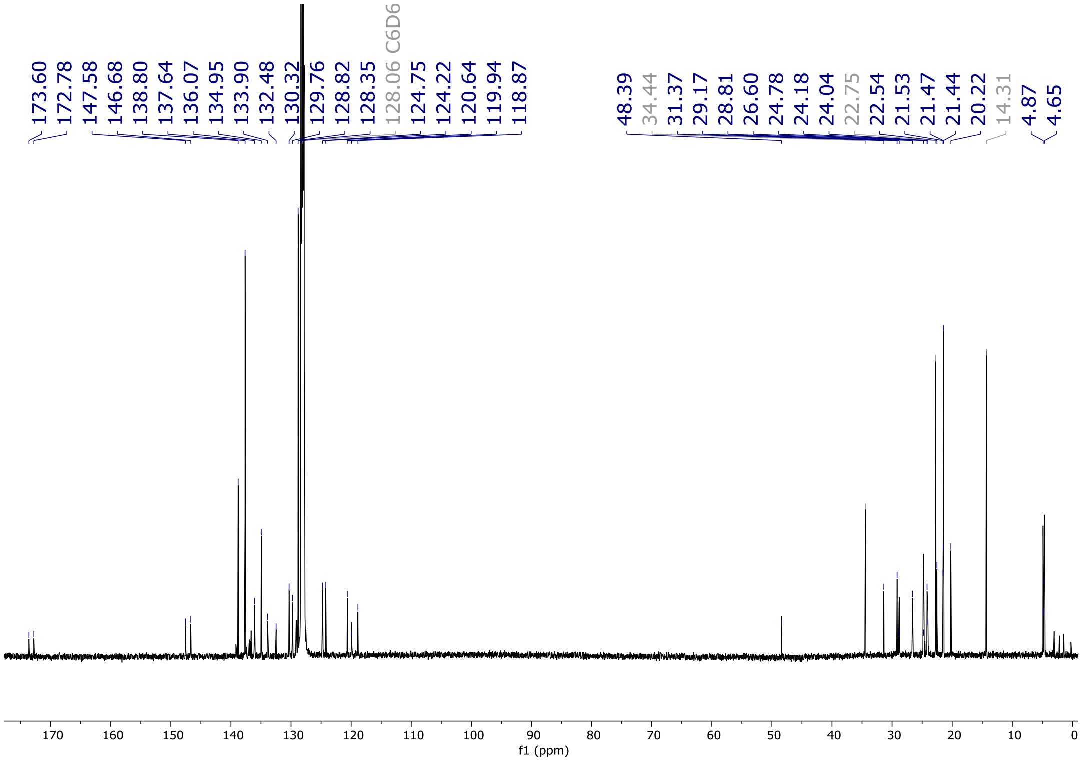


**Supplementary Figure 2:** $^{13}$C NMR of **2**. Signals marked in grey correspond to co-crystallized pentane.

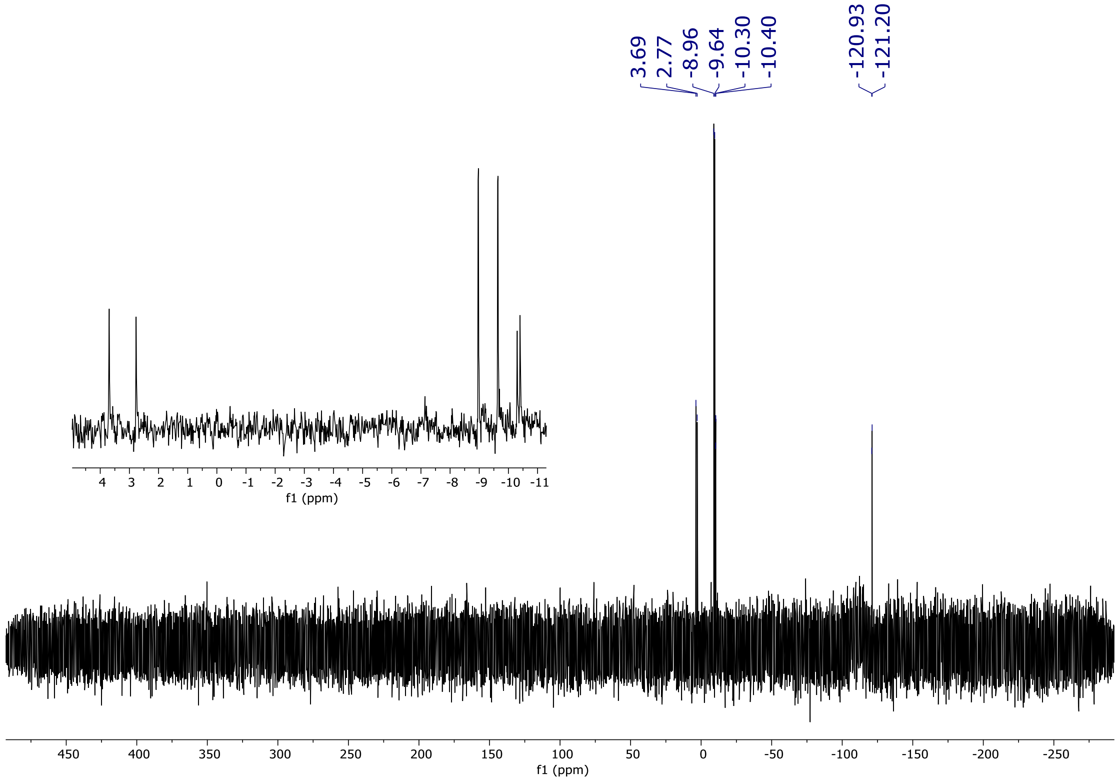


**Supplementary Figure 3:** $^{29}$Si NMR of **2**.

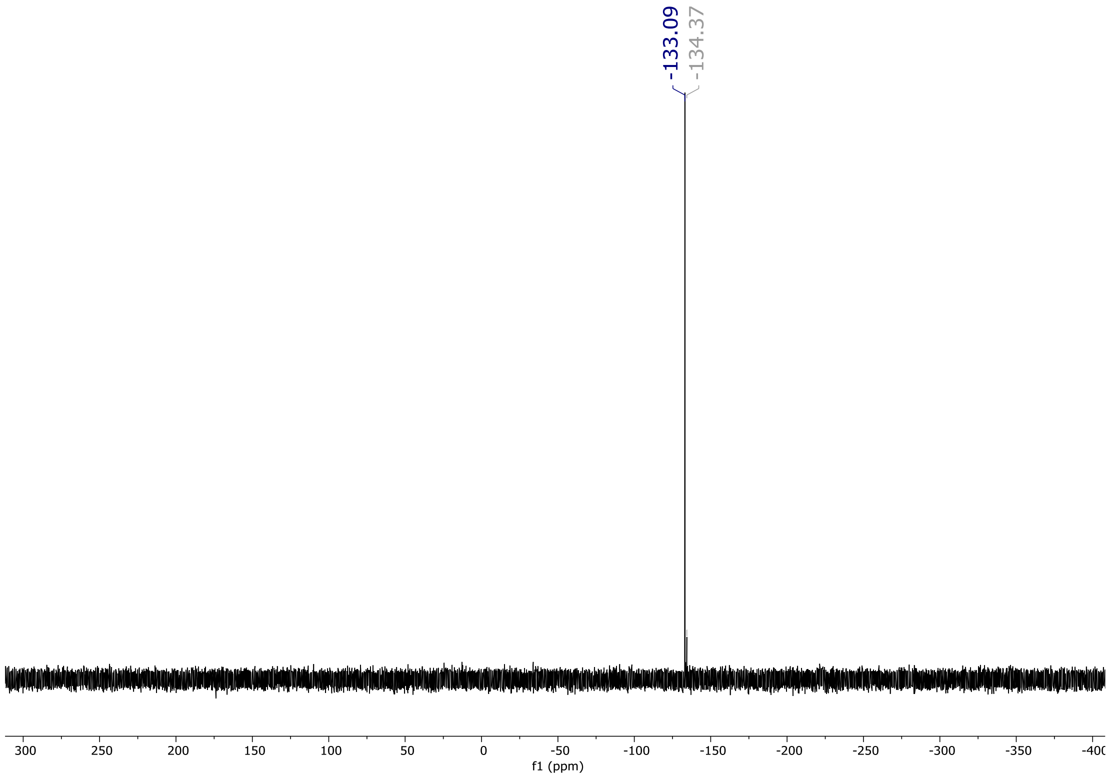


**Supplementary Figure 4:** $^{31}$P NMR of **2**. The signal in grey is IDippPH, a ubiquitous impurity when working with Dipp-NHCPs.

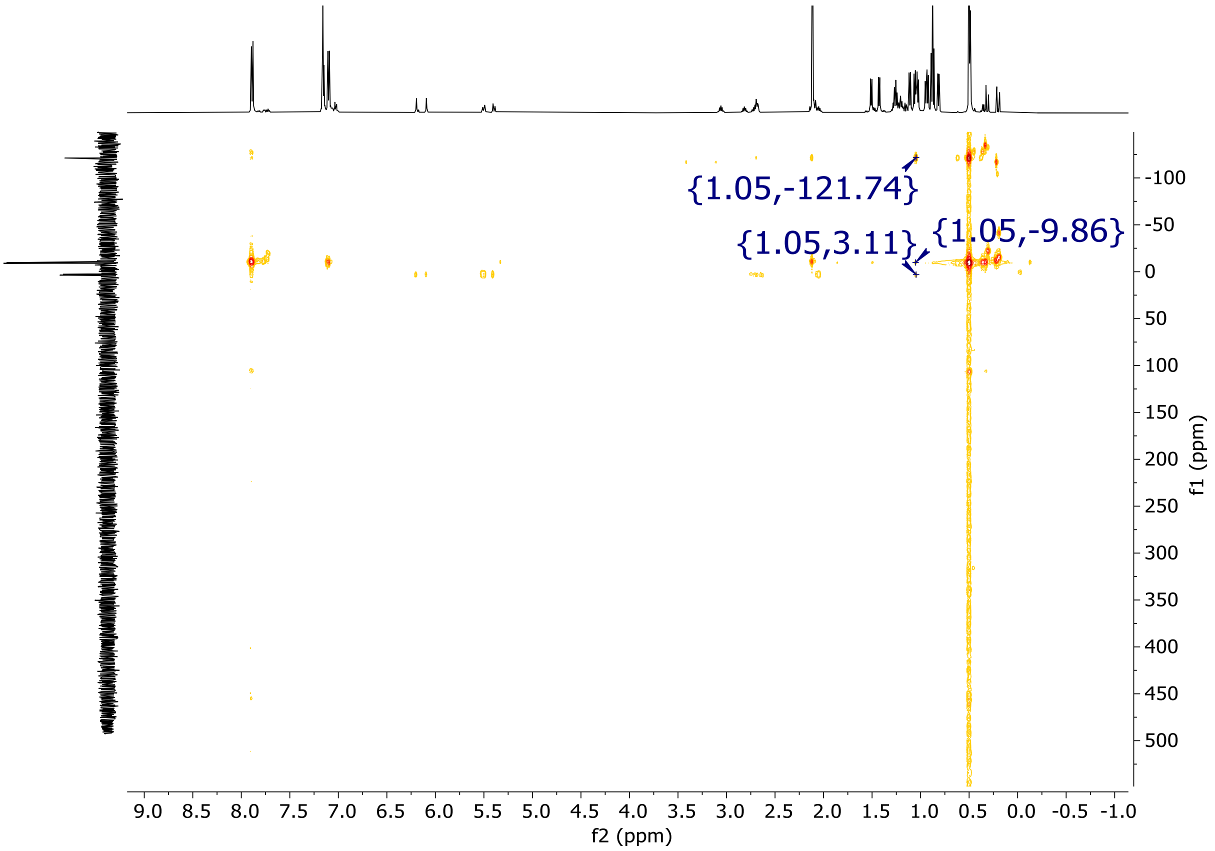


**Supplementary Figure 5**: SiH HMBC spectrum of **2**.

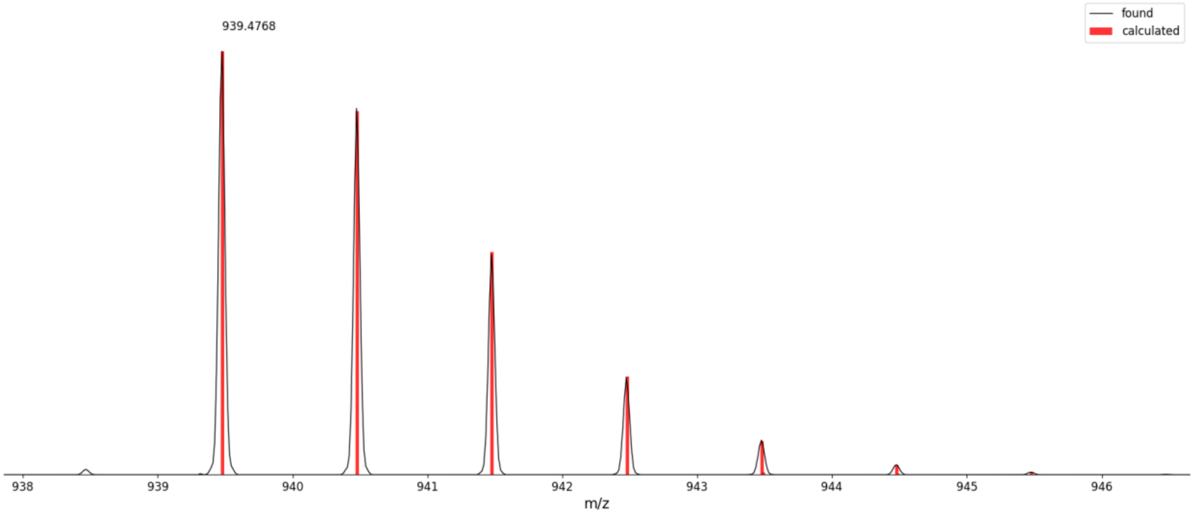


**Supplementary Figure 6:** LIFDI-MS spectrum of **2**.

# Computational Details

Calculations were carried out using ORCA 5.0.4 software.[3]

Geometry optimizations were carried using the $r^2$SCAN-3c composite method, utilizing the regularized and restored SCAN functional,[4, 5] geometrical counterpoise correction gCP,[6] the atom-pairwise dispersion correction based on tight binding partial charges (D4,[7-9] the def2-mTZVPP basis set and def2-mTZVPP/J auxiliary basis set.[10]

The optimized geometries were verified as minima or transition states by analytical frequency calculations. The transition states were additionally verified by IRC calculations. Single point calculations of the optimized geometries were carried out at the $r^2$SCAN-3c level in benzene using the SMD solvation module[11] to obtain electrostatic contribution and the cavity term to account for the solvent effects.

To get more accurate electronic energies for the mechanistic investigations, single point calculations of the $r^2$SCAN-3c optimized geometries were carried out using the PW6B95[12] functional, with D4 dispersion correction, the def2-QZVPP[13] basis set and def2/J[14] and def2-QZVPP/C[15] auxiliary basis sets. The method is denoted as PW6B95-D4(SMD=Benzene)/def2-QZVPP//$r^2$SCAN-3c. The summary of the thermochemistry results is presented in Supplementary Table 1.

**Supplementary Table 1**: Calculated energies (Eh). $E_{PW6B95}$ - electronic energy at the PW6B95-D4/def2-QZVPP//r$^2$SCAN-3c level; G-$E_{el}$ - Gibbs energy minus the electronic energy at the r$^2$SCAN-3c// r$^2$SCAN-3c level; $G_{cds}$ (cavity term) and $G_{enp}$ (electrostatic contribution) at r$^2$SCAN-3c SMD=Benzene)// r$^2$SCAN-3c level; $G_{conc}$ - concentration-induced free-energy shift ($G_{conc}$ = RTln(24.5); G – free energy at the PW6B95-D4(SMD=Benzene)/def2-QZVPP//r$^2$SCAN-3c level, G = $E_{PW6B95}$ + [G-$E_{el}$] + $G_{cds}$ + $G_{enp}$ + $G_{conc}$. Thermochemistry at 298.15 K.

| Compound | ID | $E_{PW6B95}$ | G-$E_{el}$ | $G_{cds}$ | $G_{enp}$ | $G_{conc}$ | G |
|---|---|---|---|---|---|---|---|
| $NH_3$ | 3755810 | -56.65245 | 0.01638 | -0.00500 | 0.00237 | 0.00302 | -56.63568 |
| **1** | 3519997 | -4007.10251 | 1.05124 | -0.02633 | -0.02620 | 0.00302 | -4006.10077 |
| **TS(1-A)** | 3519955 | -4063.75419 | 1.08593 | -0.02749 | -0.02623 | 0.00302 | -4062.71897 |
| **A** | 3520171 | -4063.77777 | 1.08904 | -0.02758 | -0.02674 | 0.00302 | -4062.74003 |
| **TS(A-B)** | 3519949 | -4063.75442 | 1.09113 | -0.02534 | -0.02551 | 0.00302 | -4062.71112 |
| **B** | 3520170 | -4063.75635 | 1.09098 | -0.02581 | -0.02555 | 0.00302 | -4062.71371 |
| **TS(B-2)** | 3519725 | -4063.74961 | 1.08816 | -0.02520 | -0.02506 | 0.00302 | -4062.70870 |
| **2** | 3518893 | -4063.80112 | 1.09138 | -0.02377 | -0.02489 | 0.00302 | -4062.75537 |
| **TS(1-C)** | 3520303 | -4007.07561 | 1.05237 | -0.02201 | -0.02625 | 0.00302 | -4006.06848 |
| **C** | 3520751 | -4007.07735 | 1.05216 | -0.02179 | -0.02606 | 0.00302 | -4006.07003 |
| **TS(C-D)** | 3979989 | -4007.07945 | 1.05337 | -0.02116 | -0.02544 | 0.00302 | -4006.06966 |
| **D** | 3919971 | -4007.09070 | 1.05402 | -0.02103 | -0.02492 | 0.00302 | -4006.07960 |
| **TS(D-E)** | 3919973 | -4007.06803 | 1.05392 | -0.02167 | -0.02582 | 0.00302 | -4006.05857 |
| **E** | 3922447 | -4007.09744 | 1.05451 | -0.02161 | -0.02590 | 0.00302 | -4006.08742 |
| **TS(A-F)** | 3919972 | -4063.71407 | 1.08275 | -0.02544 | -0.02670 | 0.00302 | -4062.68044 |
| **F** | 3975127 | -4063.81571 | 1.08678 | -0.02589 | -0.02642 | 0.00302 | -4062.77822 |
| **G** | 4152498 | -4120.43877 | 1.12460 | -0.02931 | -0.02495 | 0.00302 | -4119.36541 |
| **TS(G-H)** | 4144987 | -4120.41135 | 1.12410 | -0.02756 | -0.02531 | 0.00302 | -4119.33710 |
| **H** | 4147236 | -4120.47569 | 1.11953 | -0.02669 | -0.02406 | 0.00302 | -4119.40389 |
| **I** | 4144972 | -4120.41603 | 1.12303 | -0.02775 | -0.02392 | 0.00302 | -4119.34164 |
| **TS(I-J)** | 4140701 | -4120.40055 | 1.12399 | -0.03002 | -0.02421 | 0.00302 | -4119.32778 |
| **J** | 4143801 | -4120.45885 | 1.12545 | -0.02685 | -0.02338 | 0.00302 | -4119.38061 |

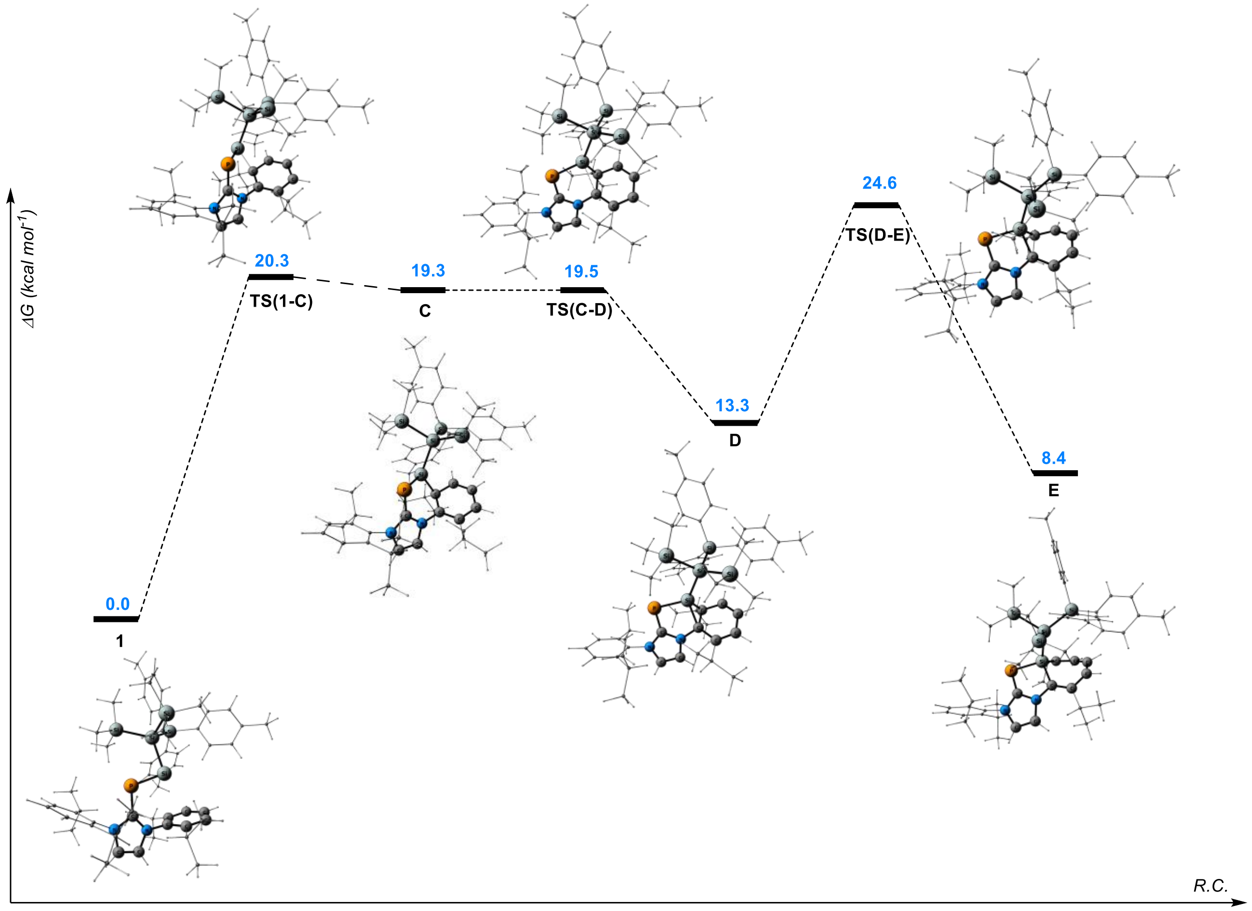


**Supplementary Figure 7:** Calculated free energy reaction coordinate diagram of the proposed mechanism for the conversion of **1** to the silepin **E** at the PW6B95-D4/def2-QZVPP(SMD = Benzene)//r$^2$SCAN-3c level of theory.

Supplementary Figure **7** describes the intermolecular insertion of the silylene into the C-C bond of the aryl substituent of the NHCP moiety. The first step is the formation of the Meisenheimer-type complex **C**, which rearranges to the formal product of [1+2] cycloaddition of the Si across the aromatic C-C bond **D**. The final step is the ring expansion to yield the final silepin product **E**, via the rate determining transition state **TS(D-E)** at 24.6 kcal mol$^{-1}$. **TS(D-E)** is by 7.2 kcal mol$^{-1}$ higher than the rate determining transition state **TS(B-2)** that leads to the formation of **2**. In any case, the whole process is endergonic by 8.4 kcal mol$^{-1}$ and thus the **E** would not be observed even with more favorable kinetics. In addition, the transition state **TS(1-C)**, which leads to the Meisenheimer-type complex **C**, is also higher than **TS(B-2)**. Thus, we rule out **C** as an intermediate in the formation of **2**.

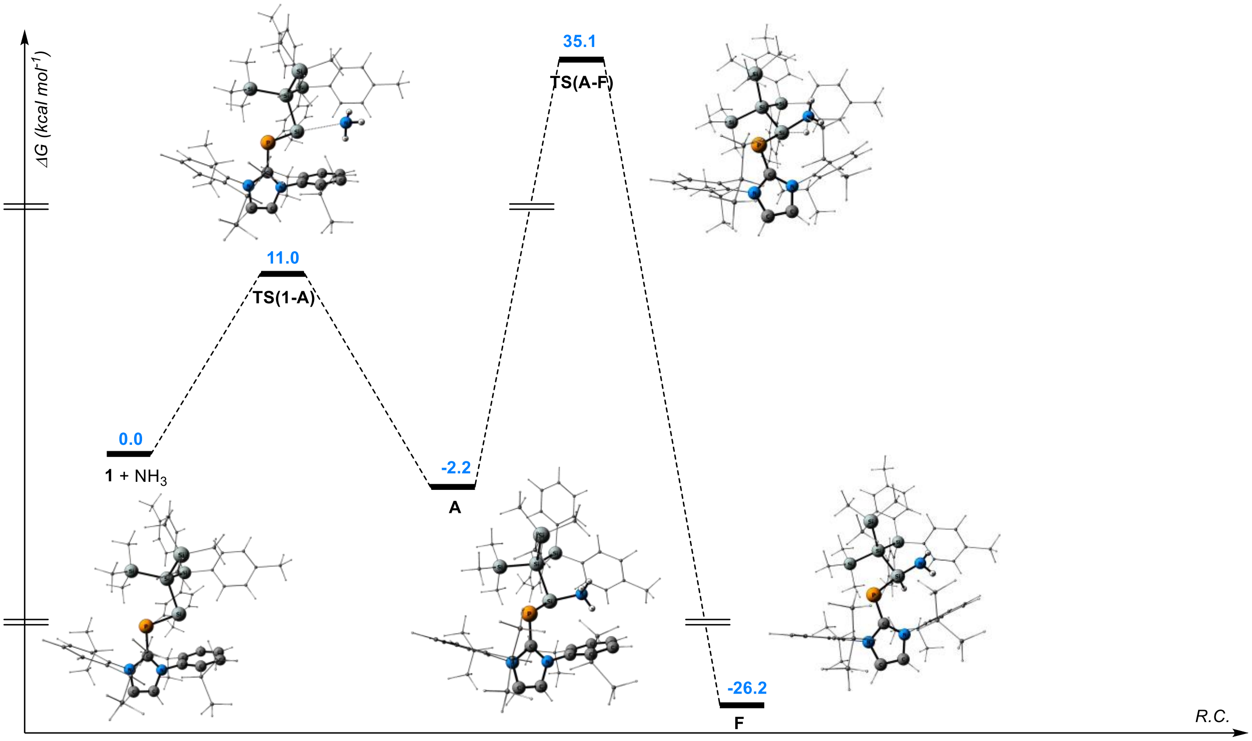


**Supplementary Figure 8:** Calculated free energy reaction coordinate diagram for 1,1-additon of ammonia at the Si center of **1** at the PW6B95-D4/def2-QZVPP(SMD = Benzene)//r$^2$SCAN-3c level of theory.

Supplementary Figure **8** describes the oxidative addition of the silylene by insertion into the N-H bond of ammonia. Upon initial coordination of an ammonia molecule to the silylene center via **TS(1-A)** the resulting complex **A** undergoes the 1,1-addition to the form the product **F** in a very exergonic step. However, the barrier for this reaction **TS(A-F)** of 37.3 kcal mol$^{-1}$ is infeasible.

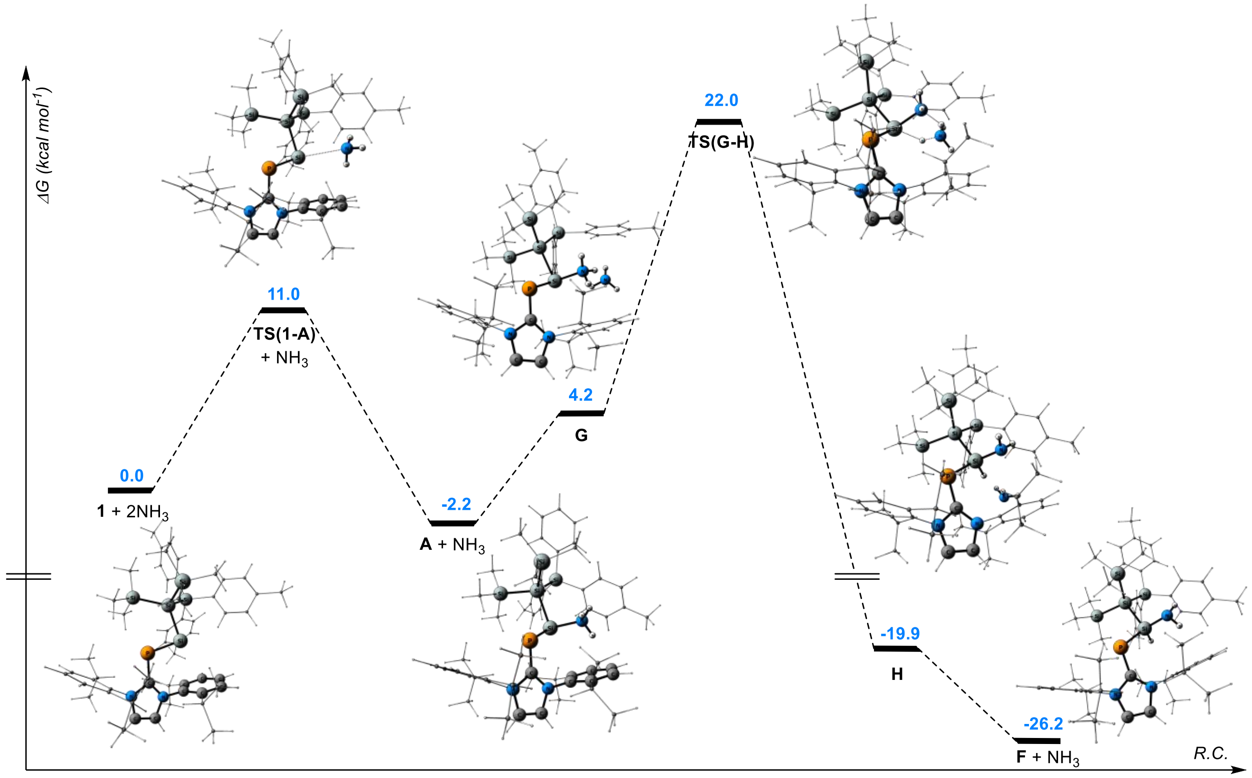


**Supplementary Figure 9:** Calculated free energy reaction coordinate diagram for 1,1-additon of ammonia at the Si center of **1** via proton shuffling mechanism at the PW6B95-D4/def2-QZVPP(SMD = Benzene)//r$^2$SCAN-3c level of theory.

Supplementary Figure **9** describes the oxidative addition of the Si by two ammonia molecules. Upon formation of complex **A**, a second molecule of $NH_3$ can abstract a proton for the ammonia molecule that is coordinated to the silicon center, while simultaneously transferring a proton the silicon center. The rate determining transition state **TS(G-H)** at 22.0 kcal mol$^{-1}$ is by 4.6 kcal mol$^{-1}$ lower in energy than **TS(B-2)**. Therefore, the activation of ammonia via the mechanism presented in Fig. 2 of the main text, in which the single ammonia molecule is activated across the Si and the C centers is much more favorable.

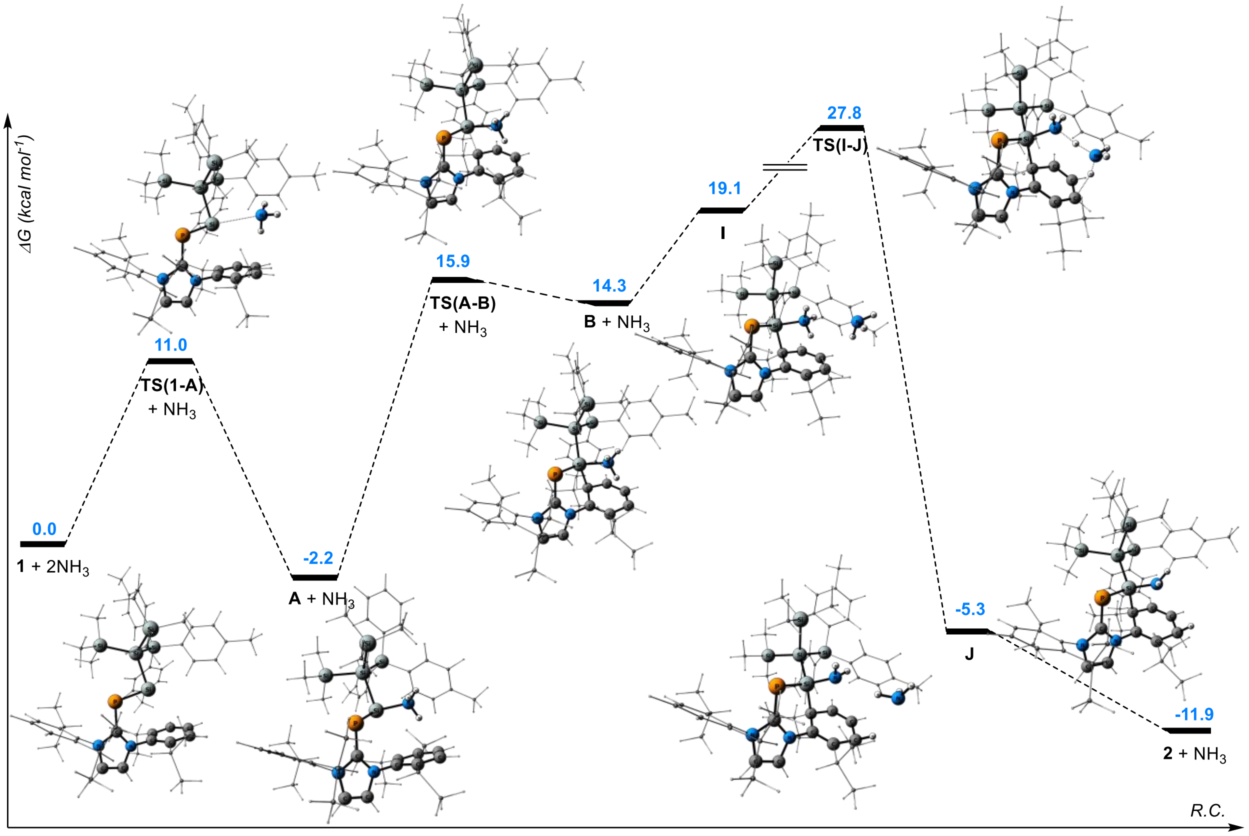


**Supplementary Figure 10:** Calculated free energy reaction coordinate diagram for cooperative silylene-aryl activation of ammonia via proton shuffling mechanism at the PW6B95-D4/def2-QZVPP(SMD = Benzene)//r$^2$SCAN-3c level of theory.

Supplementary Figure **10** describes the ammonia activation across the Si center of the silylene and the C center of the aryl via a proton shuffling mechanism. Upon formation of complex **B**, a second molecule of $NH_3$ comes in. Via **TS(I-J)** a proton from the first ammonia molecule that is coordinated to the Si center is transferred to second ammonia molecule, while a proton from the second ammonia molecule is transferred to the C center. The rate determining transition state **TS(I-J)** at 27.8 kcal mol$^{-1}$ is by 10.4 kcal mol$^{-1}$ lower in energy than **TS(B-2)**. Therefore, the mechanism presented in Fig. 2 of the main text, in which the single ammonia molecule is activated across the Si and the C centers is much more favorable.

## Cartesian coordinates and energies of the optimized geometries at the r$^2$SCAN-3c level

Calculated energies and coordinates of **1**

Electronic energy ... -4000.71363631 Eh
Total Enthalpy ... -3999.48791788 Eh
Final Gibbs free energy ... -3999.66239289 Eh

CARTESIAN COORDINATES (ANGSTROEM)

| | | | |
|---|---|---|---|
| P | -0.697162 | 0.845006 | 0.261773 |
| Si | 0.221508 | 1.043881 | -1.689356 |
| Si | 1.383547 | -1.040523 | -1.677125 |
| Si | 1.164175 | -2.597273 | 0.095317 |
| Si | 0.953097 | -2.112637 | -3.745026 |
| Si | 3.603009 | -0.199500 | -1.786728 |
| N | -2.796463 | 2.760232 | 0.131386 |
| N | -0.905300 | 3.687177 | 0.640264 |
| C | -3.057957 | 0.872668 | -3.642785 |
| H | -3.958890 | 0.510970 | -4.150503 |
| H | -2.541894 | 0.017096 | -3.194222 |
| H | -2.398815 | 1.302055 | -4.404005 |
| C | -3.377281 | 1.931188 | -2.583569 |
| H | -2.413282 | 2.323477 | -2.236452 |
| C | -4.107830 | 1.356968 | -1.387362 |
| C | -3.815318 | 1.764635 | -0.078899 |
| C | -1.471650 | 2.488759 | 0.309794 |
| C | 1.599561 | -4.333456 | -0.514929 |
| C | 0.613279 | -5.306269 | -0.720104 |
| H | -0.421285 | -5.081523 | -0.468520 |
| C | 0.929709 | -6.561993 | -1.231684 |
| H | 0.139925 | -7.295791 | -1.379853 |
| C | 2.245761 | -6.898273 | -1.550566 |
| C | 2.587803 | -8.241855 | -2.134553 |
| H | 2.677894 | -8.179992 | -3.226225 |
| H | 1.815811 | -8.983037 | -1.908615 |
| H | 3.543576 | -8.611556 | -1.749860 |
| C | -5.129532 | 0.421050 | -1.549398 |
| H | -5.388243 | 0.079859 | -2.546796 |
| C | -5.819787 | -0.081710 | -0.453700 |
| H | -6.614153 | -0.807976 | -0.603811 |
| C | -5.495118 | 0.331223 | 0.830149 |
| H | -6.030843 | -0.080853 | 1.680344 |
| C | -4.480121 | 1.263766 | 1.048270 |
| C | -4.105990 | 1.659717 | 2.464049 |
| H | -3.381151 | 2.480328 | 2.416204 |
| C | -5.315965 | 2.174776 | 3.253705 |
| H | -5.809013 | 3.003257 | 2.734031 |
| H | -4.997321 | 2.527904 | 4.240309 |
| H | -6.059123 | 1.385877 | 3.410472 |
| C | -3.428403 | 0.484500 | 3.183666 |
| H | -4.120363 | -0.358769 | 3.285675 |
| H | -3.111965 | 0.787466 | 4.188514 |
| H | -2.552844 | 0.131122 | 2.629056 |
| C | -4.177854 | 3.093446 | -3.190266 |
| H | -3.633505 | 3.531701 | -4.033821 |
| H | -4.358230 | 3.883362 | -2.453161 |
| H | -5.151342 | 2.744421 | -3.553946 |
| C | -3.050674 | 4.105187 | 0.347704 |
| H | -4.043776 | 4.516834 | 0.258581 |
| C | -1.869089 | 4.683672 | 0.670612 |
| H | -1.624935 | 5.702931 | 0.923232 |
| C | 0.462899 | 3.863812 | 1.052721 |
| C | 1.374427 | 4.434833 | 0.153945 |
| C | 2.683876 | 4.615003 | 0.602774 |
| H | 3.422301 | 5.047060 | -0.065113 |
| C | 3.062169 | 4.229448 | 1.880667 |
| H | 4.090051 | 4.367251 | 2.203803 |
| C | 2.136109 | 3.662342 | 2.745440 |
| H | 2.444316 | 3.371762 | 3.745304 |
| C | 0.811388 | 3.468973 | 2.353749 |
| C | -0.203351 | 2.915535 | 3.337856 |
| H | -1.091685 | 2.602770 | 2.779980 |
| C | 0.301233 | 1.675148 | 4.080266 |
| H | 0.596485 | 0.890820 | 3.377907 |
| H | -0.495650 | 1.276052 | 4.716464 |
| H | 1.153891 | 1.902573 | 4.729407 |
| C | -0.632509 | 4.015888 | 4.320973 |
| H | -1.035224 | 4.887743 | 3.793919 |
| H | 0.219212 | 4.351534 | 4.923571 |
| H | -1.404165 | 3.638800 | 5.001646 |
| C | 0.972440 | 4.872536 | -1.242228 |
| H | 0.034794 | 4.363979 | -1.498783 |
| C | 2.001147 | 4.472707 | -2.305416 |
| H | 1.605349 | 4.695089 | -3.301790 |
| H | 2.213120 | 3.400668 | -2.262485 |
| H | 2.940803 | 5.024158 | -2.192848 |
| C | 0.734790 | 6.390910 | -1.278726 |
| H | 1.660391 | 6.928441 | -1.042574 |
| H | -0.023849 | 6.705056 | -0.555005 |
| H | 0.405154 | 6.702094 | -2.275966 |
| C | 0.708572 | -0.819893 | -5.105265 |
| H | 0.479582 | -1.316025 | -6.057015 |
| H | 1.596079 | -0.197124 | -5.251886 |
| H | -0.124537 | -0.151940 | -4.859775 |
| C | 2.354285 | -3.276869 | -4.248514 |
| H | 2.135141 | -3.732204 | -5.222695 |
| H | 2.474118 | -4.081672 | -3.515101 |
| H | 3.311833 | -2.751460 | -4.331092 |
| C | -0.636565 | -3.136948 | -3.676493 |
| H | -1.515463 | -2.507578 | -3.496604 |
| H | -0.594141 | -3.900423 | -2.892636 |
| H | -0.785099 | -3.648377 | -4.636135 |
| C | 3.888305 | 1.199199 | -0.542510 |
| H | 4.761017 | 1.792613 | -0.844649 |
| H | 4.078636 | 0.801484 | 0.459139 |
| H | 3.029895 | 1.875631 | -0.468737 |
| C | 3.891082 | 0.524189 | -3.513271 |
| H | 4.876314 | 1.005550 | -3.556162 |
| H | 3.134552 | 1.278181 | -3.758653 |

| | | | |
|---|---|---|---|
| H | 3.860403 | -0.247190 | -4.289873 |
| C | 4.928666 | -1.509002 | -1.465581 |
| H | 4.846390 | -2.355413 | -2.155562 |
| H | 4.854084 | -1.888523 | -0.439903 |
| H | 5.925677 | -1.066650 | -1.587713 |
| C | -0.602938 | -2.677756 | 0.753117 |
| C | -1.709571 | -2.487030 | -0.082404 |
| H | -1.555259 | -2.201348 | -1.120214 |
| C | -3.008368 | -2.630329 | 0.391646 |
| H | -3.847985 | -2.461127 | -0.279401 |
| C | -3.249340 | -2.967830 | 1.724771 |
| C | -4.652758 | -3.175747 | 2.225763 |
| H | -5.369079 | -2.582078 | 1.649607 |
| H | -4.746708 | -2.906807 | 3.282870 |
| H | -4.947517 | -4.228455 | 2.130838 |
| C | -2.151531 | -3.134634 | 2.572154 |
| H | -2.316745 | -3.379876 | 3.619765 |
| C | -0.852464 | -2.993663 | 2.094808 |
| H | -0.017207 | -3.125919 | 2.780055 |
| C | 2.323366 | -2.155551 | 1.518061 |
| C | 2.443154 | -0.823396 | 1.938030 |
| H | 1.879339 | -0.044265 | 1.425673 |
| C | 3.270410 | -0.471660 | 2.998006 |
| H | 3.343284 | 0.573468 | 3.295073 |
| C | 4.014306 | -1.436859 | 3.681157 |
| C | 3.892758 | -2.767395 | 3.275464 |
| H | 4.454545 | -3.539060 | 3.798260 |
| C | 3.063930 | -3.120210 | 2.214199 |
| H | 2.992028 | -4.166207 | 1.923903 |
| C | 4.940524 | -1.048112 | 4.801383 |
| H | 5.082177 | -1.872635 | 5.506392 |
| H | 4.556287 | -0.184768 | 5.353523 |
| H | 5.929361 | -0.774472 | 4.412549 |
| C | 2.921170 | -4.683124 | -0.830919 |
| H | 3.719718 | -3.960155 | -0.682105 |
| C | 3.239373 | -5.939059 | -1.331668 |
| H | 4.275437 | -6.179319 | -1.563233 |

Calculated energies and coordinates of **TS[1-A]**

Electronic energy ... -4057.25049648 Eh
Total Enthalpy ... -4055.98548895 Eh
Final Gibbs free energy ... -4056.16457146 Eh

CARTESIAN COORDINATES (ANGSTROEM)

| | | | |
|---|---|---|---|
| Si | -0.206789 | 9.257034 | 9.531505 |
| Si | -1.417474 | 10.302546 | 11.270695 |
| Si | -0.385266 | 9.248172 | 13.175517 |
| P | -2.151866 | 9.579011 | 14.441484 |
| Si | -3.737436 | 9.927626 | 10.911178 |
| Si | -1.063664 | 12.649281 | 11.266174 |
| N | -2.915405 | 8.505622 | 16.879019 |
| N | -0.915283 | 7.789666 | 16.441301 |
| N | 2.030988 | 11.293416 | 13.324057 |
| C | -3.077104 | 10.410957 | 4.128630 |
| H | -4.097578 | 10.753037 | 4.340327 |
| H | -3.149317 | 9.522485 | 3.495135 |
| H | -2.581649 | 11.201973 | 3.556213 |
| C | -2.338294 | 10.116192 | 5.405490 |
| C | -2.143774 | 8.806799 | 5.844810 |
| H | -2.513188 | 7.980872 | 5.240334 |
| C | -1.487761 | 8.540798 | 7.044610 |
| H | -1.356245 | 7.508030 | 7.360152 |
| C | -1.005497 | 9.576054 | 7.852910 |
| C | -1.906494 | 8.602327 | 15.947349 |
| C | -4.133899 | 9.272733 | 16.861371 |
| C | -4.159727 | 10.479442 | 17.570770 |
| C | -2.935405 | 11.045117 | 18.265398 |
| H | -2.163304 | 10.268321 | 18.291704 |
| C | -3.225535 | 11.441983 | 19.718013 |
| H | -3.919419 | 12.287234 | 19.775700 |
| H | -2.298278 | 11.745873 | 20.215422 |
| H | -3.661124 | 10.609506 | 20.280678 |
| C | -6.472407 | 10.722928 | 16.899144 |
| H | -7.395050 | 11.296175 | 16.909107 |
| C | -6.413104 | 9.524646 | 16.196988 |
| H | -7.288993 | 9.180044 | 15.657988 |
| C | -5.240992 | 8.772202 | 16.158397 |
| C | -5.358253 | 11.196482 | 17.573830 |
| H | -5.412390 | 12.142987 | 18.103424 |
| C | -2.368823 | 12.229218 | 17.467376 |
| H | -2.107985 | 11.926973 | 16.447444 |
| H | -1.470301 | 12.621051 | 17.957207 |
| H | -3.101867 | 13.041397 | 17.404578 |
| C | -2.540195 | 7.684563 | 17.927893 |
| H | -3.192119 | 7.506272 | 18.768577 |
| C | -1.291153 | 7.241149 | 17.656842 |
| H | -0.629894 | 6.595110 | 18.212358 |
| C | -0.101599 | 7.387865 | 9.816439 |
| C | -0.882145 | 6.707941 | 10.755499 |
| H | -1.597282 | 7.259198 | 11.362241 |
| C | -0.765087 | 5.332338 | 10.939389 |
| H | -1.394930 | 4.834250 | 11.673546 |
| C | 0.147777 | 4.584574 | 10.196440 |
| C | 0.940772 | 5.256711 | 9.260789 |
| H | 1.667187 | 4.697001 | 8.674477 |
| C | 0.816727 | 6.627937 | 9.075161 |
| H | 1.458660 | 7.123614 | 8.348760 |
| C | 0.266177 | 3.095538 | 10.378905 |
| H | -0.256908 | 2.560134 | 9.577019 |
| H | -0.171017 | 2.775183 | 11.329338 |
| H | 1.312307 | 2.773494 | 10.354872 |
| C | -1.851387 | 11.156623 | 6.203279 |
| H | -1.992879 | 12.187364 | 5.883453 |
| C | -1.207021 | 10.890071 | 7.403199 |
| H | -0.868017 | 11.725297 | 8.013935 |
| C | 1.597052 | 9.828555 | 9.470837 |
| C | 2.123177 | 10.629002 | 8.450974 |
| H | 1.486991 | 10.936504 | 7.623916 |
| C | 3.455975 | 11.030121 | 8.459005 |
| H | 3.834595 | 11.650443 | 7.648993 |
| C | 4.318595 | 10.642441 | 9.485330 |
| C | 5.750398 | 11.103902 | 9.513778 |
| H | 5.851408 | 12.025683 | 10.100366 |
| H | 6.121398 | 11.314780 | 8.506472 |

| | | | |
|---|---|---|---|
| H | 6.402412 | 10.352870 | 9.970378 |
| C | 3.807121 | 9.823142 | 10.495930 |
| H | 4.464044 | 9.488462 | 11.297283 |
| C | 2.474969 | 9.423946 | 10.488653 |
| H | 2.107805 | 8.781055 | 11.288790 |
| C | -4.781939 | 10.904932 | 12.151506 |
| H | -4.559073 | 11.976619 | 12.126797 |
| H | -4.607225 | 10.554656 | 13.174348 |
| H | -5.846840 | 10.779938 | 11.914019 |
| C | -4.194318 | 8.100463 | 11.109407 |
| H | -5.282420 | 7.985803 | 11.022153 |
| H | -3.892372 | 7.715145 | 12.090047 |
| H | -3.730758 | 7.481945 | 10.333303 |
| C | -4.284875 | 10.425496 | 9.170221 |
| H | -3.774627 | 9.834556 | 8.402720 |
| H | -4.088349 | 11.481125 | 8.960092 |
| H | -5.365229 | 10.256192 | 9.071588 |
| C | -2.383473 | 13.557549 | 10.252102 |
| H | -3.393566 | 13.379674 | 10.634102 |
| H | -2.368802 | 13.255511 | 9.199484 |
| H | -2.194802 | 14.638163 | 10.293824 |
| C | -1.182956 | 13.246652 | 13.052117 |
| H | -0.368942 | 12.809289 | 13.640153 |
| H | -2.128982 | 12.937570 | 13.511076 |
| H | -1.112574 | 14.340467 | 13.105232 |
| C | 0.600875 | 13.175018 | 10.540291 |
| H | 0.750008 | 14.248610 | 10.713761 |
| H | 0.641378 | 13.001874 | 9.459835 |
| H | 1.441241 | 12.636434 | 10.984636 |
| C | 0.730960 | 6.569204 | 15.088531 |
| C | -0.177041 | 5.393728 | 14.749483 |
| H | 0.198838 | 5.027944 | 13.783788 |
| C | 0.028058 | 4.266114 | 15.775749 |
| H | -0.352703 | 4.563137 | 16.759575 |
| H | 1.086795 | 4.010097 | 15.883346 |
| H | -0.517220 | 3.367436 | 15.466040 |
| C | -1.671112 | 5.668914 | 14.551950 |
| H | -2.125989 | 4.804124 | 14.056033 |
| H | -1.841585 | 6.551751 | 13.927768 |
| H | -2.198698 | 5.806060 | 15.501324 |
| C | 2.031371 | 6.525643 | 14.579321 |
| H | 2.304911 | 5.707901 | 13.917141 |
| C | 2.961344 | 7.508345 | 14.879258 |
| H | 3.961883 | 7.451921 | 14.459830 |
| C | 2.619943 | 8.559281 | 15.720361 |
| H | 2.271060 | 10.480395 | 13.882466 |
| H | 3.365265 | 9.307472 | 15.977081 |
| C | 1.333886 | 8.660695 | 16.248627 |
| C | 0.990727 | 9.789534 | 17.204130 |
| H | -0.088403 | 9.774600 | 17.385806 |
| C | 1.320009 | 11.171479 | 16.631561 |
| H | 2.395755 | 11.291368 | 16.457618 |
| H | 1.015843 | 11.948549 | 17.340816 |
| H | 0.794714 | 11.340436 | 15.687093 |
| C | 1.692053 | 9.566432 | 18.552832 |
| H | 2.781213 | 9.600707 | 18.436861 |
| H | 1.430852 | 8.592468 | 18.980136 |
| H | 1.402425 | 10.345282 | 19.266971 |
| C | 0.408329 | 7.662672 | 15.903320 |
| C | -5.160411 | 7.467040 | 15.389296 |
| H | -4.140962 | 7.391326 | 14.989367 |
| C | -6.115249 | 7.416996 | 14.194738 |
| H | -7.160552 | 7.330327 | 14.512404 |
| H | -5.888059 | 6.540619 | 13.579517 |
| H | -6.012226 | 8.305695 | 13.565406 |
| C | -5.408721 | 6.265821 | 16.315214 |
| H | -4.690064 | 6.228650 | 17.139417 |
| H | -5.323117 | 5.329596 | 15.752367 |
| H | -6.416062 | 6.316573 | 16.744513 |
| H | 2.337462 | 11.083106 | 12.379085 |
| H | 2.588795 | 12.073747 | 13.656937 |

Calculated energies and coordinates of **A**

Electronic energy ... -4057.27477323 Eh
Total Enthalpy ... -4056.00789667 Eh
Final Gibbs free energy ... -4056.18573410 Eh

CARTESIAN COORDINATES (ANGSTROEM)

| | | | |
|---|---|---|---|
| Si | -0.507124 | 9.036752 | 9.307383 |
| Si | -1.752095 | 9.886711 | 11.137602 |
| Si | -0.619042 | 8.798916 | 12.959630 |
| P | -1.854156 | 9.803818 | 14.582256 |
| Si | -3.952743 | 9.019889 | 11.146018 |
| Si | -1.877299 | 12.253183 | 11.037670 |
| N | -2.387551 | 8.908225 | 17.142771 |
| N | -0.408059 | 8.213863 | 16.565220 |
| N | 0.986416 | 10.059094 | 12.932240 |
| C | -1.183179 | 12.119219 | 3.937463 |
| H | -0.244548 | 12.561613 | 3.590787 |
| H | -1.930781 | 12.914039 | 4.016197 |
| H | -1.524658 | 11.423248 | 3.161116 |
| C | -1.001590 | 11.405827 | 5.249566 |
| C | 0.220557 | 10.818318 | 5.585115 |
| H | 1.058341 | 10.898024 | 4.895098 |
| C | 0.381035 | 10.133465 | 6.785321 |
| H | 1.345499 | 9.683120 | 7.011062 |
| C | -0.671770 | 10.016666 | 7.703345 |
| C | -1.496834 | 8.918755 | 16.082862 |
| C | -3.673888 | 9.542825 | 17.125801 |
| C | -3.768202 | 10.879315 | 17.533616 |
| C | -2.552435 | 11.697738 | 17.920886 |
| H | -1.660262 | 11.091435 | 17.731729 |
| C | -2.575673 | 12.048088 | 19.414650 |
| H | -3.435992 | 12.682424 | 19.655799 |
| H | -1.667103 | 12.593029 | 19.694598 |
| H | -2.640820 | 11.146665 | 20.033508 |
| C | -6.159325 | 10.730849 | 17.187276 |
| H | -7.140116 | 11.197274 | 17.214874 |
| C | -6.032397 | 9.415678 | 16.757259 |
| H | -6.915812 | 8.869679 | 16.442098 |
| C | -4.785226 | 8.794519 | 16.710212 |
| C | -5.038986 | 11.456176 | 17.564340 |
| H | -5.150148 | 12.490454 | 17.877905 |
| C | -2.431288 | 12.956031 | 17.051659 |

| | | | |
|---|---|---|---|
| H | -2.410132 | 12.685156 | 15.991465 |
| H | -1.506962 | 13.493356 | 17.291821 |
| H | -3.267739 | 13.643088 | 17.220553 |
| C | -1.847730 | 8.257511 | 18.240138 |
| H | -2.391902 | 8.161983 | 19.166438 |
| C | -0.618737 | 7.823505 | 17.884548 |
| H | 0.126677 | 7.273239 | 18.436982 |
| C | -0.979741 | 7.244929 | 8.936827 |
| C | -1.269408 | 6.831137 | 7.629553 |
| H | -1.226311 | 7.551854 | 6.815795 |
| C | -1.622645 | 5.514844 | 7.349513 |
| H | -1.849653 | 5.226476 | 6.325001 |
| C | -1.693680 | 4.557615 | 8.363020 |
| C | -1.412058 | 4.964090 | 9.669492 |
| H | -1.479863 | 4.241758 | 10.481146 |
| C | -1.067057 | 6.280667 | 9.952670 |
| H | -0.886539 | 6.570860 | 10.988736 |
| C | -2.043325 | 3.126966 | 8.055451 |
| H | -1.138990 | 2.539562 | 7.852809 |
| H | -2.685277 | 3.054580 | 7.172465 |
| H | -2.559477 | 2.653715 | 8.896223 |
| C | -2.058346 | 11.286007 | 6.154790 |
| H | -3.020735 | 11.735569 | 5.917866 |
| C | -1.893720 | 10.605226 | 7.355834 |
| H | -2.733498 | 10.528659 | 8.042159 |
| C | 1.321881 | 9.080718 | 9.817326 |
| C | 2.075877 | 10.261390 | 9.704033 |
| H | 1.645202 | 11.127396 | 9.207640 |
| C | 3.366411 | 10.356265 | 10.216401 |
| H | 3.920804 | 11.286751 | 10.108161 |
| C | 3.963562 | 9.269051 | 10.864275 |
| C | 5.348059 | 9.383818 | 11.441941 |
| H | 5.756331 | 8.401184 | 11.695293 |
| H | 5.344454 | 9.991697 | 12.355710 |
| H | 6.033909 | 9.866111 | 10.737705 |
| C | 3.230518 | 8.086397 | 10.963426 |
| H | 3.677865 | 7.220646 | 11.447122 |
| C | 1.936488 | 7.992932 | 10.454240 |
| H | 1.399881 | 7.053487 | 10.552039 |
| C | -5.036667 | 10.057041 | 12.297820 |
| H | -5.105576 | 11.099503 | 11.968967 |
| H | -4.631544 | 10.051528 | 13.316559 |
| H | -6.055290 | 9.648115 | 12.324337 |
| C | -3.971964 | 7.237695 | 11.769269 |
| H | -5.007278 | 6.895194 | 11.892634 |
| H | -3.466325 | 7.158392 | 12.738533 |
| H | -3.472946 | 6.566747 | 11.063523 |
| C | -4.739679 | 9.026100 | 9.423876 |
| H | -4.136644 | 8.452316 | 8.710629 |
| H | -4.860956 | 10.042241 | 9.032048 |
| H | -5.735483 | 8.566749 | 9.467363 |
| C | -3.263140 | 12.899206 | 9.917515 |
| H | -4.235623 | 12.461866 | 10.165498 |
| H | -3.054468 | 12.692567 | 8.863237 |
| H | -3.347822 | 13.987397 | 10.034438 |
| C | -2.185007 | 12.970886 | 12.761087 |
| H | -1.381724 | 12.718838 | 13.462468 |
| H | -3.111233 | 12.582391 | 13.197538 |
| H | -2.261414 | 14.064359 | 12.700901 |
| C | -0.271969 | 13.013234 | 10.375567 |
| H | -0.329166 | 14.108134 | 10.421212 |
| H | -0.117442 | 12.728602 | 9.328337 |
| H | 0.610896 | 12.699927 | 10.944420 |
| C | 1.236139 | 7.047688 | 15.162228 |
| C | 0.440046 | 5.758295 | 14.991885 |
| H | 0.674736 | 5.424262 | 13.971667 |
| C | 0.999661 | 4.708804 | 15.971182 |
| H | 0.798061 | 5.007478 | 17.006817 |
| H | 2.081469 | 4.583737 | 15.860714 |
| H | 0.518049 | 3.739439 | 15.802575 |
| C | -1.086748 | 5.800307 | 15.094937 |
| H | -1.483550 | 4.850514 | 14.718876 |
| H | -1.505845 | 6.606562 | 14.485999 |
| H | -1.430108 | 5.910967 | 16.128475 |
| C | 2.503438 | 7.073965 | 14.566255 |
| H | 2.797124 | 6.239143 | 13.934827 |
| C | 3.376229 | 8.134149 | 14.751797 |
| H | 4.348954 | 8.126660 | 14.267673 |
| C | 3.009241 | 9.204796 | 15.560035 |
| H | 1.696761 | 9.731237 | 13.585590 |
| H | 3.704809 | 10.024167 | 15.719910 |
| C | 1.753586 | 9.235906 | 16.171178 |
| C | 1.385842 | 10.409121 | 17.061899 |
| H | 0.408654 | 10.208995 | 17.511213 |
| C | 1.256437 | 11.711146 | 16.260436 |
| H | 2.186783 | 11.948066 | 15.729931 |
| H | 1.032513 | 12.546825 | 16.932647 |
| H | 0.436070 | 11.632241 | 15.537500 |
| C | 2.391151 | 10.561813 | 18.211815 |
| H | 3.384429 | 10.842708 | 17.845249 |
| H | 2.492131 | 9.628705 | 18.776032 |
| H | 2.057696 | 11.345373 | 18.900526 |
| C | 0.877634 | 8.158086 | 15.940606 |
| C | -4.637204 | 7.362181 | 16.235774 |
| H | -3.616344 | 7.248424 | 15.852396 |
| C | -5.589067 | 7.021125 | 15.085718 |
| H | -6.630086 | 6.956748 | 15.421925 |
| H | -5.321888 | 6.047461 | 14.662248 |
| H | -5.524304 | 7.766718 | 14.288636 |
| C | -4.829728 | 6.377966 | 17.399571 |
| H | -4.110955 | 6.556226 | 18.205459 |
| H | -4.700153 | 5.346602 | 17.052567 |
| H | -5.838311 | 6.474099 | 17.818476 |
| H | 1.407762 | 10.103929 | 12.002576 |
| H | 0.704317 | 10.994351 | 13.215223 |

Calculated energies and coordinates of **TS[A-B]**

Electronic energy ... -4057.25832664 Eh
Total Enthalpy ... -4055.99342011 Eh
Final Gibbs free energy ... -4056.16719886 Eh

CARTESIAN COORDINATES (ANGSTROEM)

| | | | |
|---|---|---|---|
| Si | -0.144050 | 9.119059 | 9.456292 |
| Si | -1.455516 | 10.226803 | 11.095015 |

| | | | |
|---|---|---|---|
| Si | -0.773214 | 9.220743 | 13.185789 |
| P | -2.233062 | 10.024160 | 14.675684 |
| Si | -3.773219 | 9.808650 | 10.798564 |
| Si | -1.101506 | 12.578634 | 11.114495 |
| N | -2.862078 | 8.557147 | 16.962029 |
| N | -0.884876 | 7.999762 | 16.233397 |
| N | 0.801050 | 10.392835 | 13.504503 |
| C | -2.326367 | 9.859399 | 3.673379 |
| H | -1.903831 | 9.125285 | 2.981220 |
| H | -2.116378 | 10.860763 | 3.285358 |
| H | -3.415573 | 9.728291 | 3.668299 |
| C | -1.775944 | 9.683615 | 5.062219 |
| C | -1.317185 | 8.440770 | 5.503014 |
| H | -1.333011 | 7.593486 | 4.820472 |
| C | -0.840384 | 8.269641 | 6.799141 |
| H | -0.491505 | 7.287630 | 7.111186 |
| C | -0.807690 | 9.336683 | 7.705857 |
| C | -1.953058 | 8.817392 | 15.959075 |
| C | -4.062150 | 9.307794 | 17.195604 |
| C | -3.978315 | 10.443720 | 18.011220 |
| C | -2.660811 | 10.951988 | 18.565278 |
| H | -1.854616 | 10.322652 | 18.172524 |
| C | -2.624723 | 10.851664 | 20.096065 |
| H | -3.390214 | 11.488789 | 20.553027 |
| H | -1.648690 | 11.174055 | 20.475476 |
| H | -2.800350 | 9.823670 | 20.430327 |
| C | -6.373818 | 10.690723 | 17.771951 |
| H | -7.287684 | 11.230641 | 18.003764 |
| C | -6.421752 | 9.581180 | 16.935754 |
| H | -7.372859 | 9.274334 | 16.513337 |
| C | -5.265328 | 8.867892 | 16.623178 |
| C | -5.165007 | 11.122687 | 18.295927 |
| H | -5.138126 | 12.004776 | 18.929594 |
| C | -2.384740 | 12.385761 | 18.093197 |
| H | -2.402501 | 12.439106 | 16.999913 |
| H | -1.399047 | 12.714030 | 18.440807 |
| H | -3.128467 | 13.087191 | 18.487117 |
| C | -2.346879 | 7.623810 | 17.850074 |
| H | -2.899922 | 7.306479 | 18.719887 |
| C | -1.130445 | 7.260933 | 17.384277 |
| H | -0.412794 | 6.544818 | 17.752552 |
| C | -0.090870 | 7.267461 | 9.821006 |
| C | -1.294945 | 6.563906 | 9.960133 |
| H | -2.238775 | 7.094987 | 9.857432 |
| C | -1.315818 | 5.197513 | 10.210900 |
| H | -2.268907 | 4.682620 | 10.313695 |
| C | -0.125906 | 4.476210 | 10.341367 |
| C | 1.078701 | 5.164428 | 10.182775 |
| H | 2.018127 | 4.620909 | 10.262712 |
| C | 1.096405 | 6.532226 | 9.923847 |
| H | 2.053862 | 7.031695 | 9.793785 |
| C | -0.138917 | 3.009136 | 10.671247 |
| H | 0.682819 | 2.482010 | 10.176885 |
| H | -1.080593 | 2.540442 | 10.371349 |
| H | -0.021788 | 2.856099 | 11.751399 |
| C | -1.739131 | 10.754328 | 5.959607 |
| H | -2.092073 | 11.733307 | 5.640925 |
| C | -1.270702 | 10.579534 | 7.255611 |
| H | -1.278193 | 11.425637 | 7.938466 |
| C | 1.609030 | 9.818249 | 9.535291 |
| C | 2.116601 | 10.661867 | 8.539987 |
| H | 1.526132 | 10.853786 | 7.646484 |
| C | 3.362120 | 11.269800 | 8.673879 |
| H | 3.725621 | 11.925791 | 7.885423 |
| C | 4.152603 | 11.057855 | 9.804900 |
| C | 5.506839 | 11.698350 | 9.937915 |
| H | 5.751920 | 11.897769 | 10.985400 |
| H | 5.557137 | 12.641480 | 9.386210 |
| H | 6.288644 | 11.040829 | 9.537820 |
| C | 3.662036 | 10.201287 | 10.795250 |
| H | 4.262806 | 10.003878 | 11.681695 |
| C | 2.418994 | 9.593146 | 10.659245 |
| H | 2.084061 | 8.911847 | 11.441368 |
| C | -4.777209 | 11.214628 | 11.563806 |
| H | -4.576926 | 12.179795 | 11.086902 |
| H | -4.561153 | 11.303443 | 12.634360 |
| H | -5.847933 | 11.001487 | 11.450200 |
| C | -4.336639 | 8.232804 | 11.673101 |
| H | -5.401463 | 8.064302 | 11.467145 |
| H | -4.212427 | 8.361135 | 12.753610 |
| H | -3.786546 | 7.335383 | 11.376434 |
| C | -4.226477 | 9.686096 | 8.967483 |
| H | -3.693375 | 8.875949 | 8.458231 |
| H | -3.989067 | 10.610171 | 8.429961 |
| H | -5.304007 | 9.502270 | 8.868545 |
| C | -1.851069 | 13.497000 | 9.631625 |
| H | -2.835435 | 13.110566 | 9.347894 |
| H | -1.198890 | 13.444876 | 8.753319 |
| H | -1.968566 | 14.557497 | 9.888742 |
| C | -1.884882 | 13.311508 | 12.672687 |
| H | -1.570183 | 12.795114 | 13.586992 |
| H | -2.977171 | 13.255107 | 12.642633 |
| H | -1.602157 | 14.368631 | 12.759418 |
| C | 0.736517 | 13.050694 | 11.149886 |
| H | 0.822286 | 14.128035 | 10.957450 |
| H | 1.321511 | 12.526704 | 10.387342 |
| H | 1.208951 | 12.868095 | 12.121388 |
| C | 0.337560 | 7.497199 | 14.135303 |
| C | -0.456907 | 6.215092 | 13.773267 |
| H | -0.307976 | 6.106233 | 12.690173 |
| C | 0.182125 | 5.002124 | 14.464569 |
| H | 0.089111 | 5.086093 | 15.554276 |
| H | 1.247580 | 4.921340 | 14.227048 |
| H | -0.320953 | 4.078583 | 14.155193 |
| C | -1.965811 | 6.208671 | 14.010978 |
| H | -2.413052 | 5.368192 | 13.467341 |
| H | -2.433301 | 7.127834 | 13.643475 |
| H | -2.219909 | 6.087313 | 15.070288 |
| C | 1.690887 | 7.439255 | 13.573115 |
| H | 1.796862 | 6.937716 | 12.613405 |
| C | 2.786713 | 7.974625 | 14.188879 |
| H | 3.755961 | 7.899825 | 13.698234 |
| C | 2.698086 | 8.604776 | 15.450491 |
| H | 1.405862 | 9.999276 | 14.236465 |
| H | 3.577606 | 9.038546 | 15.909701 |
| C | 1.468679 | 8.592333 | 16.123502 |

| | | | |
|---|---|---|---|
| C | 1.341154 | 9.325557 | 17.452623 |
| H | 0.479593 | 8.926434 | 17.997914 |
| C | 1.056213 | 10.814512 | 17.193465 |
| H | 1.897456 | 11.276113 | 16.661221 |
| H | 0.919396 | 11.352078 | 18.139525 |
| H | 0.148318 | 10.944166 | 16.590924 |
| C | 2.560692 | 9.154684 | 18.362878 |
| H | 3.442581 | 9.666427 | 17.964016 |
| H | 2.814665 | 8.097854 | 18.494064 |
| H | 2.352918 | 9.585531 | 19.348498 |
| C | 0.357974 | 7.990483 | 15.516403 |
| C | -5.299482 | 7.658975 | 15.708536 |
| H | -4.339049 | 7.629698 | 15.178877 |
| C | -6.403354 | 7.743991 | 14.651383 |
| H | -7.398982 | 7.619780 | 15.092309 |
| H | -6.269222 | 6.944377 | 13.916063 |
| H | -6.374322 | 8.700610 | 14.120249 |
| C | -5.440480 | 6.358905 | 16.515763 |
| H | -4.613225 | 6.226646 | 17.219012 |
| H | -5.450708 | 5.494338 | 15.842722 |
| H | -6.377113 | 6.361987 | 17.085605 |
| H | 1.370351 | 10.548780 | 12.671342 |
| H | 0.450266 | 11.290140 | 13.832336 |

Calculated energies and coordinates of **B**

Electronic energy ... -4057.25784946 Eh
Total Enthalpy ... -4055.99211103 Eh
Final Gibbs free energy ... -4056.16687333 Eh

CARTESIAN COORDINATES (ANGSTROEM)

| | | | |
|---|---|---|---|
| Si | -0.037509 | 9.062789 | 9.460539 |
| Si | -1.374912 | 10.120677 | 11.116259 |
| Si | -0.773453 | 9.303674 | 13.304887 |
| P | -2.285449 | 10.102018 | 14.724401 |
| Si | -3.694415 | 9.731555 | 10.770581 |
| Si | -1.067441 | 12.485255 | 11.108760 |
| N | -2.916330 | 8.597611 | 16.992181 |
| N | -0.927153 | 8.080510 | 16.255155 |
| N | 0.794264 | 10.448704 | 13.575155 |
| C | -2.496816 | 9.940341 | 3.810562 |
| H | -2.228898 | 9.140168 | 3.114696 |
| H | -2.185992 | 10.894982 | 3.375321 |
| H | -3.590946 | 9.957190 | 3.888767 |
| C | -1.870764 | 9.725144 | 5.161029 |
| C | -1.524671 | 8.446254 | 5.600050 |
| H | -1.683946 | 7.594818 | 4.941615 |
| C | -0.977901 | 8.243187 | 6.863834 |
| H | -0.718742 | 7.233238 | 7.173253 |
| C | -0.760456 | 9.313860 | 7.739239 |
| C | -2.005169 | 8.887832 | 16.002201 |
| C | -4.119564 | 9.338410 | 17.240579 |
| C | -4.043241 | 10.448329 | 18.091823 |
| C | -2.731991 | 10.936285 | 18.677874 |
| H | -1.924384 | 10.300834 | 18.298872 |
| C | -2.729768 | 10.824355 | 20.208027 |
| H | -3.493853 | 11.470623 | 20.654257 |
| H | -1.756786 | 11.127347 | 20.610131 |
| H | -2.929252 | 9.796817 | 20.530048 |
| C | -6.434504 | 10.711041 | 17.828466 |
| H | -7.349681 | 11.247018 | 18.064136 |
| C | -6.475046 | 9.626422 | 16.959729 |
| H | -7.421814 | 9.334576 | 16.517550 |
| C | -5.317062 | 8.918909 | 16.640801 |
| C | -5.231593 | 11.122770 | 18.381022 |
| H | -5.209821 | 11.985291 | 19.041233 |
| C | -2.430259 | 12.370667 | 18.222833 |
| H | -2.431555 | 12.435794 | 17.130015 |
| H | -1.445356 | 12.682753 | 18.587247 |
| H | -3.171419 | 13.077449 | 18.612229 |
| C | -2.390723 | 7.651119 | 17.860722 |
| H | -2.940374 | 7.311778 | 18.724392 |
| C | -1.170477 | 7.310546 | 17.386194 |
| H | -0.450510 | 6.586628 | 17.733448 |
| C | 0.032872 | 7.210536 | 9.798533 |
| C | -1.162583 | 6.488272 | 9.916550 |
| H | -2.113904 | 7.003555 | 9.798740 |
| C | -1.164932 | 5.121920 | 10.169243 |
| H | -2.110818 | 4.590992 | 10.255160 |
| C | 0.034293 | 4.422117 | 10.326653 |
| C | 1.230218 | 5.130223 | 10.190402 |
| H | 2.176776 | 4.603908 | 10.294896 |
| C | 1.230298 | 6.496481 | 9.926204 |
| H | 2.181018 | 7.014403 | 9.819982 |
| C | 0.040074 | 2.957042 | 10.664395 |
| H | 0.877204 | 2.440521 | 10.185025 |
| H | -0.889512 | 2.471374 | 10.354169 |
| H | 0.144382 | 2.813469 | 11.747028 |
| C | -1.643477 | 10.800347 | 6.024941 |
| H | -1.900545 | 11.808582 | 5.705783 |
| C | -1.105545 | 10.594577 | 7.288207 |
| H | -0.957355 | 11.449450 | 7.943387 |
| C | 1.698882 | 9.802024 | 9.508717 |
| C | 2.205407 | 10.587585 | 8.466453 |
| H | 1.622229 | 10.718497 | 7.557766 |
| C | 3.444347 | 11.214836 | 8.571702 |
| H | 3.806894 | 11.824913 | 7.746847 |
| C | 4.229416 | 11.079070 | 9.717700 |
| C | 5.576407 | 11.739699 | 9.821314 |
| H | 5.800688 | 12.026558 | 10.853082 |
| H | 5.631331 | 12.634460 | 9.194669 |
| H | 6.369599 | 11.057174 | 9.491848 |
| C | 3.740987 | 10.277585 | 10.754044 |
| H | 4.337449 | 10.137892 | 11.654178 |
| C | 2.504431 | 9.652220 | 10.648286 |
| H | 2.171588 | 9.015241 | 11.467605 |
| C | -4.702300 | 11.134837 | 11.534778 |
| H | -4.480424 | 12.108612 | 11.086251 |
| H | -4.518452 | 11.197681 | 12.613213 |
| H | -5.770875 | 10.933942 | 11.384154 |
| C | -4.296362 | 8.148819 | 11.602845 |
| H | -5.342784 | 7.968302 | 11.325380 |
| H | -4.249966 | 8.275849 | 12.689074 |
| H | -3.715309 | 7.259416 | 11.343006 |
| C | -4.107043 | 9.639871 | 8.928780 |

| | | | |
|---|---|---|---|
| H | -3.592689 | 8.813789 | 8.426150 |
| H | -3.828448 | 10.556366 | 8.398693 |
| H | -5.188178 | 9.492944 | 8.809646 |
| C | -1.925891 | 13.345003 | 9.647826 |
| H | -2.832792 | 12.826872 | 9.321577 |
| H | -1.258911 | 13.434011 | 8.783950 |
| H | -2.208767 | 14.361880 | 9.947220 |
| C | -1.805240 | 13.235286 | 12.681266 |
| H | -1.402984 | 12.801354 | 13.603404 |
| H | -2.890030 | 13.099197 | 12.724116 |
| H | -1.597544 | 14.313194 | 12.693538 |
| C | 0.751691 | 13.017659 | 11.050184 |
| H | 0.787919 | 14.103656 | 10.893106 |
| H | 1.305721 | 12.541558 | 10.234813 |
| H | 1.294991 | 12.817323 | 11.980263 |
| C | 0.160582 | 7.665344 | 14.046165 |
| C | -0.610806 | 6.346997 | 13.729722 |
| H | -0.517482 | 6.238786 | 12.639536 |
| C | 0.086128 | 5.149164 | 14.382372 |
| H | 0.063636 | 5.239606 | 15.475351 |
| H | 1.135309 | 5.082508 | 14.078427 |
| H | -0.422392 | 4.217210 | 14.108855 |
| C | -2.105684 | 6.317416 | 14.047685 |
| H | -2.579162 | 5.483009 | 13.516725 |
| H | -2.601129 | 7.239305 | 13.728670 |
| H | -2.298122 | 6.175096 | 15.117346 |
| C | 1.533600 | 7.559996 | 13.449825 |
| H | 1.592369 | 7.066265 | 12.483035 |
| C | 2.661780 | 8.009748 | 14.061051 |
| H | 3.615159 | 7.891580 | 13.546361 |
| C | 2.643775 | 8.617602 | 15.344464 |
| H | 1.418287 | 10.058099 | 14.303328 |
| H | 3.559304 | 8.981136 | 15.791990 |
| C | 1.441990 | 8.594287 | 16.070662 |
| C | 1.388989 | 9.263354 | 17.439529 |
| H | 0.579223 | 8.813984 | 18.023514 |
| C | 1.043088 | 10.753104 | 17.268844 |
| H | 1.842010 | 11.266055 | 16.718597 |
| H | 0.937694 | 11.242625 | 18.244737 |
| H | 0.104084 | 10.882008 | 16.716431 |
| C | 2.670602 | 9.095361 | 18.259408 |
| H | 3.506352 | 9.655820 | 17.827950 |
| H | 2.967332 | 8.043625 | 18.323227 |
| H | 2.514975 | 9.474097 | 19.275753 |
| C | 0.285524 | 8.066723 | 15.490869 |
| C | -5.342849 | 7.735048 | 15.694021 |
| H | -4.376803 | 7.719479 | 15.175233 |
| C | -6.433412 | 7.848374 | 14.626181 |
| H | -7.435674 | 7.727025 | 15.052426 |
| H | -6.299078 | 7.059752 | 13.879021 |
| H | -6.388267 | 8.813834 | 14.111895 |
| C | -5.488622 | 6.413887 | 16.464919 |
| H | -4.666484 | 6.265838 | 17.171176 |
| H | -5.488662 | 5.568010 | 15.768462 |
| H | -6.430521 | 6.397841 | 17.025766 |
| H | 1.349445 | 10.572223 | 12.726884 |
| H | 0.469397 | 11.361545 | 13.887240 |

Calculated energies and coordinates of **TS[B-2]**

Electronic energy ... -4057.25284936 Eh
Total Enthalpy ... -4055.99202728 Eh
Final Gibbs free energy ... -4056.16469054 Eh

CARTESIAN COORDINATES (ANGSTROEM)

| | | | |
|---|---|---|---|
| Si | 0.000836 | 9.003656 | 9.479549 |
| Si | -1.329327 | 10.032833 | 11.169607 |
| Si | -0.602708 | 9.505913 | 13.406687 |
| P | -2.249158 | 10.153861 | 14.768986 |
| Si | -3.660625 | 9.667213 | 10.836354 |
| Si | -1.121708 | 12.405737 | 11.006393 |
| N | -2.950561 | 8.517744 | 16.931499 |
| N | -0.947010 | 8.049582 | 16.212843 |
| N | 0.846914 | 10.677243 | 13.704912 |
| C | -2.632387 | 10.100343 | 3.947158 |
| H | -3.662201 | 10.442043 | 4.108447 |
| H | -2.674966 | 9.207503 | 3.317136 |
| H | -2.111468 | 10.888156 | 3.393792 |
| C | -1.956067 | 9.814988 | 5.259612 |
| C | -1.836448 | 8.512614 | 5.744826 |
| H | -2.211368 | 7.684077 | 5.147629 |
| C | -1.242729 | 8.255871 | 6.977840 |
| H | -1.159685 | 7.227038 | 7.320170 |
| C | -0.750090 | 9.294384 | 7.775815 |
| C | -2.017075 | 8.873822 | 15.986763 |
| C | -4.146815 | 9.258289 | 17.213019 |
| C | -4.062419 | 10.321926 | 18.121276 |
| C | -2.751061 | 10.761180 | 18.744613 |
| H | -1.948302 | 10.132135 | 18.345040 |
| C | -2.767586 | 10.576312 | 20.267656 |
| H | -3.526464 | 11.212284 | 20.736867 |
| H | -1.794390 | 10.846407 | 20.692125 |
| H | -2.984414 | 9.537776 | 20.539319 |
| C | -6.447369 | 10.632463 | 17.851151 |
| H | -7.356818 | 11.169484 | 18.105758 |
| C | -6.495656 | 9.593411 | 16.929244 |
| H | -7.442992 | 9.337334 | 16.466681 |
| C | -5.344771 | 8.885988 | 16.584435 |
| C | -5.243719 | 10.997788 | 18.434083 |
| H | -5.215905 | 11.824962 | 19.137794 |
| C | -2.422503 | 12.210945 | 18.362427 |
| H | -2.394332 | 12.323459 | 17.273993 |
| H | -1.444060 | 12.493286 | 18.765941 |
| H | -3.165860 | 12.908007 | 18.764822 |
| C | -2.445737 | 7.506768 | 17.742137 |
| H | -3.013744 | 7.111521 | 18.569127 |
| C | -1.210372 | 7.204095 | 17.278935 |
| H | -0.485968 | 6.476841 | 17.609610 |
| C | 0.081371 | 7.151328 | 9.815427 |
| C | -1.099714 | 6.403100 | 9.911550 |
| H | -2.057943 | 6.891067 | 9.745053 |
| C | -1.080045 | 5.049826 | 10.227277 |
| H | -2.015892 | 4.499637 | 10.301386 |
| C | 0.127175 | 4.390617 | 10.471668 |
| C | 1.310107 | 5.125402 | 10.363201 |

| | | | |
|---|---|---|---|
| H | 2.262781 | 4.633019 | 10.548118 |
| C | 1.289148 | 6.476899 | 10.034524 |
| H | 2.229140 | 7.019685 | 9.961931 |
| C | 0.157646 | 2.929074 | 10.824616 |
| H | -0.812777 | 2.589115 | 11.197236 |
| H | 0.911256 | 2.724193 | 11.591442 |
| H | 0.408958 | 2.320092 | 9.947467 |
| C | -1.441751 | 10.855959 | 6.039187 |
| H | -1.505110 | 11.879568 | 5.674758 |
| C | -0.854833 | 10.598122 | 7.270137 |
| H | -0.455431 | 11.430056 | 7.846823 |
| C | 1.740221 | 9.735762 | 9.492897 |
| C | 2.331463 | 10.300637 | 8.356381 |
| H | 1.802698 | 10.283420 | 7.406525 |
| C | 3.593868 | 10.885244 | 8.414743 |
| H | 4.025781 | 11.316280 | 7.513840 |
| C | 4.319024 | 10.923697 | 9.606715 |
| C | 5.665381 | 11.590036 | 9.677108 |
| H | 5.565805 | 12.633002 | 10.002466 |
| H | 6.158696 | 11.595904 | 8.700934 |
| H | 6.321874 | 11.086139 | 10.392972 |
| C | 3.748695 | 10.333008 | 10.738112 |
| H | 4.301216 | 10.318555 | 11.676223 |
| C | 2.486220 | 9.756402 | 10.679856 |
| H | 2.094589 | 9.274609 | 11.575206 |
| C | -4.664402 | 10.962616 | 11.778189 |
| H | -4.471750 | 11.987060 | 11.447972 |
| H | -4.457087 | 10.901633 | 12.852369 |
| H | -5.732411 | 10.759552 | 11.623905 |
| C | -4.318525 | 8.006561 | 11.450690 |
| H | -5.357675 | 7.899561 | 11.112986 |
| H | -4.319450 | 7.989737 | 12.544015 |
| H | -3.756057 | 7.137140 | 11.100791 |
| C | -4.066835 | 9.805671 | 8.995396 |
| H | -3.630532 | 8.984642 | 8.416793 |
| H | -3.698381 | 10.738405 | 8.557066 |
| H | -5.156103 | 9.773661 | 8.863921 |
| C | -2.220494 | 13.134798 | 9.645958 |
| H | -3.282919 | 12.984602 | 9.858783 |
| H | -2.022167 | 12.712854 | 8.657432 |
| H | -2.042087 | 14.216580 | 9.593835 |
| C | -1.676236 | 13.248841 | 12.609172 |
| H | -1.001767 | 13.069389 | 13.452487 |
| H | -2.671718 | 12.921657 | 12.924796 |
| H | -1.712233 | 14.333690 | 12.445306 |
| C | 0.649233 | 12.971933 | 10.648094 |
| H | 0.663124 | 14.065894 | 10.559397 |
| H | 1.044208 | 12.549567 | 9.718282 |
| H | 1.353514 | 12.699889 | 11.441737 |
| C | 0.244293 | 7.861152 | 14.018399 |
| C | -0.470105 | 6.536762 | 13.615237 |
| H | -0.286498 | 6.457145 | 12.535285 |
| C | 0.186091 | 5.325391 | 14.284154 |
| H | 0.026884 | 5.346149 | 15.369136 |
| H | 1.265721 | 5.302206 | 14.108648 |
| H | -0.251231 | 4.397405 | 13.897247 |
| C | -1.987139 | 6.477602 | 13.798759 |
| H | -2.395784 | 5.656173 | 13.198327 |
| H | -2.469418 | 7.403134 | 13.471188 |
| H | -2.274043 | 6.295864 | 14.841032 |
| C | 1.674139 | 7.783411 | 13.513039 |
| H | 1.836330 | 7.180917 | 12.623402 |
| C | 2.699289 | 8.388819 | 14.140713 |
| H | 3.704134 | 8.304911 | 13.728492 |
| C | 2.482264 | 9.215673 | 15.312464 |
| H | 1.664866 | 10.239143 | 14.493018 |
| H | 3.360904 | 9.570763 | 15.845508 |
| C | 1.321907 | 8.868076 | 16.099309 |
| C | 1.227098 | 9.409676 | 17.512973 |
| H | 0.278136 | 9.084636 | 17.952857 |
| C | 1.213513 | 10.945685 | 17.499953 |
| H | 2.152634 | 11.347639 | 17.105148 |
| H | 1.079878 | 11.331806 | 18.517463 |
| H | 0.388016 | 11.318753 | 16.882182 |
| C | 2.349665 | 8.871469 | 18.409140 |
| H | 3.331345 | 9.224088 | 18.077459 |
| H | 2.367641 | 7.776471 | 18.399307 |
| H | 2.203925 | 9.205312 | 19.442833 |
| C | 0.293534 | 8.187426 | 15.509774 |
| C | -5.384663 | 7.743628 | 15.588201 |
| H | -4.402343 | 7.702867 | 15.101099 |
| C | -6.433694 | 7.946164 | 14.492192 |
| H | -7.453547 | 7.879659 | 14.886904 |
| H | -6.329492 | 7.164098 | 13.733598 |
| H | -6.313489 | 8.916407 | 13.999223 |
| C | -5.618946 | 6.403976 | 16.304149 |
| H | -4.842898 | 6.200220 | 17.047833 |
| H | -5.616438 | 5.581353 | 15.580474 |
| H | -6.588391 | 6.408190 | 16.816222 |
| H | 1.365401 | 10.884289 | 12.851583 |
| H | 0.492403 | 11.559030 | 14.069284 |

Calculated energies and coordinates of **2**

Electronic energy ... -4057.29904654 Eh
Total Enthalpy ... -4056.03316380 Eh
Final Gibbs free energy ... -4056.20766428 Eh

CARTESIAN COORDINATES (ANGSTROEM)

| | | | |
|---|---|---|---|
| Si | 0.023032 | 9.015486 | 9.522998 |
| Si | -1.373038 | 9.970634 | 11.207997 |
| Si | -0.608879 | 9.718950 | 13.470897 |
| P | -2.288236 | 10.296510 | 14.844037 |
| Si | -3.673914 | 9.474351 | 10.820846 |
| Si | -1.313135 | 12.346326 | 10.943594 |
| N | -2.984489 | 8.642385 | 16.977173 |
| N | -1.006685 | 8.127333 | 16.221141 |
| N | 0.693967 | 10.859552 | 13.746070 |
| C | -2.550328 | 10.083375 | 3.953400 |
| H | -3.591747 | 10.395947 | 4.099062 |
| H | -2.558445 | 9.194843 | 3.315777 |
| H | -2.041114 | 10.889244 | 3.415590 |
| C | -1.890049 | 9.804751 | 5.275480 |
| C | -1.779150 | 8.504867 | 5.769406 |
| H | -2.147145 | 7.673430 | 5.171823 |

| | | | |
|---|---|---|---|
| C | -1.202432 | 8.254458 | 7.011511 |
| H | -1.124362 | 7.226873 | 7.358705 |
| C | -0.717733 | 9.295951 | 7.810387 |
| C | -2.057700 | 8.991354 | 16.016507 |
| C | -4.155830 | 9.411044 | 17.282561 |
| C | -4.029738 | 10.467311 | 18.194450 |
| C | -2.697597 | 10.862981 | 18.803002 |
| H | -1.917559 | 10.224207 | 18.374214 |
| C | -2.693148 | 10.644111 | 20.321835 |
| H | -3.432217 | 11.284038 | 20.816650 |
| H | -1.708418 | 10.886183 | 20.736655 |
| H | -2.926444 | 9.604284 | 20.574503 |
| C | -6.408788 | 10.843998 | 17.958550 |
| H | -7.299461 | 11.404650 | 18.228171 |
| C | -6.498289 | 9.811744 | 17.032282 |
| H | -7.458388 | 9.583896 | 16.580932 |
| C | -5.371604 | 9.075739 | 16.667879 |
| C | -5.187594 | 11.173211 | 18.527085 |
| H | -5.128121 | 11.995946 | 19.234079 |
| C | -2.340144 | 12.312333 | 18.447523 |
| H | -2.329827 | 12.448668 | 17.361576 |
| H | -1.347341 | 12.560964 | 18.838254 |
| H | -3.057215 | 13.018369 | 18.880570 |
| C | -2.502465 | 7.599420 | 17.757920 |
| H | -3.074853 | 7.196339 | 18.578014 |
| C | -1.284772 | 7.267096 | 17.275208 |
| H | -0.589083 | 6.498624 | 17.572873 |
| C | 0.198033 | 7.157742 | 9.802654 |
| C | -0.936677 | 6.340435 | 9.894059 |
| H | -1.924520 | 6.779275 | 9.768782 |
| C | -0.833533 | 4.978083 | 10.150531 |
| H | -1.734906 | 4.371989 | 10.216561 |
| C | 0.414472 | 4.378746 | 10.337417 |
| C | 1.551761 | 5.182047 | 10.228013 |
| H | 2.535342 | 4.734438 | 10.356447 |
| C | 1.446586 | 6.543225 | 9.960879 |
| H | 2.352591 | 7.140813 | 9.881162 |
| C | 0.533734 | 2.917960 | 10.674048 |
| H | 1.390488 | 2.461797 | 10.168356 |
| H | -0.367580 | 2.367419 | 10.390221 |
| H | 0.681042 | 2.781213 | 11.752645 |
| C | -1.381818 | 10.848392 | 6.055080 |
| H | -1.436493 | 11.869962 | 5.683644 |
| C | -0.809554 | 10.596523 | 7.294484 |
| H | -0.410907 | 11.430522 | 7.868468 |
| C | 1.741885 | 9.800826 | 9.524431 |
| C | 2.367590 | 10.208224 | 8.339455 |
| H | 1.862537 | 10.068262 | 7.386882 |
| C | 3.630866 | 10.793556 | 8.353312 |
| H | 4.088580 | 11.101142 | 7.415335 |
| C | 4.322366 | 10.990108 | 9.549380 |
| C | 5.670099 | 11.657340 | 9.570069 |
| H | 5.572849 | 12.720936 | 9.820543 |
| H | 6.163056 | 11.591534 | 8.596013 |
| H | 6.326405 | 11.205105 | 10.320221 |
| C | 3.714647 | 10.564290 | 10.733827 |
| H | 4.236133 | 10.687486 | 11.681575 |
| C | 2.451560 | 9.987285 | 10.718437 |

| | | | |
|---|---|---|---|
| H | 2.022958 | 9.655622 | 11.660061 |
| C | -4.751797 | 10.716404 | 11.752977 |
| H | -4.613141 | 11.746684 | 11.413983 |
| H | -4.538091 | 10.679212 | 12.827288 |
| H | -5.807209 | 10.453927 | 11.601610 |
| C | -4.305459 | 7.775717 | 11.368457 |
| H | -5.282890 | 7.608836 | 10.897187 |
| H | -4.448065 | 7.747717 | 12.451840 |
| H | -3.652196 | 6.942474 | 11.096920 |
| C | -4.039796 | 9.610834 | 8.970802 |
| H | -3.612116 | 8.771136 | 8.413093 |
| H | -3.644694 | 10.527356 | 8.523407 |
| H | -5.127222 | 9.597679 | 8.821305 |
| C | -2.448937 | 12.973557 | 9.560171 |
| H | -3.503214 | 12.778037 | 9.777163 |
| H | -2.228043 | 12.536565 | 8.582849 |
| H | -2.324350 | 14.061207 | 9.479217 |
| C | -1.907243 | 13.206355 | 12.519678 |
| H | -1.212574 | 13.061530 | 13.352032 |
| H | -2.887223 | 12.845768 | 12.847717 |
| H | -1.989284 | 14.284458 | 12.329972 |
| C | 0.422167 | 13.000697 | 10.562300 |
| H | 0.372721 | 14.091791 | 10.452905 |
| H | 0.845238 | 12.585690 | 9.641675 |
| H | 1.131012 | 12.779520 | 11.366268 |
| C | 0.129761 | 7.964660 | 14.024928 |
| C | -0.658250 | 6.698905 | 13.567024 |
| H | -0.562257 | 6.704210 | 12.472701 |
| C | 0.010770 | 5.420427 | 14.083691 |
| H | -0.037207 | 5.374083 | 15.179353 |
| H | 1.063565 | 5.364078 | 13.790508 |
| H | -0.506176 | 4.537907 | 13.689321 |
| C | -2.156280 | 6.653867 | 13.871217 |
| H | -2.628241 | 5.888299 | 13.244870 |
| H | -2.637830 | 7.610998 | 13.656001 |
| H | -2.359453 | 6.390886 | 14.915185 |
| C | 1.494265 | 7.883460 | 13.418111 |
| H | 1.526293 | 7.523267 | 12.391507 |
| C | 2.625658 | 8.189225 | 14.046766 |
| H | 3.575739 | 8.098413 | 13.522112 |
| C | 2.665119 | 8.666723 | 15.460789 |
| H | 3.030808 | 9.706751 | 15.493734 |
| H | 3.410867 | 8.085649 | 16.026553 |
| C | 1.336585 | 8.596595 | 16.164416 |
| C | 1.295547 | 9.144140 | 17.580306 |
| H | 0.404761 | 8.761191 | 18.088499 |
| C | 1.149144 | 10.675952 | 17.523701 |
| H | 2.005125 | 11.139242 | 17.020768 |
| H | 1.090709 | 11.087557 | 18.538552 |
| H | 0.242085 | 10.955972 | 16.976990 |
| C | 2.508953 | 8.741175 | 18.424691 |
| H | 3.431242 | 9.201538 | 18.054502 |
| H | 2.650399 | 7.654578 | 18.433102 |
| H | 2.369512 | 9.072769 | 19.458986 |
| C | 0.236822 | 8.184698 | 15.507934 |
| C | -5.456304 | 7.940013 | 15.667309 |
| H | -4.473218 | 7.853706 | 15.189389 |
| C | -6.483070 | 8.194669 | 14.560942 |

| | | | |
|---|---|---|---|
| H | -7.509100 | 8.181610 | 14.944867 |
| H | -6.413288 | 7.405957 | 13.804955 |
| H | -6.307991 | 9.156185 | 14.068164 |
| C | -5.759545 | 6.611981 | 16.379136 |
| H | -5.002600 | 6.375716 | 17.133057 |
| H | -5.783981 | 5.789448 | 15.655712 |
| H | -6.733964 | 6.659018 | 16.879429 |
| H | 1.267952 | 11.174306 | 12.976502 |
| H | 0.459685 | 11.634772 | 14.350064 |

Calculated energies and coordinates of **TS[1-C]**

Electronic energy ... -4000.69175512 Eh
Total Enthalpy ... -3999.46776129 Eh
Final Gibbs free energy ... -3999.63938528 Eh

CARTESIAN COORDINATES (ANGSTROEM)

| | | | |
|---|---|---|---|
| Si | -0.132206 | 9.116613 | 9.511298 |
| Si | -1.559287 | 9.947832 | 11.212405 |
| Si | -1.761486 | 8.459467 | 13.075547 |
| P | -2.508318 | 9.784968 | 14.671364 |
| Si | -3.789884 | 9.864307 | 10.380893 |
| Si | -1.089802 | 12.220102 | 11.725122 |
| N | -2.781675 | 8.647831 | 17.231135 |
| N | -0.953928 | 7.979989 | 16.246087 |
| C | -2.665963 | 10.201038 | 3.926327 |
| H | -3.671550 | 10.616581 | 4.066424 |
| H | -2.765272 | 9.289656 | 3.329991 |
| H | -2.090663 | 10.930590 | 3.347608 |
| C | -2.016377 | 9.920992 | 5.253724 |
| C | -2.000386 | 8.634866 | 5.794354 |
| H | -2.438249 | 7.813034 | 5.231604 |
| C | -1.424823 | 8.386510 | 7.037551 |
| H | -1.412457 | 7.368186 | 7.420941 |
| C | -0.851616 | 9.416543 | 7.792155 |
| C | -2.096223 | 8.728378 | 16.043530 |
| C | -4.016982 | 9.326267 | 17.493719 |
| C | -3.957607 | 10.626872 | 18.013698 |
| C | -2.642977 | 11.330724 | 18.292308 |
| H | -1.826100 | 10.681008 | 17.958795 |
| C | -2.459286 | 11.570354 | 19.797541 |
| H | -3.229634 | 12.245395 | 20.186563 |
| H | -1.482340 | 12.025199 | 19.994623 |
| H | -2.521794 | 10.632099 | 20.359063 |
| C | -6.379030 | 10.640707 | 18.014679 |
| H | -7.311311 | 11.159375 | 18.219526 |
| C | -6.405180 | 9.356128 | 17.488901 |
| H | -7.359623 | 8.880225 | 17.282805 |
| C | -5.222258 | 8.668431 | 17.215745 |
| C | -5.168532 | 11.268983 | 18.274004 |
| H | -5.162873 | 12.277635 | 18.677305 |
| C | -2.530259 | 12.641111 | 17.502487 |
| H | -2.661868 | 12.458719 | 16.431340 |
| H | -1.542143 | 13.088582 | 17.656670 |
| H | -3.281779 | 13.369498 | 17.826609 |
| C | -2.074859 | 7.894938 | 18.150440 |
| H | -2.457720 | 7.706871 | 19.141321 |
| C | -0.941739 | 7.474139 | 17.538626 |
| H | -0.148773 | 6.831146 | 17.886519 |
| C | 0.149816 | 7.261950 | 9.720507 |
| C | -0.899261 | 6.394194 | 10.055296 |
| H | -1.904959 | 6.786210 | 10.181880 |
| C | -0.679391 | 5.034770 | 10.251023 |
| H | -1.515193 | 4.387239 | 10.509557 |
| C | 0.601935 | 4.490317 | 10.136530 |
| C | 1.650814 | 5.347232 | 9.793310 |
| H | 2.656908 | 4.945495 | 9.691369 |
| C | 1.428507 | 6.704970 | 9.585353 |
| H | 2.269164 | 7.347259 | 9.330910 |
| C | 0.848847 | 3.032533 | 10.412665 |
| H | 1.726611 | 2.666745 | 9.872058 |
| H | -0.012553 | 2.421382 | 10.126789 |
| H | 1.028082 | 2.866544 | 11.482601 |
| C | -1.426441 | 10.951543 | 5.992235 |
| H | -1.416005 | 11.961621 | 5.587171 |
| C | -0.861530 | 10.704853 | 7.236612 |
| H | -0.415578 | 11.531483 | 7.785639 |
| C | 1.544968 | 9.976692 | 9.587680 |
| C | 2.296130 | 10.243566 | 8.435689 |
| H | 1.893189 | 9.986005 | 7.458514 |
| C | 3.553028 | 10.836134 | 8.516027 |
| H | 4.113970 | 11.032311 | 7.604562 |
| C | 4.107268 | 11.183438 | 9.749637 |
| C | 5.448771 | 11.858511 | 9.836451 |
| H | 5.331696 | 12.946127 | 9.920474 |
| H | 6.053467 | 11.660051 | 8.947053 |
| H | 6.007205 | 11.522480 | 10.715683 |
| C | 3.366565 | 10.911418 | 10.903096 |
| H | 3.777944 | 11.169129 | 11.877736 |
| C | 2.110312 | 10.323499 | 10.821102 |
| H | 1.558577 | 10.124912 | 11.737259 |
| C | -5.040726 | 10.183076 | 11.760135 |
| H | -4.882126 | 11.140209 | 12.265081 |
| H | -4.987484 | 9.401124 | 12.524628 |
| H | -6.054404 | 10.181875 | 11.339092 |
| C | -4.225580 | 8.175123 | 9.651579 |
| H | -5.260542 | 8.198925 | 9.286952 |
| H | -4.159360 | 7.383965 | 10.407061 |
| H | -3.581930 | 7.911989 | 8.806034 |
| C | -4.041603 | 11.154651 | 9.023609 |
| H | -3.411792 | 10.940956 | 8.154075 |
| H | -3.808143 | 12.167515 | 9.368207 |
| H | -5.089697 | 11.144769 | 8.698072 |
| C | -0.532017 | 13.194103 | 10.202617 |
| H | -1.293001 | 13.183416 | 9.414654 |
| H | 0.398398 | 12.786517 | 9.791257 |
| H | -0.348360 | 14.240451 | 10.478542 |
| C | -2.653617 | 13.038562 | 12.403546 |
| H | -3.011734 | 12.521130 | 13.300401 |
| H | -3.464997 | 13.050469 | 11.668787 |
| H | -2.431823 | 14.078127 | 12.676711 |
| C | 0.239820 | 12.368429 | 13.058553 |
| H | 0.156723 | 13.339524 | 13.562460 |
| H | 1.246874 | 12.289010 | 12.637893 |
| H | 0.113326 | 11.584918 | 13.811744 |

| | | | |
|---|---|---|---|
| C | -0.176790 | 7.149985 | 14.064229 |
| C | -0.854688 | 5.757635 | 14.111044 |
| H | -1.011214 | 5.499832 | 13.054707 |
| C | 0.137842 | 4.747107 | 14.707716 |
| H | 0.349166 | 4.984259 | 15.757385 |
| H | 1.088376 | 4.758344 | 14.164369 |
| H | -0.277576 | 3.733776 | 14.668204 |
| C | -2.207684 | 5.643118 | 14.811367 |
| H | -2.657154 | 4.673413 | 14.569906 |
| H | -2.896407 | 6.425142 | 14.470556 |
| H | -2.115339 | 5.696843 | 15.901157 |
| C | 0.892407 | 7.225948 | 13.096920 |
| H | 0.771259 | 6.670118 | 12.172954 |
| C | 2.040191 | 7.944581 | 13.315168 |
| H | 2.801019 | 7.972451 | 12.539415 |
| C | 2.246978 | 8.626034 | 14.520658 |
| H | 3.158046 | 9.195598 | 14.667237 |
| C | 1.270821 | 8.606137 | 15.519036 |
| C | 1.459285 | 9.451955 | 16.769960 |
| H | 0.808190 | 9.063795 | 17.560135 |
| C | 1.023196 | 10.899821 | 16.500512 |
| H | 1.660452 | 11.357945 | 15.735928 |
| H | 1.098640 | 11.497722 | 17.416786 |
| H | -0.010401 | 10.941277 | 16.138135 |
| C | 2.890940 | 9.402960 | 17.314519 |
| H | 3.598592 | 9.909683 | 16.650302 |
| H | 3.233684 | 8.371996 | 17.451223 |
| H | 2.935477 | 9.912811 | 18.282780 |
| C | 0.100123 | 7.873248 | 15.290242 |
| C | -5.265922 | 7.277366 | 16.616261 |
| H | -4.240896 | 6.896115 | 16.560767 |
| C | -5.813872 | 7.324386 | 15.182943 |
| H | -6.854095 | 7.668994 | 15.170126 |
| H | -5.780848 | 6.327085 | 14.730109 |
| H | -5.218551 | 8.005950 | 14.567471 |
| C | -6.072126 | 6.307913 | 17.490064 |
| H | -5.687409 | 6.281535 | 18.515058 |
| H | -6.018381 | 5.295357 | 17.075920 |
| H | -7.129132 | 6.591870 | 17.534847 |

Calculated energies and coordinates of **C**

Electronic energy ... -4000.69366596 Eh
Total Enthalpy ... -3999.46913916 Eh
Final Gibbs free energy ... -3999.64150517 Eh

CARTESIAN COORDINATES (ANGSTROEM)

| | | | |
|---|---|---|---|
| Si | -0.197257 | 9.041171 | 9.546922 |
| Si | -1.237618 | 10.283708 | 11.291145 |
| Si | -1.392632 | 8.918484 | 13.206173 |
| P | -2.691110 | 9.825262 | 14.720996 |
| Si | -3.522186 | 10.607566 | 10.666333 |
| Si | -0.323994 | 12.438385 | 11.702280 |
| N | -2.860909 | 8.387651 | 17.127618 |
| N | -0.857544 | 8.278954 | 16.257296 |
| C | -3.083934 | 10.065627 | 4.124522 |
| H | -3.968297 | 10.692628 | 4.292316 |
| H | -3.420144 | 9.139612 | 3.649771 |
| H | -2.436778 | 10.598466 | 3.420658 |
| C | -2.372273 | 9.790641 | 5.420795 |
| C | -2.547321 | 8.584903 | 6.100357 |
| H | -3.186873 | 7.816921 | 5.670354 |
| C | -1.906836 | 8.345755 | 7.313629 |
| H | -2.047734 | 7.385452 | 7.805426 |
| C | -1.074800 | 9.307098 | 7.896865 |
| C | -2.124846 | 8.773864 | 16.033054 |
| C | -4.229626 | 8.751512 | 17.342412 |
| C | -4.493769 | 9.938263 | 18.041011 |
| C | -3.387768 | 10.850310 | 18.537975 |
| H | -2.424713 | 10.420028 | 18.240974 |
| C | -3.398741 | 10.952343 | 20.069038 |
| H | -4.322492 | 11.419699 | 20.427724 |
| H | -2.557944 | 11.563202 | 20.415268 |
| H | -3.320735 | 9.963849 | 20.534089 |
| C | -6.853583 | 9.460883 | 17.782383 |
| H | -7.888989 | 9.739466 | 17.957071 |
| C | -6.560408 | 8.300825 | 17.077825 |
| H | -7.370355 | 7.682017 | 16.701889 |
| C | -5.239441 | 7.920155 | 16.839672 |
| C | -5.831432 | 10.271654 | 18.256950 |
| H | -6.075489 | 11.183195 | 18.795247 |
| C | -3.481142 | 12.236711 | 17.886456 |
| H | -3.468146 | 12.151261 | 16.795477 |
| H | -2.630290 | 12.854705 | 18.193950 |
| H | -4.399267 | 12.756177 | 18.182629 |
| C | -2.063799 | 7.701537 | 18.023614 |
| H | -2.460046 | 7.309832 | 18.947414 |
| C | -0.824216 | 7.622074 | 17.482546 |
| H | 0.066325 | 7.123983 | 17.830953 |
| C | -0.257428 | 7.203370 | 9.980346 |
| C | -1.447487 | 6.595179 | 10.407421 |
| H | -2.355385 | 7.185967 | 10.495224 |
| C | -1.497322 | 5.242041 | 10.723805 |
| H | -2.437465 | 4.800996 | 11.049068 |
| C | -0.351535 | 4.446851 | 10.654095 |
| C | 0.839435 | 5.045930 | 10.237172 |
| H | 1.745638 | 4.446901 | 10.174876 |
| C | 0.884423 | 6.395592 | 9.901007 |
| H | 1.828645 | 6.830591 | 9.579632 |
| C | -0.388780 | 3.000143 | 11.063133 |
| H | 0.357328 | 2.411905 | 10.520820 |
| H | -1.374052 | 2.559898 | 10.883252 |
| H | -0.172738 | 2.896098 | 12.134250 |
| C | -1.525542 | 10.748446 | 5.988009 |
| H | -1.363346 | 11.692130 | 5.470809 |
| C | -0.893901 | 10.512563 | 7.201738 |
| H | -0.240380 | 11.279592 | 7.614440 |
| C | 1.597285 | 9.542094 | 9.257805 |
| C | 2.213481 | 9.313962 | 8.019256 |
| H | 1.641075 | 8.873304 | 7.205391 |
| C | 3.547138 | 9.646686 | 7.807075 |
| H | 4.000519 | 9.455668 | 6.836590 |
| C | 4.314653 | 10.227662 | 8.819864 |
| C | 5.744579 | 10.626021 | 8.576830 |
| H | 5.807200 | 11.675011 | 8.261774 |

| | | | |
|---|---|---|---|
| H | 6.199081 | 10.018765 | 7.788728 |
| H | 6.346753 | 10.521122 | 9.484355 |
| C | 3.706262 | 10.455959 | 10.056256 |
| H | 4.282749 | 10.908527 | 10.861393 |
| C | 2.374451 | 10.117931 | 10.266924 |
| H | 1.924617 | 10.299927 | 11.238558 |
| C | -4.606916 | 11.293255 | 12.052345 |
| H | -4.193302 | 12.184730 | 12.530281 |
| H | -4.779144 | 10.548021 | 12.834933 |
| H | -5.579448 | 11.562473 | 11.619366 |
| C | -4.357871 | 9.004611 | 10.113875 |
| H | -5.400168 | 9.224359 | 9.849299 |
| H | -4.373004 | 8.264316 | 10.922097 |
| H | -3.878406 | 8.562545 | 9.235047 |
| C | -3.591785 | 11.833346 | 9.229312 |
| H | -3.089174 | 11.443112 | 8.339319 |
| H | -3.128684 | 12.791797 | 9.488018 |
| H | -4.640309 | 12.029020 | 8.970820 |
| C | 0.277258 | 13.240088 | 10.099482 |
| H | -0.530736 | 13.314484 | 9.363162 |
| H | 1.093219 | 12.661846 | 9.652084 |
| H | 0.647994 | 14.253860 | 10.297047 |
| C | -1.666698 | 13.530367 | 12.459698 |
| H | -2.050127 | 13.092775 | 13.388492 |
| H | -2.510361 | 13.686491 | 11.780468 |
| H | -1.241667 | 14.513091 | 12.700229 |
| C | 1.083553 | 12.391767 | 12.960701 |
| H | 1.245831 | 13.402754 | 13.356107 |
| H | 2.029095 | 12.046638 | 12.532471 |
| H | 0.837986 | 11.732380 | 13.799793 |
| C | 0.145014 | 7.893944 | 14.025073 |
| C | -0.067811 | 6.354913 | 13.929571 |
| H | 0.005555 | 6.136940 | 12.855200 |
| C | 1.077268 | 5.627917 | 14.641304 |
| H | 1.036438 | 5.801951 | 15.723416 |
| H | 2.049743 | 5.979586 | 14.281150 |
| H | 1.013710 | 4.546982 | 14.472584 |
| C | -1.424392 | 5.837249 | 14.400069 |
| H | -1.562074 | 4.801206 | 14.069209 |
| H | -2.240155 | 6.434787 | 13.970668 |
| H | -1.515570 | 5.849889 | 15.491964 |
| C | 1.272811 | 8.338390 | 13.174954 |
| H | 1.285524 | 7.968990 | 12.152540 |
| C | 2.320394 | 9.079832 | 13.647490 |
| H | 3.138278 | 9.317770 | 12.970985 |
| C | 2.365326 | 9.548646 | 14.968620 |
| H | 3.186773 | 10.178506 | 15.287348 |
| C | 1.295990 | 9.292552 | 15.832159 |
| C | 1.212364 | 10.032941 | 17.161270 |
| H | 0.617878 | 9.443249 | 17.866643 |
| C | 0.468538 | 11.362858 | 16.949397 |
| H | 1.047813 | 12.021094 | 16.292015 |
| H | 0.320049 | 11.876728 | 17.906927 |
| H | -0.510294 | 11.204808 | 16.480893 |
| C | 2.572929 | 10.267467 | 17.822275 |
| H | 3.179832 | 10.987779 | 17.264138 |
| H | 3.144076 | 9.337274 | 17.909885 |
| H | 2.429201 | 10.678266 | 18.827380 |
| C | 0.248947 | 8.485904 | 15.387518 |
| C | -4.934205 | 6.663512 | 16.049904 |
| H | -3.847126 | 6.541103 | 16.010187 |
| C | -5.431793 | 6.792717 | 14.603455 |
| H | -6.524119 | 6.874327 | 14.565610 |
| H | -5.139289 | 5.909893 | 14.023775 |
| H | -5.001003 | 7.678888 | 14.126481 |
| C | -5.516408 | 5.417275 | 16.729607 |
| H | -5.156431 | 5.320878 | 17.759436 |
| H | -5.225388 | 4.516732 | 16.178066 |
| H | -6.611008 | 5.451995 | 16.758155 |

Calculated energies and coordinates of **TS[C-D]**

Electronic energy ... -4000.69037875 Eh
Total Enthalpy ... -3999.46667292 Eh
Final Gibbs free energy ... -3999.63700435 Eh

CARTESIAN COORDINATES (ANGSTROEM)

| | | | |
|---|---|---|---|
| Si | -0.125339 | 9.110677 | 9.658202 |
| Si | -0.972391 | 10.512964 | 11.394830 |
| Si | -1.193056 | 9.152979 | 13.289116 |
| P | -3.011686 | 9.403166 | 14.497760 |
| Si | -3.243644 | 11.067330 | 10.872198 |
| Si | 0.204736 | 12.568671 | 11.617747 |
| N | -2.937689 | 8.143101 | 17.008772 |
| N | -0.938420 | 8.452555 | 16.156615 |
| C | -3.293304 | 10.437410 | 4.465102 |
| H | -4.032206 | 11.214889 | 4.695286 |
| H | -3.835131 | 9.573045 | 4.070863 |
| H | -2.645069 | 10.828294 | 3.674681 |
| C | -2.503188 | 10.076843 | 5.692921 |
| C | -2.832686 | 8.960288 | 6.462362 |
| H | -3.653740 | 8.318615 | 6.149414 |
| C | -2.120902 | 8.650152 | 7.617816 |
| H | -2.390606 | 7.758898 | 8.180640 |
| C | -1.058547 | 9.449825 | 8.052939 |
| C | -2.281294 | 8.627940 | 15.900577 |
| C | -4.344306 | 8.303864 | 17.224395 |
| C | -4.789443 | 9.503797 | 17.800124 |
| C | -3.837954 | 10.617958 | 18.194509 |
| H | -2.822252 | 10.324350 | 17.908857 |
| C | -3.845248 | 10.834747 | 19.714164 |
| H | -4.828977 | 11.168668 | 20.062239 |
| H | -3.112981 | 11.601053 | 19.990957 |
| H | -3.595376 | 9.911702 | 20.248031 |
| C | -7.047674 | 8.643886 | 17.640897 |
| H | -8.113252 | 8.777213 | 17.805344 |
| C | -6.579857 | 7.472656 | 17.060816 |
| H | -7.285095 | 6.698723 | 16.771453 |
| C | -5.216102 | 7.276390 | 16.838978 |
| C | -6.161739 | 9.648198 | 18.006730 |
| H | -6.541792 | 10.562574 | 18.453707 |
| C | -4.159127 | 11.918833 | 17.447018 |
| H | -4.142669 | 11.757195 | 16.364745 |
| H | -3.414870 | 12.684714 | 17.691918 |
| H | -5.144858 | 12.307268 | 17.726380 |

| | | | |
|---|---|---|---|
| C | -2.017607 | 7.718831 | 17.952639 |
| H | -2.330365 | 7.310960 | 18.901328 |
| C | -0.783412 | 7.887850 | 17.423367 |
| H | 0.185592 | 7.608419 | 17.803914 |
| C | -0.375142 | 7.301381 | 10.135600 |
| C | -1.592031 | 6.849422 | 10.665827 |
| H | -2.405115 | 7.551617 | 10.834030 |
| C | -1.791715 | 5.509854 | 10.984075 |
| H | -2.749502 | 5.193070 | 11.391400 |
| C | -0.772121 | 4.573219 | 10.811179 |
| C | 0.447657 | 5.016911 | 10.292799 |
| H | 1.258041 | 4.304729 | 10.149966 |
| C | 0.641059 | 6.352920 | 9.957134 |
| H | 1.602732 | 6.663974 | 9.554113 |
| C | -0.961358 | 3.135091 | 11.208579 |
| H | -0.494468 | 2.457317 | 10.486886 |
| H | -2.021682 | 2.878836 | 11.286587 |
| H | -0.499604 | 2.938520 | 12.184515 |
| C | -1.426141 | 10.866012 | 6.107946 |
| H | -1.139805 | 11.733938 | 5.517209 |
| C | -0.722338 | 10.561514 | 7.265783 |
| H | 0.109949 | 11.198270 | 7.559362 |
| C | 1.699137 | 9.392245 | 9.275228 |
| C | 2.222741 | 9.059506 | 8.017490 |
| H | 1.562391 | 8.675219 | 7.242644 |
| C | 3.575901 | 9.215739 | 7.737296 |
| H | 3.955491 | 8.946537 | 6.753738 |
| C | 4.457730 | 9.716369 | 8.698692 |
| C | 5.913199 | 9.922760 | 8.380484 |
| H | 6.086050 | 10.934232 | 7.992259 |
| H | 6.258939 | 9.216505 | 7.620051 |
| H | 6.536598 | 9.803506 | 9.271586 |
| C | 3.942180 | 10.048130 | 9.953478 |
| H | 4.608886 | 10.440435 | 10.719502 |
| C | 2.589639 | 9.887698 | 10.232145 |
| H | 2.212085 | 10.149141 | 11.216354 |
| C | -4.062068 | 12.113772 | 12.218433 |
| H | -3.711152 | 13.148836 | 12.197902 |
| H | -3.898384 | 11.718661 | 13.225143 |
| H | -5.144080 | 12.123023 | 12.033975 |
| C | -4.314182 | 9.527742 | 10.638548 |
| H | -5.345919 | 9.846315 | 10.441934 |
| H | -4.327480 | 8.898268 | 11.535552 |
| H | -3.987301 | 8.926025 | 9.784586 |
| C | -3.315121 | 12.071849 | 9.274324 |
| H | -3.003788 | 11.484348 | 8.406102 |
| H | -2.683879 | 12.966136 | 9.322231 |
| H | -4.348728 | 12.403847 | 9.111573 |
| C | 0.776132 | 13.153422 | 9.914413 |
| H | -0.064266 | 13.227766 | 9.215401 |
| H | 1.514955 | 12.466355 | 9.487652 |
| H | 1.239013 | 14.145402 | 9.991767 |
| C | -0.959355 | 13.875406 | 12.328321 |
| H | -1.428097 | 13.540926 | 13.259903 |
| H | -1.753776 | 14.133321 | 11.621248 |
| H | -0.391359 | 14.789595 | 12.541631 |
| C | 1.689509 | 12.478260 | 12.780194 |
| H | 1.981569 | 13.498343 | 13.060936 |
| H | 2.557624 | 12.000839 | 12.316604 |
| H | 1.453847 | 11.923674 | 13.693899 |
| C | 0.180151 | 7.992561 | 13.965646 |
| C | 0.040691 | 6.449756 | 13.994335 |
| H | 0.196350 | 6.147607 | 12.948626 |
| C | 1.158947 | 5.832622 | 14.842155 |
| H | 1.038231 | 6.103495 | 15.897991 |
| H | 2.144979 | 6.178526 | 14.516621 |
| H | 1.135518 | 4.739000 | 14.775216 |
| C | -1.321368 | 5.905024 | 14.421005 |
| H | -1.402308 | 4.848024 | 14.140241 |
| H | -2.139268 | 6.447685 | 13.935615 |
| H | -1.454522 | 5.964335 | 15.506906 |
| C | 1.412253 | 8.379508 | 13.207408 |
| H | 1.520892 | 7.928183 | 12.223202 |
| C | 2.411243 | 9.127234 | 13.729702 |
| H | 3.308100 | 9.291903 | 13.135644 |
| C | 2.317899 | 9.742632 | 15.012766 |
| H | 3.114800 | 10.399899 | 15.337363 |
| C | 1.166876 | 9.613424 | 15.753238 |
| C | 0.923266 | 10.482441 | 16.981411 |
| H | 0.477289 | 9.871991 | 17.775597 |
| C | -0.103026 | 11.576097 | 16.632645 |
| H | 0.311361 | 12.255645 | 15.878730 |
| H | -0.349046 | 12.165011 | 17.524283 |
| H | -1.028302 | 11.155613 | 16.224720 |
| C | 2.190295 | 11.108506 | 17.563888 |
| H | 2.623486 | 11.849607 | 16.883123 |
| H | 2.953729 | 10.354219 | 17.781466 |
| H | 1.947617 | 11.627080 | 18.497243 |
| C | 0.111597 | 8.790504 | 15.254078 |
| C | -4.723641 | 6.002318 | 16.183244 |
| H | -3.629550 | 6.023266 | 16.183004 |
| C | -5.179010 | 5.934321 | 14.718578 |
| H | -6.271680 | 5.884453 | 14.647091 |
| H | -4.766049 | 5.041189 | 14.235786 |
| H | -4.836103 | 6.817346 | 14.169453 |
| C | -5.163614 | 4.756650 | 16.962866 |
| H | -4.844225 | 4.809575 | 18.008986 |
| H | -4.725709 | 3.858204 | 16.514778 |
| H | -6.252461 | 4.636854 | 16.949477 |

Calculated energies and coordinates of **D**

Electronic energy ... -4000.69880301 Eh
Total Enthalpy ... -3999.47389881 Eh
Final Gibbs free energy ... -3999.64478117 Eh

CARTESIAN COORDINATES (ANGSTROEM)

| | | | |
|---|---|---|---|
| Si | -0.129304 | 9.155241 | 9.740220 |
| Si | -0.803528 | 10.600407 | 11.523491 |
| Si | -1.059134 | 9.220999 | 13.411420 |
| P | -3.112146 | 9.023470 | 14.259090 |
| Si | -3.032092 | 11.350293 | 11.075659 |
| Si | 0.506748 | 12.586666 | 11.647634 |
| N | -2.958488 | 8.048825 | 16.915257 |
| N | -1.009378 | 8.521048 | 16.041874 |

| | | | |
|---|---|---|---|
| C | -3.393588 | 10.621220 | 4.639736 |
| H | -4.108090 | 11.415785 | 4.888134 |
| H | -3.964773 | 9.773269 | 4.251395 |
| H | -2.750913 | 11.000189 | 3.838974 |
| C | -2.588739 | 10.232972 | 5.849580 |
| C | -2.935024 | 9.124320 | 6.623059 |
| H | -3.782860 | 8.509939 | 6.327341 |
| C | -2.206255 | 8.786761 | 7.760283 |
| H | -2.490635 | 7.901887 | 8.325889 |
| C | -1.108867 | 9.550421 | 8.173748 |
| C | -2.358512 | 8.529622 | 15.777704 |
| C | -4.373451 | 8.084563 | 17.138752 |
| C | -4.936709 | 9.283878 | 17.602178 |
| C | -4.103368 | 10.522380 | 17.873335 |
| H | -3.060652 | 10.300360 | 17.622357 |
| C | -4.148251 | 10.902645 | 19.359732 |
| H | -5.162937 | 11.176021 | 19.669717 |
| H | -3.496550 | 11.762345 | 19.550391 |
| H | -3.816087 | 10.072251 | 19.991887 |
| C | -7.096318 | 8.188309 | 17.562687 |
| H | -8.169210 | 8.229031 | 17.728761 |
| C | -6.513501 | 7.017884 | 17.096785 |
| H | -7.136685 | 6.150741 | 16.897102 |
| C | -5.137851 | 6.939853 | 16.873770 |
| C | -6.315667 | 9.308871 | 17.812934 |
| H | -6.784801 | 10.220428 | 18.172532 |
| C | -4.543397 | 11.693211 | 16.984615 |
| H | -4.503622 | 11.410286 | 15.928177 |
| H | -3.879277 | 12.551361 | 17.136585 |
| H | -5.564654 | 12.012438 | 17.221071 |
| C | -1.992751 | 7.763693 | 17.873658 |
| H | -2.260379 | 7.382760 | 18.846988 |
| C | -0.784742 | 8.036813 | 17.326311 |
| H | 0.209627 | 7.904628 | 17.721911 |
| C | -0.473256 | 7.365268 | 10.227281 |
| C | -1.698303 | 6.989626 | 10.794001 |
| H | -2.460624 | 7.740503 | 10.991652 |
| C | -1.969433 | 5.665267 | 11.122362 |
| H | -2.929619 | 5.409471 | 11.565221 |
| C | -1.017389 | 4.666917 | 10.915253 |
| C | 0.210908 | 5.034282 | 10.357273 |
| H | 0.970825 | 4.273040 | 10.190348 |
| C | 0.476957 | 6.356081 | 10.016931 |
| H | 1.442914 | 6.609048 | 9.584517 |
| C | -1.286942 | 3.238326 | 11.301723 |
| H | -1.145746 | 2.563705 | 10.449903 |
| H | -2.308287 | 3.112061 | 11.671051 |
| H | -0.599630 | 2.910543 | 12.090811 |
| C | -1.477648 | 10.985819 | 6.241689 |
| H | -1.177045 | 11.845985 | 5.646642 |
| C | -0.756592 | 10.653579 | 7.381248 |
| H | 0.104532 | 11.260035 | 7.653803 |
| C | 1.691683 | 9.319350 | 9.275784 |
| C | 2.131149 | 8.998384 | 7.983064 |
| H | 1.409445 | 8.692698 | 7.228375 |
| C | 3.478009 | 9.065952 | 7.643160 |
| H | 3.790620 | 8.808351 | 6.633211 |
| C | 4.438453 | 9.461703 | 8.577904 |

| | | | |
|---|---|---|---|
| C | 5.888982 | 9.573356 | 8.195892 |
| H | 6.111166 | 10.573511 | 7.803496 |
| H | 6.152763 | 8.850892 | 7.417810 |
| H | 6.541978 | 9.406914 | 9.057667 |
| C | 4.007426 | 9.778264 | 9.867984 |
| H | 4.736349 | 10.086900 | 10.615354 |
| C | 2.660608 | 9.707630 | 10.206017 |
| H | 2.351287 | 9.951253 | 11.218157 |
| C | -3.732573 | 12.325821 | 12.534942 |
| H | -3.266994 | 13.309431 | 12.638661 |
| H | -3.608154 | 11.781390 | 13.476472 |
| H | -4.807789 | 12.476619 | 12.373428 |
| C | -4.212934 | 9.919436 | 10.722499 |
| H | -5.211306 | 10.331870 | 10.527219 |
| H | -4.301153 | 9.230755 | 11.569442 |
| H | -3.908442 | 9.356608 | 9.834505 |
| C | -3.048772 | 12.473240 | 9.555898 |
| H | -2.766475 | 11.927468 | 8.650243 |
| H | -2.375510 | 13.330486 | 9.660296 |
| H | -4.064592 | 12.864568 | 9.414963 |
| C | 0.983536 | 13.083527 | 9.888352 |
| H | 0.102600 | 13.183304 | 9.245280 |
| H | 1.655811 | 12.345781 | 9.436832 |
| H | 1.501609 | 14.050787 | 9.903710 |
| C | -0.521111 | 13.987045 | 12.393966 |
| H | -0.926043 | 13.719305 | 13.375528 |
| H | -1.358355 | 14.271049 | 11.749425 |
| H | 0.117429 | 14.870324 | 12.521643 |
| C | 2.073173 | 12.490463 | 12.693864 |
| H | 2.529671 | 13.488363 | 12.724189 |
| H | 2.815666 | 11.793810 | 12.296030 |
| H | 1.844213 | 12.185172 | 13.719161 |
| C | 0.259224 | 7.939014 | 13.924058 |
| C | 0.136883 | 6.408647 | 14.079378 |
| H | 0.335823 | 6.033459 | 13.063908 |
| C | 1.234769 | 5.867577 | 15.007249 |
| H | 1.072580 | 6.211764 | 16.035539 |
| H | 2.229452 | 6.199020 | 14.693663 |
| H | 1.222782 | 4.771418 | 15.019756 |
| C | -1.219837 | 5.843451 | 14.497645 |
| H | -1.244918 | 4.767880 | 14.284132 |
| H | -2.044563 | 6.318484 | 13.961400 |
| H | -1.384874 | 5.962120 | 15.573952 |
| C | 1.546477 | 8.267550 | 13.269877 |
| H | 1.780469 | 7.687461 | 12.377675 |
| C | 2.450107 | 9.135856 | 13.763859 |
| H | 3.395647 | 9.273803 | 13.244314 |
| C | 2.218605 | 9.888334 | 14.975927 |
| H | 2.997977 | 10.562301 | 15.313814 |
| C | 1.033656 | 9.831449 | 15.623621 |
| C | 0.672134 | 10.775184 | 16.756547 |
| H | 0.250388 | 10.198480 | 17.590834 |
| C | -0.432400 | 11.739731 | 16.282528 |
| H | -0.055353 | 12.366932 | 15.465879 |
| H | -0.741273 | 12.396571 | 17.103843 |
| H | -1.315289 | 11.209161 | 15.911081 |
| C | 1.857937 | 11.562606 | 17.312112 |
| H | 2.264320 | 12.255240 | 16.566036 |

| | | | |
|---|---|---|---|
| H | 2.665821 | 10.901698 | 17.643263 |
| H | 1.533985 | 12.159803 | 18.170610 |
| C | -0.034957 | 8.970608 | 15.071342 |
| C | -4.521739 | 5.662485 | 16.341009 |
| H | -3.435988 | 5.793647 | 16.323933 |
| C | -4.973601 | 5.400806 | 14.896984 |
| H | -6.054068 | 5.221838 | 14.849209 |
| H | -4.464547 | 4.516199 | 14.497325 |
| H | -4.736151 | 6.257362 | 14.257852 |
| C | -4.829364 | 4.462696 | 17.246102 |
| H | -4.513426 | 4.651158 | 18.277586 |
| H | -4.303453 | 3.573204 | 16.882681 |
| H | -5.900216 | 4.232123 | 17.259312 |

Calculated energies and coordinates of **TS[D-E]**

Electronic energy ... -4000.67943915 Eh
Total Enthalpy ... -3999.45618353 Eh
Final Gibbs free energy ... -3999.62551697 Eh

CARTESIAN COORDINATES (ANGSTROEM)

| | | | |
|---|---|---|---|
| Si | 1.335952 | 0.004819 | -3.252009 |
| Si | 0.086531 | 1.036736 | -1.496693 |
| Si | -0.134908 | -0.276170 | 0.459576 |
| P | -2.115206 | -0.058546 | 1.485516 |
| Si | -2.192027 | 1.285207 | -2.212712 |
| Si | 0.699242 | 3.219401 | -0.785077 |
| N | -1.768312 | -0.473399 | 4.261882 |
| N | 0.114484 | -0.444594 | 3.143202 |
| C | -1.465573 | 1.401760 | -8.634627 |
| H | -1.740479 | 0.509684 | -9.204493 |
| H | -0.837240 | 2.035808 | -9.267870 |
| H | -2.387318 | 1.959325 | -8.426806 |
| C | -0.764151 | 1.040767 | -7.354042 |
| C | -0.924050 | -0.217944 | -6.772772 |
| H | -1.541026 | -0.962320 | -7.271826 |
| C | -0.300745 | -0.536478 | -5.568873 |
| H | -0.433639 | -1.531888 | -5.150066 |
| C | 0.501177 | 0.394488 | -4.899598 |
| C | -1.252942 | -0.313064 | 3.001692 |
| C | -3.147367 | -0.287588 | 4.594227 |
| C | -3.605791 | 1.017920 | 4.826501 |
| C | -2.699734 | 2.229374 | 4.714280 |
| H | -1.709126 | 1.891419 | 4.391273 |
| C | -2.535106 | 2.920088 | 6.075251 |
| H | -3.490996 | 3.314163 | 6.437895 |
| H | -1.835748 | 3.759157 | 5.992685 |
| H | -2.150823 | 2.224682 | 6.829051 |
| C | -5.791347 | 0.077829 | 5.281408 |
| H | -6.832405 | 0.221347 | 5.556932 |
| C | -5.314471 | -1.200949 | 5.027452 |
| H | -5.988808 | -2.049568 | 5.100432 |
| C | -3.982121 | -1.410822 | 4.669017 |
| C | -4.947541 | 1.175428 | 5.175545 |
| H | -5.336931 | 2.171715 | 5.365555 |
| C | -3.211920 | 3.209116 | 3.650008 |
| H | -3.320956 | 2.705026 | 2.684726 |
| H | -2.502654 | 4.035014 | 3.527151 |
| H | -4.180837 | 3.636136 | 3.932207 |
| C | -0.741238 | -0.721045 | 5.166912 |
| H | -0.940473 | -0.883083 | 6.214764 |
| C | 0.420260 | -0.724883 | 4.474895 |
| H | 1.423074 | -0.932578 | 4.806240 |
| C | 1.327442 | -1.868239 | -3.032483 |
| C | 0.185863 | -2.553349 | -2.597440 |
| H | -0.717128 | -1.998000 | -2.350892 |
| C | 0.186073 | -3.934228 | -2.441270 |
| H | -0.713593 | -4.436896 | -2.091625 |
| C | 1.334510 | -4.685457 | -2.704029 |
| C | 2.477221 | -4.008767 | -3.136270 |
| H | 3.386663 | -4.571097 | -3.338382 |
| C | 2.472677 | -2.627105 | -3.302265 |
| H | 3.384094 | -2.129074 | -3.625967 |
| C | 1.330668 | -6.180817 | -2.540601 |
| H | 0.964449 | -6.671318 | -3.451000 |
| H | 0.676808 | -6.488797 | -1.718710 |
| H | 2.336484 | -6.562867 | -2.343077 |
| C | 0.050282 | 1.969685 | -6.698654 |
| H | 0.197871 | 2.954302 | -7.137952 |
| C | 0.665612 | 1.653641 | -5.495092 |
| H | 1.296763 | 2.398515 | -5.012687 |
| C | 3.120547 | 0.587199 | -3.433634 |
| C | 3.746370 | 0.549511 | -4.689181 |
| H | 3.179590 | 0.242046 | -5.565457 |
| C | 5.084221 | 0.898264 | -4.838788 |
| H | 5.543590 | 0.854739 | -5.824258 |
| C | 5.848579 | 1.305815 | -3.742468 |
| C | 7.284264 | 1.721610 | -3.911763 |
| H | 7.752412 | 1.204147 | -4.754338 |
| H | 7.868270 | 1.513086 | -3.010413 |
| H | 7.356177 | 2.798854 | -4.107155 |
| C | 5.231746 | 1.343543 | -2.490362 |
| H | 5.804289 | 1.657438 | -1.619364 |
| C | 3.895184 | 0.990439 | -2.342001 |
| H | 3.446249 | 1.019868 | -1.354815 |
| C | -3.168069 | 2.398747 | -1.035883 |
| H | -2.821204 | 3.436174 | -1.085942 |
| H | -3.119653 | 2.066584 | 0.004795 |
| H | -4.220234 | 2.387381 | -1.348978 |
| C | -3.056824 | -0.394114 | -2.303747 |
| H | -4.123476 | -0.242663 | -2.511973 |
| H | -2.978673 | -0.956295 | -1.366302 |
| H | -2.646598 | -1.005508 | -3.115477 |
| C | -2.344641 | 2.092316 | -3.912947 |
| H | -2.058371 | 1.414586 | -4.720889 |
| H | -1.737527 | 2.997675 | -4.006022 |
| H | -3.395352 | 2.376040 | -4.058086 |
| C | 0.093397 | 4.497369 | -2.041067 |
| H | -0.994120 | 4.486120 | -2.158751 |
| H | 0.539216 | 4.323866 | -3.026939 |
| H | 0.386509 | 5.503350 | -1.715101 |
| C | -0.148717 | 3.524422 | 0.877009 |
| H | 0.118274 | 2.752560 | 1.607777 |
| H | -1.238610 | 3.511725 | 0.785961 |
| H | 0.152701 | 4.500640 | 1.278544 |

| | | | |
|---|---|---|---|
| C | 2.548805 | 3.519985 | -0.569758 |
| H | 2.693779 | 4.503111 | -0.102805 |
| H | 3.071136 | 3.518674 | -1.531400 |
| H | 3.015717 | 2.768566 | 0.072931 |
| C | 0.833393 | -1.868626 | 0.735086 |
| C | 0.285598 | -3.242073 | 1.144200 |
| H | 0.777476 | -3.927796 | 0.435632 |
| C | 0.754736 | -3.655049 | 2.546509 |
| H | 0.221786 | -3.094864 | 3.321759 |
| H | 1.828217 | -3.474916 | 2.668323 |
| H | 0.559711 | -4.720469 | 2.715736 |
| C | -1.217781 | -3.453049 | 0.984986 |
| H | -1.455825 | -4.518129 | 1.096150 |
| H | -1.563285 | -3.125681 | -0.001394 |
| H | -1.790677 | -2.892586 | 1.726668 |
| C | 2.132626 | -1.928230 | 0.137285 |
| H | 2.332057 | -2.839271 | -0.428569 |
| C | 3.154141 | -1.023615 | 0.225054 |
| H | 4.036645 | -1.206851 | -0.383067 |
| C | 3.243003 | -0.008761 | 1.211784 |
| H | 4.199987 | 0.500787 | 1.303819 |
| C | 2.289895 | 0.224626 | 2.168698 |
| C | 2.659612 | 1.075476 | 3.386255 |
| H | 1.765874 | 1.209913 | 4.005073 |
| C | 3.111568 | 2.485920 | 2.981347 |
| H | 4.027808 | 2.459316 | 2.382224 |
| H | 3.319409 | 3.083090 | 3.876933 |
| H | 2.341768 | 2.997724 | 2.398345 |
| C | 3.749208 | 0.411262 | 4.242707 |
| H | 4.695990 | 0.379550 | 3.692935 |
| H | 3.501606 | -0.623752 | 4.500988 |
| H | 3.914706 | 0.971597 | 5.170542 |
| C | 0.959417 | -0.303557 | 2.002627 |
| C | -3.481987 | -2.809201 | 4.367618 |
| H | -2.440409 | -2.733675 | 4.036019 |
| C | -4.281967 | -3.448222 | 3.224883 |
| H | -5.325244 | -3.617300 | 3.513581 |
| H | -3.847302 | -4.417300 | 2.956982 |
| H | -4.269626 | -2.807520 | 2.337510 |
| C | -3.507184 | -3.689512 | 5.624618 |
| H | -2.916428 | -3.245109 | 6.432648 |
| H | -3.096175 | -4.680584 | 5.403717 |
| H | -4.530765 | -3.823053 | 5.992429 |

Calculated energies and coordinates of **E**

Electronic energy ... -4000.70639384 Eh
Total Enthalpy ... -3999.48138666 Eh
Final Gibbs free energy ... -3999.65187977 Eh

CARTESIAN COORDINATES (ANGSTROEM)

| | | | |
|---|---|---|---|
| Si | 1.351564 | 0.010957 | -3.135504 |
| Si | -0.050286 | 0.673113 | -1.305419 |
| Si | -0.090377 | -0.747888 | 0.631417 |
| P | -2.059303 | -0.556104 | 1.698473 |
| Si | -2.319637 | 0.811991 | -2.094091 |
| Si | 0.286263 | 2.883567 | -0.476876 |
| N | -1.650544 | 0.269012 | 4.338714 |
| N | 0.241917 | 0.098641 | 3.213386 |
| C | -1.172056 | 2.009865 | -8.467454 |
| H | -1.297607 | 1.206168 | -9.198535 |
| H | -0.571548 | 2.802068 | -8.925325 |
| H | -2.164773 | 2.429572 | -8.262596 |
| C | -0.537522 | 1.505301 | -7.200577 |
| C | -0.598715 | 0.156418 | -6.848966 |
| H | -1.085494 | -0.548853 | -7.519458 |
| C | -0.041786 | -0.300944 | -5.657231 |
| H | -0.095937 | -1.361377 | -5.421043 |
| C | 0.593754 | 0.575295 | -4.771055 |
| C | -1.146517 | -0.035769 | 3.099945 |
| C | -3.041401 | 0.245580 | 4.673260 |
| C | -3.781074 | 1.428985 | 4.539372 |
| C | -3.158505 | 2.709136 | 4.019536 |
| H | -2.175529 | 2.466639 | 3.601585 |
| C | -2.948386 | 3.712761 | 5.162515 |
| H | -3.906825 | 4.003853 | 5.607670 |
| H | -2.456800 | 4.618062 | 4.789619 |
| H | -2.325414 | 3.286789 | 5.956199 |
| C | -5.699059 | 0.227667 | 5.399802 |
| H | -6.745002 | 0.222132 | 5.693517 |
| C | -4.949739 | -0.937139 | 5.493884 |
| H | -5.418173 | -1.847893 | 5.856066 |
| C | -3.603721 | -0.955889 | 5.126145 |
| C | -5.122291 | 1.396858 | 4.922221 |
| H | -5.723330 | 2.297778 | 4.840672 |
| C | -3.988465 | 3.328095 | 2.888857 |
| H | -4.164245 | 2.601510 | 2.089658 |
| H | -3.456402 | 4.184392 | 2.461155 |
| H | -4.959483 | 3.686818 | 3.247353 |
| C | -0.616285 | 0.539757 | 5.220272 |
| H | -0.804763 | 0.780478 | 6.255025 |
| C | 0.547000 | 0.428884 | 4.545915 |
| H | 1.545559 | 0.537986 | 4.921058 |
| C | 1.493897 | -1.870071 | -3.193420 |
| C | 0.358143 | -2.682113 | -3.084525 |
| H | -0.617629 | -2.221040 | -2.944466 |
| C | 0.448068 | -4.067268 | -3.123371 |
| H | -0.453361 | -4.670049 | -3.030436 |
| C | 1.686674 | -4.700315 | -3.255984 |
| C | 2.824516 | -3.898965 | -3.360417 |
| H | 3.801112 | -4.368663 | -3.460138 |
| C | 2.729456 | -2.509995 | -3.337354 |
| H | 3.636769 | -1.915340 | -3.419252 |
| C | 1.781477 | -6.200847 | -3.302352 |
| H | 1.539456 | -6.576548 | -4.304219 |
| H | 1.078311 | -6.664815 | -2.603183 |
| H | 2.789913 | -6.544977 | -3.055687 |
| C | 0.116366 | 2.382457 | -6.329657 |
| H | 0.191136 | 3.436644 | -6.589396 |
| C | 0.668166 | 1.925880 | -5.140870 |
| H | 1.182077 | 2.633285 | -4.491708 |
| C | 3.098703 | 0.727746 | -3.151259 |
| C | 3.812184 | 0.767706 | -4.359433 |
| H | 3.330953 | 0.444279 | -5.280396 |
| C | 5.127661 | 1.214948 | -4.405661 |

| | | | |
|---|---|---|---|
| H | 5.656731 | 1.229781 | -5.356436 |
| C | 5.781900 | 1.649696 | -3.249496 |
| C | 7.192119 | 2.169862 | -3.308713 |
| H | 7.760691 | 1.689584 | -4.110621 |
| H | 7.718791 | 2.003306 | -2.364340 |
| H | 7.200062 | 3.249659 | -3.502790 |
| C | 5.076222 | 1.616630 | -2.045658 |
| H | 5.559915 | 1.953687 | -1.130037 |
| C | 3.761784 | 1.163851 | -2.002728 |
| H | 3.237807 | 1.143132 | -1.054705 |
| C | -3.415049 | 1.778559 | -0.892593 |
| H | -3.092446 | 2.816376 | -0.764985 |
| H | -3.449228 | 1.304025 | 0.092513 |
| H | -4.433133 | 1.795247 | -1.303360 |
| C | -3.122741 | -0.887780 | -2.293769 |
| H | -4.183861 | -0.751636 | -2.538254 |
| H | -3.066677 | -1.478644 | -1.374593 |
| H | -2.671845 | -1.465958 | -3.107347 |
| C | -2.473126 | 1.695592 | -3.756593 |
| H | -2.096362 | 1.091171 | -4.585690 |
| H | -1.945161 | 2.652920 | -3.779980 |
| H | -3.538689 | 1.893696 | -3.932697 |
| C | -0.390439 | 4.163093 | -1.695495 |
| H | -1.468692 | 4.066944 | -1.851325 |
| H | 0.096130 | 4.082828 | -2.673609 |
| H | -0.195258 | 5.170898 | -1.307301 |
| C | -0.623454 | 3.054419 | 1.172110 |
| H | -0.063683 | 2.539079 | 1.961225 |
| H | -1.630764 | 2.631968 | 1.152068 |
| H | -0.690960 | 4.115678 | 1.445610 |
| C | 2.068382 | 3.385721 | -0.110808 |
| H | 2.054584 | 4.352430 | 0.409335 |
| H | 2.679113 | 3.489889 | -1.012109 |
| H | 2.547613 | 2.656442 | 0.549815 |
| C | 0.520341 | -2.511750 | 0.367042 |
| C | -0.304905 | -3.772021 | 0.544859 |
| H | 0.262866 | -4.586460 | 0.067221 |
| C | -0.428738 | -4.103930 | 2.042253 |
| H | -0.974816 | -3.305081 | 2.556617 |
| H | 0.558911 | -4.201507 | 2.504216 |
| H | -0.978740 | -5.042447 | 2.182998 |
| C | -1.690131 | -3.724036 | -0.102588 |
| H | -2.174961 | -4.704513 | -0.030724 |
| H | -1.626701 | -3.453599 | -1.159621 |
| H | -2.335584 | -2.999797 | 0.405911 |
| C | 1.836170 | -2.635813 | 0.066370 |
| H | 2.188375 | -3.628583 | -0.234474 |
| C | 2.863375 | -1.634003 | 0.026053 |
| H | 3.662815 | -1.848762 | -0.682958 |
| C | 3.098751 | -0.555694 | 0.832415 |
| H | 4.059098 | -0.082336 | 0.647646 |
| C | 2.386689 | -0.041906 | 1.973928 |
| C | 3.263421 | 0.574162 | 3.065332 |
| H | 2.658035 | 1.312767 | 3.601718 |
| C | 4.487651 | 1.350090 | 2.557966 |
| H | 5.261614 | 0.687869 | 2.157440 |
| H | 4.933470 | 1.896818 | 3.395850 |
| H | 4.223668 | 2.080707 | 1.787200 |
| C | 3.731309 | -0.524402 | 4.036628 |
| H | 4.404852 | -1.209530 | 3.510044 |
| H | 2.898911 | -1.124289 | 4.417654 |
| H | 4.276548 | -0.097559 | 4.887343 |
| C | 1.025175 | -0.173280 | 2.070070 |
| C | -2.799031 | -2.236652 | 5.230648 |
| H | -1.807881 | -2.054418 | 4.800710 |
| C | -3.443394 | -3.368641 | 4.420458 |
| H | -4.423201 | -3.644763 | 4.825224 |
| H | -2.806837 | -4.259635 | 4.448417 |
| H | -3.569592 | -3.069114 | 3.375264 |
| C | -2.607512 | -2.642391 | 6.698771 |
| H | -2.127529 | -1.842256 | 7.272226 |
| H | -1.981771 | -3.538820 | 6.768563 |
| H | -3.569755 | -2.864591 | 7.173840 |

Calculated energies and coordinates of **TS[A-F]**

Electronic energy ... -4057.21572926 Eh
Total Enthalpy ... -4055.95635514 Eh
Final Gibbs free energy ... -4056.13297456 Eh

CARTESIAN COORDINATES (ANGSTROEM)

| | | | |
|---|---|---|---|
| Si | 0.654597 | -0.172440 | -3.603206 |
| Si | -0.590891 | 0.880913 | -1.879717 |
| Si | 0.508676 | 0.107739 | 0.058516 |
| P | -0.798109 | 0.772468 | 1.753631 |
| Si | -2.814765 | 0.072201 | -1.807151 |
| Si | -0.623636 | 3.245317 | -2.030262 |
| N | -1.232662 | -0.401833 | 4.210328 |
| N | 0.760446 | -0.951167 | 3.534288 |
| N | 2.208003 | 0.971510 | -0.073080 |
| C | -0.024203 | 2.616876 | -9.128704 |
| H | -1.065213 | 2.599344 | -9.464902 |
| H | 0.595227 | 2.147255 | -9.898437 |
| H | 0.279707 | 3.668783 | -9.058598 |
| C | 0.134168 | 1.926921 | -7.801342 |
| C | 1.322419 | 1.271828 | -7.468733 |
| H | 2.133424 | 1.237360 | -8.193451 |
| C | 1.482522 | 0.663951 | -6.228058 |
| H | 2.421200 | 0.163027 | -5.999909 |
| C | 0.459810 | 0.687743 | -5.269688 |
| C | -0.360578 | -0.245271 | 3.146933 |
| C | -2.511970 | 0.240003 | 4.322574 |
| C | -2.583672 | 1.460324 | 5.005771 |
| C | -1.356934 | 2.155779 | 5.565182 |
| H | -0.501100 | 1.480106 | 5.460552 |
| C | -1.509061 | 2.465538 | 7.059883 |
| H | -2.302797 | 3.198305 | 7.240720 |
| H | -0.577054 | 2.885262 | 7.453820 |
| H | -1.748843 | 1.563031 | 7.632031 |
| C | -4.968743 | 1.453606 | 4.595707 |
| H | -5.938310 | 1.932611 | 4.699134 |
| C | -4.866294 | 0.244474 | 3.916901 |
| H | -5.758268 | -0.203217 | 3.492044 |
| C | -3.635403 | -0.390175 | 3.763375 |
| C | -3.841171 | 2.054861 | 5.132220 |

| | | | |
|---|---|---|---|
| H | -3.933463 | 3.005329 | 5.649852 |
| C | -1.039516 | 3.425556 | 4.762266 |
| H | -0.883429 | 3.185295 | 3.705152 |
| H | -0.132061 | 3.901219 | 5.152058 |
| H | -1.858268 | 4.150428 | 4.834461 |
| C | -0.644016 | -1.134226 | 5.229048 |
| H | -1.163836 | -1.338447 | 6.151825 |
| C | 0.591701 | -1.479178 | 4.810122 |
| H | 1.371406 | -2.049877 | 5.288972 |
| C | 0.116398 | -1.973948 | -3.765322 |
| C | -0.297437 | -2.502206 | -4.994386 |
| H | -0.317093 | -1.862459 | -5.874340 |
| C | -0.692747 | -3.831718 | -5.112722 |
| H | -1.016819 | -4.211682 | -6.079481 |
| C | -0.680437 | -4.686266 | -4.009472 |
| C | -0.274039 | -4.163837 | -2.778383 |
| H | -0.277173 | -4.804412 | -1.898063 |
| C | 0.110128 | -2.834209 | -2.655653 |
| H | 0.383159 | -2.447197 | -1.672739 |
| C | -1.074688 | -6.132183 | -4.141522 |
| H | -1.591521 | -6.487164 | -3.244495 |
| H | -0.190266 | -6.766246 | -4.281835 |
| H | -1.731472 | -6.289117 | -5.002021 |
| C | -0.892945 | 1.946673 | -6.855001 |
| H | -1.831098 | 2.444646 | -7.092360 |
| C | -0.729457 | 1.341082 | -5.614315 |
| H | -1.545698 | 1.373854 | -4.897014 |
| C | 2.490800 | -0.127466 | -3.152395 |
| C | 3.217636 | 1.065093 | -3.291754 |
| H | 2.756126 | 1.921198 | -3.778113 |
| C | 4.517362 | 1.184031 | -2.814944 |
| H | 5.051510 | 2.125034 | -2.935317 |
| C | 5.151546 | 0.107402 | -2.185235 |
| C | 6.541290 | 0.254040 | -1.628548 |
| H | 6.979588 | -0.719146 | -1.388904 |
| H | 6.531565 | 0.853001 | -0.708961 |
| H | 7.204221 | 0.761823 | -2.336897 |
| C | 4.452892 | -1.094072 | -2.084323 |
| H | 4.935294 | -1.954099 | -1.624151 |
| C | 3.146962 | -1.210483 | -2.555108 |
| H | 2.635708 | -2.164424 | -2.456980 |
| C | -3.846457 | 1.139543 | -0.636737 |
| H | -3.859884 | 2.191099 | -0.942292 |
| H | -3.448953 | 1.090891 | 0.383649 |
| H | -4.884977 | 0.783552 | -0.624874 |
| C | -2.874543 | -1.714217 | -1.205756 |
| H | -3.918696 | -2.030826 | -1.090225 |
| H | -2.373766 | -1.823239 | -0.236907 |
| H | -2.390553 | -2.386203 | -1.920795 |
| C | -3.628741 | 0.111879 | -3.515917 |
| H | -3.073382 | -0.500697 | -4.235327 |
| H | -3.704431 | 1.128850 | -3.916264 |
| H | -4.646197 | -0.293716 | -3.447054 |
| C | -2.047915 | 3.881442 | -3.104720 |
| H | -3.017476 | 3.481716 | -2.791102 |
| H | -1.897539 | 3.623442 | -4.158289 |
| H | -2.099379 | 4.975539 | -3.034037 |
| C | -0.832396 | 3.949712 | -0.290914 |

| | | | |
|---|---|---|---|
| H | 0.006922 | 3.663744 | 0.352958 |
| H | -1.743389 | 3.576249 | 0.189012 |
| H | -0.885552 | 5.045346 | -0.325898 |
| C | 0.964273 | 3.952834 | -2.776015 |
| H | 0.906255 | 5.048528 | -2.797488 |
| H | 1.100165 | 3.603944 | -3.806041 |
| H | 1.852553 | 3.675712 | -2.198183 |
| C | 2.430173 | -2.038339 | 2.102148 |
| C | 1.622032 | -3.304274 | 1.840910 |
| H | 1.921024 | -3.613044 | 0.829000 |
| C | 2.077902 | -4.408536 | 2.812017 |
| H | 1.799271 | -4.153155 | 3.840890 |
| H | 3.162300 | -4.554110 | 2.780381 |
| H | 1.591752 | -5.357810 | 2.561128 |
| C | 0.093380 | -3.210275 | 1.831021 |
| H | -0.308985 | -4.110294 | 1.352461 |
| H | -0.248797 | -2.340223 | 1.262168 |
| H | -0.326334 | -3.163856 | 2.841334 |
| C | 3.717481 | -1.996762 | 1.555310 |
| H | 4.039431 | -2.819157 | 0.921138 |
| C | 4.576056 | -0.935540 | 1.791916 |
| H | 5.566674 | -0.931093 | 1.345802 |
| C | 4.165018 | 0.128869 | 2.584715 |
| H | 2.820856 | 1.015066 | 0.731623 |
| H | 4.843765 | 0.956177 | 2.772716 |
| C | 2.886165 | 0.147190 | 3.145444 |
| C | 2.463264 | 1.316620 | 4.015743 |
| H | 1.406916 | 1.192422 | 4.270081 |
| C | 2.582514 | 2.654290 | 3.275331 |
| H | 3.618878 | 2.871438 | 2.992432 |
| H | 2.237056 | 3.468935 | 3.920560 |
| H | 1.963090 | 2.652901 | 2.372526 |
| C | 3.261669 | 1.328608 | 5.326767 |
| H | 4.329391 | 1.489062 | 5.139213 |
| H | 3.152790 | 0.380145 | 5.863377 |
| H | 2.908652 | 2.135023 | 5.979101 |
| C | 2.039196 | -0.945688 | 2.888956 |
| C | -3.507596 | -1.716313 | 3.038383 |
| H | -2.537677 | -1.710485 | 2.524521 |
| C | -4.584495 | -1.922392 | 1.971855 |
| H | -5.574040 | -2.077211 | 2.416960 |
| H | -4.349223 | -2.813531 | 1.381915 |
| H | -4.635277 | -1.069681 | 1.288623 |
| C | -3.525533 | -2.889140 | 4.032228 |
| H | -2.717747 | -2.819337 | 4.766751 |
| H | -3.411404 | -3.838753 | 3.497513 |
| H | -4.477527 | -2.914770 | 4.575451 |
| H | 2.014067 | -0.401005 | -0.282435 |
| H | 2.519589 | 1.558219 | -0.840140 |

Calculated energies and coordinates of **F**

Electronic energy ... -4057.31523585 Eh
Total Enthalpy ... -4056.05221421 Eh
Final Gibbs free energy ... -4056.22845968 Eh

CARTESIAN COORDINATES (ANGSTROEM)

| | | | |
|---|---|---|---|
| Si | 0.676335 | -0.150949 | -3.612539 |
| Si | -0.606234 | 0.832928 | -1.877073 |
| Si | 0.594693 | 0.258963 | 0.072967 |
| P | -0.819296 | 0.714875 | 1.752488 |
| Si | -2.799659 | -0.051118 | -1.780210 |
| Si | -0.675484 | 3.197271 | -2.003205 |
| N | -1.225992 | -0.338654 | 4.252760 |
| N | 0.762557 | -0.926776 | 3.588497 |
| N | 2.048794 | 1.229601 | 0.045202 |
| C | 0.025146 | 2.788855 | -9.061779 |
| H | -1.003305 | 2.725066 | -9.429623 |
| H | 0.691488 | 2.378173 | -9.826055 |
| H | 0.268083 | 3.853000 | -8.951020 |
| C | 0.183582 | 2.067282 | -7.751393 |
| C | 1.396873 | 1.470154 | -7.401277 |
| H | 2.229662 | 1.503659 | -8.100912 |
| C | 1.554491 | 0.832318 | -6.175052 |
| H | 2.511975 | 0.374392 | -5.935496 |
| C | 0.504417 | 0.769196 | -5.248901 |
| C | -0.356413 | -0.228632 | 3.179032 |
| C | -2.507036 | 0.303119 | 4.333970 |
| C | -2.578768 | 1.578067 | 4.908932 |
| C | -1.349362 | 2.332703 | 5.375209 |
| H | -0.471642 | 1.705178 | 5.188850 |
| C | -1.403958 | 2.614032 | 6.882694 |
| H | -2.240371 | 3.275537 | 7.133790 |
| H | -0.480713 | 3.103838 | 7.211265 |
| H | -1.525157 | 1.688611 | 7.455956 |
| C | -4.974313 | 1.506802 | 4.571161 |
| H | -5.948164 | 1.978958 | 4.665934 |
| C | -4.870665 | 0.252358 | 3.980518 |
| H | -5.765669 | -0.236884 | 3.610959 |
| C | -3.634233 | -0.375842 | 3.843722 |
| C | -3.841345 | 2.164124 | 5.024313 |
| H | -3.933308 | 3.152534 | 5.465779 |
| C | -1.161899 | 3.626296 | 4.570560 |
| H | -1.095248 | 3.406764 | 3.500174 |
| H | -0.240549 | 4.132321 | 4.880026 |
| H | -1.994993 | 4.319280 | 4.732813 |
| C | -0.632424 | -1.028356 | 5.299000 |
| H | -1.147363 | -1.190706 | 6.232731 |
| C | 0.599339 | -1.397051 | 4.888681 |
| H | 1.379166 | -1.949485 | 5.388395 |
| C | 0.147937 | -1.944067 | -3.880462 |
| C | -0.191160 | -2.418645 | -5.154045 |
| H | -0.160079 | -1.741355 | -6.004856 |
| C | -0.576809 | -3.741012 | -5.353461 |
| H | -0.842082 | -4.077680 | -6.353608 |
| C | -0.630230 | -4.643535 | -4.290137 |
| C | -0.298551 | -4.176378 | -3.015556 |
| H | -0.351154 | -4.855228 | -2.166164 |
| C | 0.076462 | -2.853375 | -2.814266 |
| H | 0.295490 | -2.517173 | -1.801652 |
| C | -1.013756 | -6.081619 | -4.509623 |
| H | -1.556884 | -6.484601 | -3.649263 |
| H | -0.122275 | -6.704435 | -4.655148 |
| H | -1.642234 | -6.195251 | -5.397651 |
| C | -0.871257 | 1.997732 | -6.838178 |
| H | -1.829340 | 2.447843 | -7.090822 |
| C | -0.710333 | 1.363803 | -5.611555 |
| H | -1.550026 | 1.326055 | -4.921466 |
| C | 2.503399 | -0.126519 | -3.117109 |
| C | 3.251309 | 1.057761 | -3.225558 |
| H | 2.802075 | 1.938343 | -3.677795 |
| C | 4.557811 | 1.136593 | -2.759613 |
| H | 5.106409 | 2.071882 | -2.854208 |
| C | 5.176563 | 0.031504 | -2.165755 |
| C | 6.579145 | 0.133360 | -1.631607 |
| H | 6.970979 | -0.848165 | -1.349569 |
| H | 6.613445 | 0.778687 | -0.745035 |
| H | 7.256771 | 0.569725 | -2.373272 |
| C | 4.449962 | -1.155243 | -2.079016 |
| H | 4.916615 | -2.035240 | -1.640935 |
| C | 3.139783 | -1.233563 | -2.543717 |
| H | 2.608615 | -2.178363 | -2.460319 |
| C | -3.888703 | 1.043634 | -0.689902 |
| H | -3.942784 | 2.073391 | -1.058323 |
| H | -3.503597 | 1.070957 | 0.335901 |
| H | -4.911011 | 0.643997 | -0.665515 |
| C | -2.822435 | -1.794608 | -1.059381 |
| H | -3.860035 | -2.135878 | -0.954896 |
| H | -2.357506 | -1.807983 | -0.066899 |
| H | -2.294529 | -2.502924 | -1.704467 |
| C | -3.563933 | -0.129938 | -3.509492 |
| H | -2.951710 | -0.735578 | -4.187814 |
| H | -3.682403 | 0.866134 | -3.950667 |
| H | -4.559336 | -0.588745 | -3.455973 |
| C | -2.069708 | 3.806493 | -3.131611 |
| H | -3.037348 | 3.360136 | -2.880482 |
| H | -1.855093 | 3.587114 | -4.182590 |
| H | -2.169532 | 4.894901 | -3.032533 |
| C | -0.963981 | 3.882746 | -0.269159 |
| H | -0.136635 | 3.605761 | 0.393517 |
| H | -1.885668 | 3.494777 | 0.177399 |
| H | -1.032106 | 4.977681 | -0.302999 |
| C | 0.937900 | 3.912289 | -2.673169 |
| H | 0.890701 | 5.008606 | -2.667064 |
| H | 1.111598 | 3.589188 | -3.705976 |
| H | 1.789497 | 3.604273 | -2.057477 |
| C | 2.367725 | -2.053797 | 2.116358 |
| C | 1.543256 | -3.320864 | 1.916553 |
| H | 1.790115 | -3.653200 | 0.897896 |
| C | 2.049635 | -4.403848 | 2.887181 |
| H | 1.833415 | -4.117830 | 3.923094 |
| H | 3.129509 | -4.556819 | 2.796006 |
| H | 1.544593 | -5.356110 | 2.690723 |
| C | 0.014773 | -3.232464 | 1.990673 |
| H | -0.407946 | -4.145341 | 1.556003 |
| H | -0.377481 | -2.379350 | 1.431394 |
| H | -0.344044 | -3.168950 | 3.023064 |
| C | 3.622462 | -2.022039 | 1.499923 |
| H | 3.902972 | -2.845091 | 0.847092 |
| C | 4.506720 | -0.974180 | 1.701860 |
| H | 5.471194 | -0.976403 | 1.201871 |
| C | 4.159803 | 0.078494 | 2.538500 |
| H | 2.688223 | 1.086754 | 0.817325 |

| | | | |
|---|---|---|---|
| H | 4.861818 | 0.891357 | 2.703209 |
| C | 2.910311 | 0.113280 | 3.158666 |
| C | 2.555582 | 1.271948 | 4.072661 |
| H | 1.545998 | 1.106941 | 4.460037 |
| C | 2.536636 | 2.609257 | 3.320788 |
| H | 3.518736 | 2.842962 | 2.893456 |
| H | 2.271648 | 3.418873 | 4.009664 |
| H | 1.801862 | 2.592446 | 2.510011 |
| C | 3.506089 | 1.322430 | 5.277376 |
| H | 4.533475 | 1.541375 | 4.966165 |
| H | 3.513972 | 0.369699 | 5.817617 |
| H | 3.192357 | 2.109240 | 5.971974 |
| C | 2.025954 | -0.955285 | 2.917761 |
| C | -3.504884 | -1.743710 | 3.201520 |
| H | -2.533035 | -1.768879 | 2.692922 |
| C | -4.576126 | -2.009436 | 2.141740 |
| H | -5.567438 | -2.145265 | 2.588951 |
| H | -4.334497 | -2.928600 | 1.598733 |
| H | -4.627607 | -1.191825 | 1.416525 |
| C | -3.525143 | -2.854531 | 4.263416 |
| H | -2.713431 | -2.739885 | 4.987870 |
| H | -3.415896 | -3.835835 | 3.788018 |
| H | -4.474943 | -2.842865 | 4.810780 |
| H | 0.869909 | -1.213509 | -0.030140 |
| H | 2.579352 | 1.170168 | -0.818291 |

Calculated energies and coordinates of **G**

Electronic energy ... -4113.82690866 Eh
Total Enthalpy ... -4112.51939254 Eh
Final Gibbs free energy ... -4112.70231004 Eh

CARTESIAN COORDINATES (ANGSTROEM)

| | | | |
|---|---|---|---|
| Si | 0.680377 | -0.128171 | -3.636662 |
| Si | -0.370555 | 0.569911 | -1.621291 |
| Si | 0.596399 | -0.591591 | 0.262965 |
| P | -0.641505 | 0.632138 | 1.735473 |
| Si | -2.587490 | -0.297199 | -1.640837 |
| Si | -0.592614 | 2.937316 | -1.737700 |
| N | -1.550516 | -0.053654 | 4.261158 |
| N | 0.444623 | -0.877014 | 3.987348 |
| N | 2.385263 | 0.361931 | 0.313279 |
| C | -0.843233 | 3.793817 | -8.241055 |
| H | -1.431009 | 3.312842 | -9.029149 |
| H | 0.034897 | 4.257141 | -8.700102 |
| H | -1.459032 | 4.597874 | -7.819194 |
| C | -0.449521 | 2.809656 | -7.173751 |
| C | 0.784334 | 2.897805 | -6.528034 |
| H | 1.489147 | 3.671829 | -6.825160 |
| C | 1.130014 | 2.006383 | -5.516624 |
| H | 2.109734 | 2.095173 | -5.052198 |
| C | 0.253502 | 0.996104 | -5.099434 |
| C | -0.543326 | -0.177531 | 3.316264 |
| C | -2.789055 | 0.642584 | 4.055504 |
| C | -2.863783 | 1.991233 | 4.423207 |
| C | -1.668089 | 2.755633 | 4.956098 |
| H | -0.801799 | 2.086121 | 4.940411 |
| C | -1.891738 | 3.194341 | 6.409569 |
| H | -2.733031 | 3.892109 | 6.486421 |
| H | -1.000225 | 3.699786 | 6.797359 |
| H | -2.108949 | 2.336737 | 7.055380 |
| C | -5.189671 | 1.963616 | 3.757275 |
| H | -6.136476 | 2.482650 | 3.637216 |
| C | -5.082804 | 0.628727 | 3.382845 |
| H | -5.947225 | 0.124141 | 2.964814 |
| C | -3.880729 | -0.062618 | 3.521881 |
| C | -4.092186 | 2.637655 | 4.268876 |
| H | -4.183713 | 3.684937 | 4.543127 |
| C | -1.330765 | 3.950984 | 4.056096 |
| H | -1.174179 | 3.618611 | 3.025588 |
| H | -0.416948 | 4.442995 | 4.408504 |
| H | -2.133793 | 4.696364 | 4.061600 |
| C | -1.157521 | -0.587982 | 5.478487 |
| H | -1.799779 | -0.557011 | 6.344601 |
| C | 0.078483 | -1.104918 | 5.310390 |
| H | 0.733113 | -1.615778 | 5.998677 |
| C | 0.232771 | -1.911830 | -4.068205 |
| C | -0.002480 | -2.343008 | -5.379862 |
| H | 0.028200 | -1.625529 | -6.197017 |
| C | -0.268224 | -3.680030 | -5.663877 |
| H | -0.448533 | -3.985190 | -6.692799 |
| C | -0.303412 | -4.637668 | -4.648887 |
| C | -0.053691 | -4.216836 | -3.339657 |
| H | -0.070589 | -4.945278 | -2.530833 |
| C | 0.205859 | -2.881558 | -3.054864 |
| H | 0.388211 | -2.583915 | -2.021031 |
| C | -0.627973 | -6.075904 | -4.948665 |
| H | -1.696774 | -6.272734 | -4.798239 |
| H | -0.076238 | -6.754177 | -4.290347 |
| H | -0.389399 | -6.331229 | -5.985292 |
| C | -1.319787 | 1.788195 | -6.783485 |
| H | -2.282902 | 1.686817 | -7.280162 |
| C | -0.977343 | 0.905619 | -5.766563 |
| H | -1.685882 | 0.131189 | -5.488256 |
| C | 2.575629 | -0.091420 | -3.514856 |
| C | 3.260249 | 0.934604 | -2.852932 |
| H | 2.692231 | 1.720020 | -2.359219 |
| C | 4.650250 | 0.985513 | -2.818207 |
| H | 5.148053 | 1.797605 | -2.291512 |
| C | 5.417536 | 0.004948 | -3.449579 |
| C | 6.920651 | 0.065423 | -3.437984 |
| H | 7.290180 | 0.641544 | -2.584777 |
| H | 7.297393 | 0.546359 | -4.349171 |
| H | 7.359620 | -0.936132 | -3.394019 |
| C | 4.745465 | -1.021850 | -4.118805 |
| H | 5.320916 | -1.798092 | -4.620296 |
| C | 3.354489 | -1.067858 | -4.152950 |
| H | 2.863582 | -1.885091 | -4.677711 |
| C | -3.747459 | 0.825752 | -0.654544 |
| H | -3.809905 | 1.828880 | -1.089900 |
| H | -3.409200 | 0.923919 | 0.383315 |
| H | -4.760636 | 0.402439 | -0.655986 |
| C | -2.629934 | -2.020779 | -0.865083 |
| H | -3.659969 | -2.399252 | -0.863453 |
| H | -2.267319 | -1.998416 | 0.168352 |

| | | | |
|---|---|---|---|
| H | -2.012030 | -2.724603 | -1.431273 |
| C | -3.331088 | -0.476946 | -3.371985 |
| H | -2.765127 | -1.199591 | -3.970818 |
| H | -3.361557 | 0.476296 | -3.909789 |
| H | -4.360432 | -0.847942 | -3.284592 |
| C | -1.762260 | 3.434545 | -3.139671 |
| H | -2.746237 | 2.964607 | -3.038278 |
| H | -1.355029 | 3.149937 | -4.115394 |
| H | -1.907247 | 4.522710 | -3.132836 |
| C | -1.239616 | 3.673084 | -0.121562 |
| H | -0.507349 | 3.535030 | 0.680961 |
| H | -2.170774 | 3.208702 | 0.214644 |
| H | -1.412189 | 4.749319 | -0.254486 |
| C | 1.045909 | 3.838875 | -2.071962 |
| H | 0.856413 | 4.920017 | -2.087128 |
| H | 1.478703 | 3.567683 | -3.040357 |
| H | 1.792553 | 3.655050 | -1.289624 |
| C | 2.143027 | -2.324086 | 2.972188 |
| C | 1.262307 | -3.565391 | 2.885134 |
| H | 1.634994 | -4.106460 | 2.003997 |
| C | 1.527691 | -4.452524 | 4.115473 |
| H | 1.170395 | -3.958849 | 5.026491 |
| H | 2.594203 | -4.665851 | 4.240717 |
| H | 0.990989 | -5.402725 | 4.019105 |
| C | -0.244870 | -3.386742 | 2.679736 |
| H | -0.670021 | -4.348865 | 2.372299 |
| H | -0.452390 | -2.655546 | 1.892635 |
| H | -0.754584 | -3.084178 | 3.599608 |
| C | 3.473517 | -2.469472 | 2.562285 |
| H | 3.781981 | -3.418244 | 2.128779 |
| C | 4.395252 | -1.443207 | 2.696046 |
| H | 5.418418 | -1.584446 | 2.359723 |
| C | 4.011379 | -0.233702 | 3.261797 |
| H | 2.362480 | 1.309070 | -0.051383 |
| H | 4.741647 | 0.562213 | 3.380574 |
| C | 2.696118 | -0.031060 | 3.686471 |
| C | 2.312229 | 1.286009 | 4.337035 |
| H | 1.245498 | 1.252931 | 4.574813 |
| C | 2.521543 | 2.477846 | 3.394047 |
| H | 3.566760 | 2.563539 | 3.073861 |
| H | 2.252689 | 3.409919 | 3.902875 |
| H | 1.876882 | 2.382482 | 2.513220 |
| C | 3.075789 | 1.477016 | 5.655234 |
| H | 4.153556 | 1.572727 | 5.482446 |
| H | 2.917171 | 0.628994 | 6.329735 |
| H | 2.734404 | 2.386750 | 6.160912 |
| C | 1.777748 | -1.084625 | 3.517025 |
| C | -3.750817 | -1.520717 | 3.122424 |
| H | -2.743092 | -1.648128 | 2.705486 |
| C | -4.749141 | -1.939918 | 2.041775 |
| H | -5.772551 | -1.985799 | 2.431945 |
| H | -4.493529 | -2.939408 | 1.676173 |
| H | -4.728200 | -1.254012 | 1.190911 |
| C | -3.894814 | -2.445658 | 4.342188 |
| H | -3.137789 | -2.242877 | 5.104426 |
| H | -3.790077 | -3.492676 | 4.035973 |
| H | -4.883267 | -2.320154 | 4.799579 |
| H | 2.694230 | 0.403048 | 1.283617 |

| | | | |
|---|---|---|---|
| H | 3.074578 | -0.210299 | -0.204068 |
| N | 3.735160 | -2.014575 | -0.847218 |
| H | 3.793966 | -2.009211 | -1.861473 |
| H | 4.461717 | -2.631556 | -0.504003 |
| H | 2.837705 | -2.431500 | -0.602996 |

Calculated energies and coordinates of **TS[G-H]**

Electronic energy ... -4113.80297463 Eh
Total Enthalpy ... -4112.49989350 Eh
Final Gibbs free energy ... -4112.67887209 Eh

CARTESIAN COORDINATES (ANGSTROEM)

| | | | |
|---|---|---|---|
| Si | 0.643129 | -0.012116 | -3.580963 |
| Si | -0.574898 | 0.892829 | -1.764575 |
| Si | 0.683553 | 0.210085 | 0.160341 |
| P | -0.841822 | 0.793705 | 1.733994 |
| Si | -2.727723 | -0.098297 | -1.713775 |
| Si | -0.874138 | 3.228261 | -2.016941 |
| N | -1.453892 | -0.312575 | 4.163198 |
| N | 0.548106 | -0.990486 | 3.615590 |
| N | 2.007137 | 1.556094 | 0.254237 |
| C | -0.283691 | 2.943099 | -8.992967 |
| H | -1.217585 | 2.625870 | -9.467274 |
| H | 0.535452 | 2.775564 | -9.698296 |
| H | -0.355887 | 4.024295 | -8.821283 |
| C | -0.051434 | 2.211573 | -7.699055 |
| C | 1.235975 | 1.863870 | -7.287209 |
| H | 2.083550 | 2.102187 | -7.926832 |
| C | 1.451378 | 1.214289 | -6.074959 |
| H | 2.468340 | 0.953883 | -5.790537 |
| C | 0.387348 | 0.890091 | -5.223031 |
| C | -0.506705 | -0.209954 | 3.149823 |
| C | -2.688597 | 0.415659 | 4.197519 |
| C | -2.683094 | 1.706239 | 4.742492 |
| C | -1.409304 | 2.374880 | 5.220103 |
| H | -0.568605 | 1.721042 | 4.968455 |
| C | -1.419688 | 2.556517 | 6.743882 |
| H | -2.235937 | 3.217366 | 7.056878 |
| H | -0.477259 | 3.001571 | 7.082666 |
| H | -1.549443 | 1.597219 | 7.256628 |
| C | -5.074907 | 1.781770 | 4.383272 |
| H | -6.016829 | 2.318129 | 4.457402 |
| C | -5.048238 | 0.508877 | 3.825683 |
| H | -5.970360 | 0.068896 | 3.460182 |
| C | -3.853258 | -0.201268 | 3.715589 |
| C | -3.904402 | 2.375741 | 4.831883 |
| H | -3.936030 | 3.377548 | 5.251320 |
| C | -1.172443 | 3.707548 | 4.498475 |
| H | -1.182096 | 3.559538 | 3.413976 |
| H | -0.198587 | 4.121488 | 4.785362 |
| H | -1.936853 | 4.448345 | 4.758516 |
| C | -0.995029 | -1.100284 | 5.203829 |
| H | -1.580509 | -1.262187 | 6.094916 |
| C | 0.234084 | -1.534251 | 4.864558 |
| H | 0.942645 | -2.144882 | 5.400716 |
| C | 0.180197 | -1.829595 | -3.804377 |

| | | | |
|---|---|---|---|
| C | -0.295397 | -2.342684 | -5.016392 |
| H | -0.404095 | -1.681039 | -5.873519 |
| C | -0.630359 | -3.688110 | -5.148447 |
| H | -0.999704 | -4.058835 | -6.102554 |
| C | -0.495580 | -4.571196 | -4.076325 |
| C | -0.012011 | -4.066572 | -2.865144 |
| H | 0.097928 | -4.735376 | -2.012962 |
| C | 0.312208 | -2.722460 | -2.728999 |
| H | 0.661112 | -2.351184 | -1.763639 |
| C | -0.888836 | -6.017744 | -4.206116 |
| H | -1.910074 | -6.177356 | -3.838276 |
| H | -0.227343 | -6.664614 | -3.621262 |
| H | -0.859354 | -6.347333 | -5.248724 |
| C | -1.119528 | 1.883340 | -6.859654 |
| H | -2.134383 | 2.139067 | -7.158583 |
| C | -0.901850 | 1.242702 | -5.646058 |
| H | -1.754010 | 1.010341 | -5.012726 |
| C | 2.524761 | 0.013275 | -3.289792 |
| C | 3.190848 | 1.210380 | -2.986545 |
| H | 2.616891 | 2.127936 | -2.877347 |
| C | 4.572023 | 1.263868 | -2.836279 |
| H | 5.051636 | 2.211361 | -2.598526 |
| C | 5.356081 | 0.115181 | -2.989405 |
| C | 6.854131 | 0.178616 | -2.864577 |
| H | 7.161647 | 0.898204 | -2.099503 |
| H | 7.306830 | 0.497372 | -3.811513 |
| H | 7.276971 | -0.798221 | -2.612312 |
| C | 4.704391 | -1.083309 | -3.297250 |
| H | 5.290640 | -1.990881 | -3.430280 |
| C | 3.317739 | -1.131188 | -3.448007 |
| H | 2.844626 | -2.079675 | -3.691705 |
| C | -3.895394 | 0.994057 | -0.701227 |
| H | -3.988042 | 1.999514 | -1.125032 |
| H | -3.536635 | 1.092011 | 0.329911 |
| H | -4.899432 | 0.550111 | -0.682876 |
| C | -2.667661 | -1.804320 | -0.908115 |
| H | -3.677092 | -2.232355 | -0.868787 |
| H | -2.281502 | -1.729501 | 0.114547 |
| H | -2.028247 | -2.489656 | -1.472934 |
| C | -3.511074 | -0.322013 | -3.424606 |
| H | -2.885748 | -0.938755 | -4.079982 |
| H | -3.697720 | 0.637142 | -3.920354 |
| H | -4.478517 | -0.828666 | -3.313498 |
| C | -2.245303 | 3.683588 | -3.242912 |
| H | -3.187467 | 3.170461 | -3.025777 |
| H | -1.950659 | 3.431364 | -4.267001 |
| H | -2.434723 | 4.764075 | -3.202828 |
| C | -1.304207 | 3.968059 | -0.331189 |
| H | -0.507049 | 3.774317 | 0.395340 |
| H | -2.219602 | 3.530337 | 0.080381 |
| H | -1.445581 | 5.053444 | -0.411856 |
| C | 0.683950 | 4.109838 | -2.648042 |
| H | 0.471184 | 5.178625 | -2.778810 |
| H | 0.999284 | 3.713896 | -3.620600 |
| H | 1.524331 | 4.031301 | -1.948507 |
| C | 2.260423 | -2.451650 | 2.666821 |
| C | 1.319686 | -3.607810 | 2.335980 |
| H | 1.794712 | -4.090111 | 1.468441 |
| C | 1.302299 | -4.652149 | 3.465709 |
| H | 0.790311 | -4.257416 | 4.349286 |
| H | 2.313244 | -4.953802 | 3.758957 |
| H | 0.756566 | -5.544587 | 3.140281 |
| C | -0.106826 | -3.253551 | 1.906264 |
| H | -0.545001 | -4.112966 | 1.386517 |
| H | -0.118912 | -2.396790 | 1.224322 |
| H | -0.748605 | -3.030396 | 2.764164 |
| C | 3.628134 | -2.699558 | 2.506578 |
| H | 3.943020 | -3.683912 | 2.165780 |
| C | 4.582109 | -1.724681 | 2.773973 |
| H | 5.640209 | -1.953263 | 2.673439 |
| C | 4.176532 | -0.453102 | 3.169297 |
| H | 2.007178 | 2.142063 | -0.575724 |
| H | 4.924546 | 0.309739 | 3.359806 |
| C | 2.827869 | -0.152786 | 3.357835 |
| C | 2.405580 | 1.211527 | 3.873858 |
| H | 1.423431 | 1.437235 | 3.434661 |
| C | 3.364272 | 2.334394 | 3.462065 |
| H | 4.302333 | 2.296051 | 4.028167 |
| H | 2.900889 | 3.303426 | 3.675054 |
| H | 3.602604 | 2.295304 | 2.394531 |
| C | 2.261541 | 1.202301 | 5.405496 |
| H | 3.221084 | 0.957646 | 5.876637 |
| H | 1.517796 | 0.477275 | 5.747511 |
| H | 1.953757 | 2.193976 | 5.757022 |
| C | 1.880926 | -1.175036 | 3.118845 |
| C | -3.810544 | -1.590445 | 3.108048 |
| H | -2.817490 | -1.714272 | 2.657440 |
| C | -4.842635 | -1.786435 | 1.995083 |
| H | -5.864855 | -1.816160 | 2.389014 |
| H | -4.659820 | -2.740299 | 1.490300 |
| H | -4.776936 | -0.989530 | 1.249710 |
| C | -3.995058 | -2.671088 | 4.185585 |
| H | -3.221717 | -2.616392 | 4.956745 |
| H | -3.950606 | -3.668685 | 3.734112 |
| H | -4.970482 | -2.559549 | 4.673576 |
| H | 1.795793 | 2.182341 | 1.026484 |
| H | 3.376260 | 0.390202 | 0.094500 |
| N | 3.572977 | -0.638296 | -0.109033 |
| H | 3.995218 | -0.774800 | -1.028344 |
| H | 4.107119 | -1.081874 | 0.638100 |
| H | 2.524382 | -0.961002 | -0.095132 |

Calculated energies and coordinates of **H**

Electronic energy ... -4113.86291401 Eh
Total Enthalpy ... -4112.55936583 Eh
Final Gibbs free energy ... -4112.74338748 Eh

CARTESIAN COORDINATES (ANGSTROEM)

| | | | |
|---|---|---|---|
| Si | 0.672752 | 0.092918 | -3.630351 |
| Si | -0.715427 | 0.816266 | -1.851374 |
| Si | 0.570399 | 0.350248 | 0.099113 |
| P | -0.955451 | 0.649916 | 1.692758 |
| Si | -2.838239 | -0.238226 | -1.807846 |
| Si | -1.050151 | 3.159569 | -1.968256 |

| | | | |
|---|---|---|---|
| N | -1.337765 | -0.274400 | 4.232187 |
| N | 0.673452 | -0.852174 | 3.630966 |
| N | 1.971279 | 1.394277 | 0.234432 |
| C | -0.191592 | 3.102911 | -9.009978 |
| H | -1.158818 | 2.849225 | -9.453851 |
| H | 0.593369 | 2.869593 | -9.735321 |
| H | -0.178938 | 4.188129 | -8.849439 |
| C | 0.024148 | 2.369370 | -7.714553 |
| C | 1.307065 | 2.012307 | -7.294446 |
| H | 2.160555 | 2.243735 | -7.928516 |
| C | 1.509042 | 1.360750 | -6.081575 |
| H | 2.520860 | 1.088234 | -5.789496 |
| C | 0.434960 | 1.044673 | -5.238826 |
| C | -0.445616 | -0.185463 | 3.177912 |
| C | -2.573498 | 0.450554 | 4.308622 |
| C | -2.540092 | 1.746998 | 4.837620 |
| C | -1.245729 | 2.420726 | 5.251307 |
| H | -0.420393 | 1.725179 | 5.066237 |
| C | -1.239228 | 2.754485 | 6.748466 |
| H | -2.018619 | 3.483054 | 6.997821 |
| H | -0.273934 | 3.185653 | 7.036060 |
| H | -1.409841 | 1.859542 | 7.356381 |
| C | -4.940253 | 1.834571 | 4.542848 |
| H | -5.876027 | 2.378512 | 4.636301 |
| C | -4.938860 | 0.555219 | 3.998240 |
| H | -5.873635 | 0.118800 | 3.662028 |
| C | -3.752699 | -0.164628 | 3.863081 |
| C | -3.754482 | 2.426308 | 4.951738 |
| H | -3.767419 | 3.434269 | 5.356970 |
| C | -0.979781 | 3.668986 | 4.398495 |
| H | -0.986935 | 3.412581 | 3.334237 |
| H | -0.000510 | 4.092736 | 4.649889 |
| H | -1.735779 | 4.442361 | 4.575011 |
| C | -0.775402 | -0.954925 | 5.299855 |
| H | -1.309259 | -1.094628 | 6.226310 |
| C | 0.465833 | -1.323692 | 4.924610 |
| H | 1.237714 | -1.860062 | 5.452120 |
| C | 0.337879 | -1.735166 | -3.967828 |
| C | 0.264268 | -2.239515 | -5.272980 |
| H | 0.396467 | -1.565554 | -6.116846 |
| C | 0.021972 | -3.588168 | -5.513201 |
| H | -0.028837 | -3.950525 | -6.538021 |
| C | -0.159815 | -4.486799 | -4.459593 |
| C | -0.088048 | -3.992142 | -3.154943 |
| H | -0.232619 | -4.670019 | -2.315087 |
| C | 0.153076 | -2.642897 | -2.916051 |
| H | 0.190213 | -2.289269 | -1.886447 |
| C | -0.460479 | -5.937268 | -4.721289 |
| H | -1.540919 | -6.096870 | -4.824431 |
| H | -0.112364 | -6.571154 | -3.900391 |
| H | 0.010948 | -6.280641 | -5.646947 |
| C | -1.052928 | 2.044773 | -6.885755 |
| H | -2.063702 | 2.303327 | -7.195190 |
| C | -0.849069 | 1.398690 | -5.672617 |
| H | -1.708521 | 1.158753 | -5.050932 |
| C | 2.469737 | 0.285220 | -3.069777 |
| C | 3.114240 | 1.530993 | -3.065578 |
| H | 2.625767 | 2.394244 | -3.509518 |
| C | 4.369849 | 1.696101 | -2.490102 |
| H | 4.840146 | 2.677443 | -2.500464 |
| C | 5.030765 | 0.623846 | -1.882657 |
| C | 6.350427 | 0.817759 | -1.188569 |
| H | 6.193804 | 1.060112 | -0.129405 |
| H | 6.920907 | 1.638722 | -1.632740 |
| H | 6.960711 | -0.089446 | -1.230574 |
| C | 4.410077 | -0.626092 | -1.911800 |
| H | 4.909047 | -1.482113 | -1.461828 |
| C | 3.160196 | -0.791012 | -2.498875 |
| H | 2.711607 | -1.780692 | -2.512939 |
| C | -4.041304 | 0.824515 | -0.807533 |
| H | -4.171450 | 1.824721 | -1.232731 |
| H | -3.685646 | 0.937474 | 0.223106 |
| H | -5.027460 | 0.342662 | -0.782049 |
| C | -2.792895 | -1.940320 | -0.991091 |
| H | -3.817493 | -2.321738 | -0.894969 |
| H | -2.359162 | -1.872966 | 0.013154 |
| H | -2.217813 | -2.662731 | -1.577254 |
| C | -3.533563 | -0.450384 | -3.554797 |
| H | -2.848136 | -1.022022 | -4.191208 |
| H | -3.725262 | 0.514876 | -4.036810 |
| H | -4.486280 | -0.993447 | -3.513449 |
| C | -2.423547 | 3.618719 | -3.187589 |
| H | -3.342383 | 3.046731 | -3.025927 |
| H | -2.098392 | 3.454314 | -4.220072 |
| H | -2.668352 | 4.682885 | -3.078576 |
| C | -1.518865 | 3.829437 | -0.264810 |
| H | -0.736924 | 3.628302 | 0.475825 |
| H | -2.437679 | 3.371432 | 0.115725 |
| H | -1.671976 | 4.914861 | -0.319912 |
| C | 0.501909 | 4.070650 | -2.549116 |
| H | 0.325334 | 5.153538 | -2.527175 |
| H | 0.751256 | 3.793399 | -3.579800 |
| H | 1.369857 | 3.856535 | -1.916544 |
| C | 2.249949 | -2.293394 | 2.437850 |
| C | 1.332950 | -3.510666 | 2.378406 |
| H | 1.678125 | -4.062858 | 1.494314 |
| C | 1.563636 | -4.436179 | 3.585627 |
| H | 1.188737 | -3.982315 | 4.508958 |
| H | 2.626431 | -4.661940 | 3.718958 |
| H | 1.027131 | -5.380324 | 3.439834 |
| C | -0.165287 | -3.259199 | 2.172468 |
| H | -0.632160 | -4.182976 | 1.812896 |
| H | -0.354392 | -2.474882 | 1.433050 |
| H | -0.672276 | -2.982821 | 3.102702 |
| C | 3.539970 | -2.464010 | 1.924079 |
| H | 3.806134 | -3.431888 | 1.505939 |
| C | 4.465129 | -1.432330 | 1.938244 |
| H | 5.460134 | -1.589515 | 1.529880 |
| C | 4.123447 | -0.194978 | 2.465783 |
| H | 2.679525 | 1.258519 | -0.480853 |
| H | 4.851774 | 0.608187 | 2.455551 |
| C | 2.856596 | 0.033355 | 2.998730 |
| C | 2.494794 | 1.378815 | 3.608067 |
| H | 1.463196 | 1.603530 | 3.303750 |
| C | 3.374422 | 2.529508 | 3.111652 |
| H | 4.385852 | 2.475572 | 3.532072 |

| | | | |
|---|---|---|---|
| H | 2.941488 | 3.481757 | 3.436087 |
| H | 3.443476 | 2.532446 | 2.021417 |
| C | 2.556941 | 1.336525 | 5.145339 |
| H | 3.571416 | 1.086118 | 5.477283 |
| H | 1.867208 | 0.605897 | 5.574832 |
| H | 2.300577 | 2.320465 | 5.555811 |
| C | 1.937162 | -1.033779 | 2.971631 |
| C | -3.727628 | -1.559490 | 3.267591 |
| H | -2.758773 | -1.676995 | 2.765866 |
| C | -4.811526 | -1.776562 | 2.209524 |
| H | -5.812854 | -1.816680 | 2.653047 |
| H | -4.641801 | -2.730976 | 1.700932 |
| H | -4.793623 | -0.982879 | 1.457298 |
| C | -3.839713 | -2.627935 | 4.366198 |
| H | -3.027674 | -2.551299 | 5.095459 |
| H | -3.802606 | -3.630619 | 3.925858 |
| H | -4.789643 | -2.524261 | 4.903481 |
| H | 1.750092 | 2.382112 | 0.280331 |
| H | 2.551197 | -3.178536 | -0.642398 |
| N | 2.738188 | -4.128113 | -0.955422 |
| H | 2.253202 | -4.225116 | -1.842948 |
| H | 3.729195 | -4.154058 | -1.174811 |
| H | 1.135307 | -1.027596 | -0.037607 |

Calculated energies and coordinates of **I**

Electronic energy ... -4113.81057979 Eh
Total Enthalpy ... -4112.50446237 Eh
Final Gibbs free energy ... -4112.68754869 Eh

CARTESIAN COORDINATES (ANGSTROEM)

| | | | |
|---|---|---|---|
| Si | 0.768924 | -0.420281 | -3.544947 |
| Si | -0.720084 | 0.381266 | -1.860781 |
| Si | 0.099868 | 0.044464 | 0.387874 |
| P | -1.221114 | 1.084097 | 1.842263 |
| Si | -2.834377 | -0.686192 | -2.031339 |
| Si | -1.090935 | 2.714436 | -2.210292 |
| N | -1.549757 | -0.028717 | 4.378473 |
| N | 0.342632 | -0.675816 | 3.498281 |
| N | 1.670098 | 1.143113 | 0.252138 |
| C | 0.196713 | 2.855657 | -8.816818 |
| H | -0.577699 | 2.436282 | -9.466550 |
| H | 1.134115 | 2.895580 | -9.378780 |
| H | -0.094968 | 3.887729 | -8.586080 |
| C | 0.347254 | 2.050258 | -7.555594 |
| C | 1.596915 | 1.858032 | -6.965162 |
| H | 2.482303 | 2.273511 | -7.441948 |
| C | 1.730317 | 1.137115 | -5.781723 |
| H | 2.722389 | 0.997762 | -5.359465 |
| C | 0.615790 | 0.586372 | -5.136104 |
| C | -0.769388 | 0.086336 | 3.248285 |
| C | -2.717474 | 0.759410 | 4.647297 |
| C | -2.546841 | 1.980789 | 5.311724 |
| C | -1.177082 | 2.527944 | 5.666850 |
| H | -0.419688 | 1.817187 | 5.318695 |
| C | -1.010067 | 2.676910 | 7.184748 |
| H | -1.716382 | 3.408550 | 7.592538 |
| H | 0.002564 | 3.019743 | 7.423919 |
| H | -1.180243 | 1.724442 | 7.697761 |
| C | -4.952591 | 2.243665 | 5.271905 |
| H | -5.835597 | 2.824955 | 5.522598 |
| C | -5.089614 | 1.043801 | 4.582548 |
| H | -6.079020 | 0.706240 | 4.291583 |
| C | -3.973936 | 0.277828 | 4.248919 |
| C | -3.695716 | 2.711120 | 5.624545 |
| H | -3.601254 | 3.661975 | 6.141708 |
| C | -0.915048 | 3.855818 | 4.942804 |
| H | -1.028143 | 3.731568 | 3.861256 |
| H | 0.104378 | 4.201536 | 5.148367 |
| H | -1.608230 | 4.636031 | 5.276653 |
| C | -0.905657 | -0.813524 | 5.323810 |
| H | -1.338747 | -0.997183 | 6.294444 |
| C | 0.252219 | -1.237760 | 4.766946 |
| H | 1.019007 | -1.895930 | 5.142727 |
| C | 0.350131 | -2.217324 | -3.928894 |
| C | 0.035340 | -2.660368 | -5.218842 |
| H | 0.022343 | -1.951425 | -6.043696 |
| C | -0.256283 | -3.998914 | -5.468633 |
| H | -0.499115 | -4.314830 | -6.481059 |
| C | -0.233653 | -4.945583 | -4.443154 |
| C | 0.090850 | -4.511526 | -3.154215 |
| H | 0.115938 | -5.228211 | -2.335524 |
| C | 0.366322 | -3.173941 | -2.904448 |
| H | 0.593436 | -2.865095 | -1.885575 |
| C | -0.575436 | -6.386587 | -4.706150 |
| H | -0.436527 | -6.643524 | -5.760066 |
| H | -1.622813 | -6.590007 | -4.450986 |
| H | 0.042762 | -7.058329 | -4.102559 |
| C | -0.767653 | 1.487757 | -6.927496 |
| H | -1.753167 | 1.612291 | -7.372321 |
| C | -0.634351 | 0.776743 | -5.741632 |
| H | -1.523712 | 0.356052 | -5.279660 |
| C | 2.583387 | -0.338236 | -3.016497 |
| C | 3.179873 | 0.891197 | -2.695982 |
| H | 2.596000 | 1.808045 | -2.757656 |
| C | 4.518273 | 0.976191 | -2.328222 |
| H | 4.947708 | 1.948353 | -2.097429 |
| C | 5.317522 | -0.167272 | -2.260346 |
| C | 6.745431 | -0.084276 | -1.797746 |
| H | 7.202144 | 0.869758 | -2.078363 |
| H | 7.350086 | -0.893575 | -2.216931 |
| H | 6.800634 | -0.165861 | -0.704231 |
| C | 4.738490 | -1.390454 | -2.602241 |
| H | 5.344187 | -2.294027 | -2.573652 |
| C | 3.400578 | -1.474684 | -2.974230 |
| H | 2.985877 | -2.444582 | -3.238546 |
| C | -4.034843 | 0.249703 | -0.915044 |
| H | -4.200440 | 1.274116 | -1.266196 |
| H | -3.646022 | 0.308933 | 0.108305 |
| H | -5.006397 | -0.260119 | -0.891498 |
| C | -2.787743 | -2.497545 | -1.506112 |
| H | -3.815282 | -2.868140 | -1.397715 |
| H | -2.273008 | -2.648605 | -0.554355 |
| H | -2.283826 | -3.106906 | -2.262853 |
| C | -3.515781 | -0.685741 | -3.795852 |

| | | | |
|---|---|---|---|
| H | -2.888861 | -1.294288 | -4.457838 |
| H | -3.608109 | 0.318089 | -4.221727 |
| H | -4.516571 | -1.136645 | -3.785539 |
| C | -2.420829 | 3.023871 | -3.519428 |
| H | -3.379707 | 2.575561 | -3.240205 |
| H | -2.129495 | 2.637336 | -4.500172 |
| H | -2.575810 | 4.106270 | -3.616137 |
| C | -1.675033 | 3.579107 | -0.637245 |
| H | -1.904163 | 4.626585 | -0.872898 |
| H | -0.921914 | 3.567725 | 0.157692 |
| H | -2.576813 | 3.117244 | -0.222705 |
| C | 0.476220 | 3.603465 | -2.791806 |
| H | 0.238725 | 4.659003 | -2.976098 |
| H | 0.859251 | 3.179211 | -3.726612 |
| H | 1.277431 | 3.580273 | -2.043627 |
| C | 1.116998 | -1.452379 | 1.256111 |
| C | 0.332540 | -2.804098 | 1.299943 |
| H | 0.257457 | -3.111441 | 0.244583 |
| C | 1.148185 | -3.868653 | 2.040065 |
| H | 1.262736 | -3.592245 | 3.095233 |
| H | 2.151592 | -3.966389 | 1.614825 |
| H | 0.644812 | -4.841200 | 1.996767 |
| C | -1.095844 | -2.767120 | 1.849981 |
| H | -1.627165 | -3.683138 | 1.566160 |
| H | -1.659114 | -1.912576 | 1.457771 |
| H | -1.110666 | -2.712405 | 2.943800 |
| C | 2.394866 | -1.652695 | 0.486394 |
| H | 2.319220 | -2.202650 | -0.448962 |
| C | 3.606293 | -1.184792 | 0.888914 |
| H | 4.466979 | -1.379521 | 0.251145 |
| C | 3.786340 | -0.451925 | 2.091552 |
| H | 2.276509 | 0.875985 | -0.529537 |
| H | 4.775245 | -0.131246 | 2.400367 |
| C | 2.687673 | -0.319344 | 2.958940 |
| C | 2.891140 | 0.420009 | 4.275947 |
| H | 1.928036 | 0.507234 | 4.788650 |
| C | 3.391005 | 1.853186 | 4.047183 |
| H | 4.389119 | 1.859226 | 3.596389 |
| H | 3.449660 | 2.391840 | 5.000625 |
| H | 2.708728 | 2.403009 | 3.388260 |
| C | 3.844619 | -0.343634 | 5.204615 |
| H | 4.849146 | -0.401146 | 4.772901 |
| H | 3.500562 | -1.370433 | 5.367587 |
| H | 3.919808 | 0.154781 | 6.178182 |
| C | 1.442963 | -0.835928 | 2.598304 |
| C | -4.099206 | -1.029714 | 3.492906 |
| H | -3.199170 | -1.125519 | 2.875357 |
| C | -5.301821 | -1.063559 | 2.546776 |
| H | -6.249404 | -1.141088 | 3.092281 |
| H | -5.229209 | -1.939960 | 1.893976 |
| H | -5.336481 | -0.171703 | 1.914027 |
| C | -4.146440 | -2.223322 | 4.459310 |
| H | -3.251706 | -2.270692 | 5.087194 |
| H | -4.213855 | -3.162496 | 3.898813 |
| H | -5.021830 | -2.150967 | 5.115464 |
| H | 1.464758 | 2.136404 | 0.171249 |
| H | 2.239728 | 1.019174 | 1.100363 |
| N | 4.235507 | 2.887162 | 0.695481 |
| H | 5.002647 | 3.160478 | 0.090643 |
| H | 4.335500 | 3.439728 | 1.540801 |
| H | 4.417688 | 1.922862 | 0.973842 |

Calculated energies and coordinates of **TS[I-J]**

Electronic energy ... -4113.79644857 Eh
Total Enthalpy ... -4112.49594368 Eh
Final Gibbs free energy ... -4112.67246289 Eh

CARTESIAN COORDINATES (ANGSTROEM)

| | | | |
|---|---|---|---|
| Si | 0.883726 | -0.391827 | -3.501261 |
| Si | -0.721214 | 0.305049 | -1.857680 |
| Si | 0.166829 | 0.408870 | 0.401337 |
| P | -1.172878 | 1.398790 | 1.880877 |
| Si | -2.692469 | -1.000544 | -2.088654 |
| Si | -1.345540 | 2.571602 | -2.331782 |
| N | -1.581826 | 0.188367 | 4.344374 |
| N | 0.307293 | -0.503572 | 3.478993 |
| N | 1.498397 | 1.641681 | 0.066980 |
| C | -0.049956 | 2.613430 | -8.881901 |
| H | -0.954870 | 2.260124 | -9.385107 |
| H | 0.794415 | 2.496798 | -9.567563 |
| H | -0.176556 | 3.687094 | -8.695354 |
| C | 0.183664 | 1.872746 | -7.593901 |
| C | 1.475021 | 1.664429 | -7.105710 |
| H | 2.327117 | 2.018436 | -7.682526 |
| C | 1.690224 | 1.006288 | -5.898741 |
| H | 2.710864 | 0.855417 | -5.555804 |
| C | 0.619864 | 0.536158 | -5.126322 |
| C | -0.762444 | 0.325609 | 3.240130 |
| C | -2.694773 | 1.039973 | 4.647092 |
| C | -2.442396 | 2.224541 | 5.351348 |
| C | -1.038912 | 2.664334 | 5.722607 |
| H | -0.329644 | 1.935530 | 5.316139 |
| C | -0.851801 | 2.695384 | 7.245543 |
| H | -1.511732 | 3.435421 | 7.711958 |
| H | 0.180998 | 2.960615 | 7.497134 |
| H | -1.073876 | 1.719866 | 7.690811 |
| C | -4.826935 | 2.636951 | 5.353351 |
| H | -5.669250 | 3.262769 | 5.635023 |
| C | -5.046096 | 1.469820 | 4.630883 |
| H | -6.058464 | 1.201834 | 4.346675 |
| C | -3.983903 | 0.647600 | 4.257254 |
| C | -3.539196 | 3.013686 | 5.703531 |
| H | -3.379817 | 3.936856 | 6.253819 |
| C | -0.702446 | 4.021358 | 5.090019 |
| H | -0.830991 | 3.978565 | 4.004056 |
| H | 0.337325 | 4.290642 | 5.307673 |
| H | -1.343621 | 4.817340 | 5.484839 |
| C | -1.023253 | -0.701113 | 5.246888 |
| H | -1.496939 | -0.924436 | 6.189673 |
| C | 0.121504 | -1.160486 | 4.694072 |
| H | 0.812999 | -1.912324 | 5.036173 |
| C | 0.633435 | -2.233818 | -3.839396 |
| C | 0.105212 | -2.674002 | -5.059961 |
| H | -0.110426 | -1.952989 | -5.845095 |

| | | | |
|---|---|---|---|
| C | -0.147900 | -4.022577 | -5.295811 |
| H | -0.561464 | -4.329743 | -6.254124 |
| C | 0.127173 | -4.987898 | -4.326869 |
| C | 0.668451 | -4.559331 | -3.111711 |
| H | 0.893408 | -5.288764 | -2.336101 |
| C | 0.905297 | -3.212559 | -2.870811 |
| H | 1.301492 | -2.928072 | -1.899127 |
| C | -0.175346 | -6.441735 | -4.564775 |
| H | -0.260605 | -6.662392 | -5.632475 |
| H | -1.124036 | -6.722321 | -4.090757 |
| H | 0.602476 | -7.084166 | -4.140263 |
| C | -0.887919 | 1.391502 | -6.837679 |
| H | -1.904814 | 1.530005 | -7.200132 |
| C | -0.671022 | 0.742491 | -5.628356 |
| H | -1.526602 | 0.385325 | -5.061888 |
| C | 2.690379 | -0.069806 | -3.017115 |
| C | 3.147518 | 1.243323 | -2.840603 |
| H | 2.473144 | 2.077808 | -3.027487 |
| C | 4.463030 | 1.517201 | -2.471531 |
| H | 4.786557 | 2.551547 | -2.363254 |
| C | 5.378773 | 0.482912 | -2.267056 |
| C | 6.797047 | 0.759627 | -1.846621 |
| H | 6.986364 | 1.833206 | -1.756248 |
| H | 7.510064 | 0.352460 | -2.571910 |
| H | 7.021323 | 0.290018 | -0.881031 |
| C | 4.940942 | -0.827606 | -2.478731 |
| H | 5.641367 | -1.650848 | -2.351482 |
| C | 3.630097 | -1.097096 | -2.851217 |
| H | 3.333236 | -2.129821 | -3.016201 |
| C | -3.934765 | -0.292992 | -0.854295 |
| H | -4.256576 | 0.711694 | -1.152113 |
| H | -3.499690 | -0.205625 | 0.147622 |
| H | -4.827568 | -0.927559 | -0.794048 |
| C | -2.451612 | -2.847061 | -1.790033 |
| H | -3.437916 | -3.328606 | -1.761623 |
| H | -1.936049 | -3.078042 | -0.856603 |
| H | -1.881323 | -3.293046 | -2.611440 |
| C | -3.463793 | -0.920885 | -3.816914 |
| H | -2.825793 | -1.418175 | -4.556397 |
| H | -3.677932 | 0.094453 | -4.161234 |
| H | -4.414808 | -1.468987 | -3.788173 |
| C | -2.805351 | 2.740124 | -3.527127 |
| H | -3.699860 | 2.234886 | -3.148948 |
| H | -2.589162 | 2.363092 | -4.529762 |
| H | -3.043969 | 3.807769 | -3.617321 |
| C | -1.905239 | 3.491064 | -0.778998 |
| H | -2.233710 | 4.497317 | -1.070623 |
| H | -1.116529 | 3.602080 | -0.028007 |
| H | -2.742292 | 2.991055 | -0.281072 |
| C | 0.083153 | 3.575658 | -3.068707 |
| H | -0.280201 | 4.583148 | -3.308459 |
| H | 0.469896 | 3.130801 | -3.991818 |
| H | 0.912902 | 3.693017 | -2.361824 |
| C | 1.059842 | -1.132553 | 1.181449 |
| C | 0.211369 | -2.454910 | 1.094653 |
| H | 0.152052 | -2.669824 | 0.015277 |
| C | 0.952266 | -3.612949 | 1.766758 |
| H | 1.025056 | -3.440868 | 2.847811 |
| H | 1.969891 | -3.712568 | 1.377643 |
| H | 0.418137 | -4.557395 | 1.611250 |
| C | -1.228412 | -2.388433 | 1.614996 |
| H | -1.774646 | -3.292673 | 1.321618 |
| H | -1.769603 | -1.522327 | 1.216575 |
| H | -1.264167 | -2.339465 | 2.708340 |
| C | 2.334643 | -1.331594 | 0.387115 |
| H | 2.221412 | -1.533542 | -0.673326 |
| C | 3.571057 | -1.366089 | 0.941879 |
| H | 4.418120 | -1.598509 | 0.294957 |
| C | 3.817916 | -1.009553 | 2.297236 |
| H | 1.778599 | 1.600755 | -0.914421 |
| H | 4.802011 | -1.145387 | 2.733411 |
| C | 2.705215 | -0.687732 | 3.118596 |
| C | 3.006598 | -0.211220 | 4.536867 |
| H | 2.071711 | 0.066288 | 5.032666 |
| C | 3.872079 | 1.057962 | 4.526296 |
| H | 4.850315 | 0.870965 | 4.070312 |
| H | 4.038577 | 1.414662 | 5.549722 |
| H | 3.370010 | 1.858417 | 3.970639 |
| C | 3.678076 | -1.305779 | 5.377557 |
| H | 4.673057 | -1.544760 | 4.988778 |
| H | 3.097143 | -2.234185 | 5.366730 |
| H | 3.791225 | -0.980601 | 6.418371 |
| C | 1.421123 | -0.724488 | 2.606157 |
| C | -4.205353 | -0.631398 | 3.474641 |
| H | -3.308463 | -0.792394 | 2.865251 |
| C | -5.398549 | -0.548326 | 2.520251 |
| H | -6.352647 | -0.535091 | 3.059372 |
| H | -5.406557 | -1.426683 | 1.866948 |
| H | -5.341555 | 0.342699 | 1.887593 |
| C | -4.364939 | -1.833078 | 4.419336 |
| H | -3.484968 | -1.967765 | 5.054870 |
| H | -4.506162 | -2.753277 | 3.841455 |
| H | -5.238405 | -1.697081 | 5.067903 |
| H | 1.089875 | 2.563292 | 0.219835 |
| H | 2.740706 | 1.632891 | 0.688324 |
| N | 3.901222 | 1.625286 | 1.061651 |
| H | 4.490095 | 1.538564 | 0.235685 |
| H | 4.178522 | 2.443163 | 1.594172 |
| H | 4.019600 | 0.740820 | 1.643589 |

Calculated energies and coordinates of **J**

Electronic energy ... -4113.84770203 Eh
Total Enthalpy ... -4112.54130619 Eh
Final Gibbs free energy ... -4112.72225023 Eh

CARTESIAN COORDINATES (ANGSTROEM)

| | | | |
|---|---|---|---|
| Si | 0.884044 | -0.467486 | -3.449707 |
| Si | -0.674874 | 0.282174 | -1.787246 |
| Si | 0.249011 | 0.437154 | 0.435204 |
| P | -1.244378 | 1.267114 | 1.883330 |
| Si | -2.687533 | -0.964364 | -2.045607 |
| Si | -1.215292 | 2.579919 | -2.237123 |
| N | -1.656762 | 0.021306 | 4.330192 |
| N | 0.205184 | -0.680339 | 3.441398 |

| | | | |
|---|---|---|---|
| N | 1.595198 | 1.568107 | 0.321930 |
| C | 0.060667 | 2.665628 | -8.774968 |
| H | -0.842761 | 2.339070 | -9.298854 |
| H | 0.912374 | 2.551645 | -9.451768 |
| H | -0.052229 | 3.736330 | -8.564322 |
| C | 0.264475 | 1.890993 | -7.501921 |
| C | 1.546270 | 1.609490 | -7.025587 |
| H | 2.411997 | 1.931379 | -7.600807 |
| C | 1.734867 | 0.921438 | -5.830842 |
| H | 2.748296 | 0.717688 | -5.493984 |
| C | 0.646353 | 0.492047 | -5.060413 |
| C | -0.851212 | 0.172009 | 3.218493 |
| C | -2.746886 | 0.891743 | 4.661239 |
| C | -2.455501 | 2.067116 | 5.365953 |
| C | -1.036258 | 2.472415 | 5.716196 |
| H | -0.348311 | 1.746201 | 5.269726 |
| C | -0.812929 | 2.449365 | 7.234524 |
| H | -1.452222 | 3.181999 | 7.739790 |
| H | 0.229158 | 2.692867 | 7.469214 |
| H | -1.038086 | 1.462587 | 7.652838 |
| C | -4.830824 | 2.528477 | 5.406197 |
| H | -5.655013 | 3.170553 | 5.704259 |
| C | -5.086905 | 1.372119 | 4.678418 |
| H | -6.108840 | 1.129727 | 4.405756 |
| C | -4.048119 | 0.530066 | 4.283575 |
| C | -3.530199 | 2.876476 | 5.739103 |
| H | -3.343439 | 3.793533 | 6.290879 |
| C | -0.687103 | 3.842668 | 5.120912 |
| H | -0.854963 | 3.844669 | 4.039502 |
| H | 0.367429 | 4.074118 | 5.307808 |
| H | -1.289561 | 4.640715 | 5.568901 |
| C | -1.101712 | -0.898429 | 5.210063 |
| H | -1.574891 | -1.140289 | 6.148299 |
| C | 0.039050 | -1.351093 | 4.647565 |
| H | 0.748923 | -2.091486 | 4.980553 |
| C | 0.560716 | -2.294561 | -3.816162 |
| C | -0.038291 | -2.690332 | -5.019160 |
| H | -0.268203 | -1.944277 | -5.776395 |
| C | -0.341441 | -4.024807 | -5.274843 |
| H | -0.808412 | -4.295874 | -6.219422 |
| C | -0.048397 | -5.022023 | -4.344067 |
| C | 0.565716 | -4.639623 | -3.148348 |
| H | 0.812128 | -5.395918 | -2.405613 |
| C | 0.854366 | -3.306359 | -2.889276 |
| H | 1.317822 | -3.057841 | -1.937849 |
| C | -0.404259 | -6.460115 | -4.602634 |
| H | -0.527907 | -6.654272 | -5.671717 |
| H | -1.347634 | -6.720890 | -4.107076 |
| H | 0.364509 | -7.136190 | -4.215662 |
| C | -0.825910 | 1.452175 | -6.746824 |
| H | -1.836611 | 1.649910 | -7.099111 |
| C | -0.635146 | 0.771929 | -5.550410 |
| H | -1.504427 | 0.451126 | -4.983671 |
| C | 2.701617 | -0.229807 | -2.981473 |
| C | 3.188044 | 1.064644 | -2.750291 |
| H | 2.515135 | 1.914836 | -2.841031 |
| C | 4.525650 | 1.297133 | -2.449285 |
| H | 4.871871 | 2.318005 | -2.299264 |

| | | | |
|---|---|---|---|
| C | 5.433636 | 0.239646 | -2.353640 |
| C | 6.867800 | 0.477762 | -1.967854 |
| H | 7.175272 | 1.505157 | -2.183944 |
| H | 7.541571 | -0.202368 | -2.497787 |
| H | 7.015006 | 0.308746 | -0.893114 |
| C | 4.962806 | -1.050078 | -2.609039 |
| H | 5.655482 | -1.888608 | -2.569262 |
| C | 3.627117 | -1.279415 | -2.924068 |
| H | 3.305705 | -2.295247 | -3.141180 |
| C | -3.926097 | -0.290193 | -0.789279 |
| H | -4.159977 | 0.760572 | -0.996300 |
| H | -3.533444 | -0.330809 | 0.232017 |
| H | -4.863599 | -0.858888 | -0.829719 |
| C | -2.493008 | -2.831896 | -1.840957 |
| H | -3.490671 | -3.284411 | -1.768804 |
| H | -1.923822 | -3.128332 | -0.959287 |
| H | -1.993593 | -3.252967 | -2.719625 |
| C | -3.471069 | -0.802621 | -3.763828 |
| H | -2.854258 | -1.289644 | -4.527394 |
| H | -3.668086 | 0.227363 | -4.072162 |
| H | -4.433050 | -1.331644 | -3.740014 |
| C | -2.691771 | 2.806852 | -3.401721 |
| H | -3.596643 | 2.332821 | -3.007735 |
| H | -2.511004 | 2.429910 | -4.411350 |
| H | -2.892625 | 3.883171 | -3.480564 |
| C | -1.711659 | 3.513592 | -0.671363 |
| H | -1.975990 | 4.541112 | -0.953775 |
| H | -0.910712 | 3.566141 | 0.072298 |
| H | -2.578650 | 3.064638 | -0.176456 |
| C | 0.243868 | 3.523624 | -2.984210 |
| H | -0.071189 | 4.552245 | -3.201460 |
| H | 0.598863 | 3.075472 | -3.918011 |
| H | 1.081203 | 3.579880 | -2.279446 |
| C | 1.052407 | -1.198830 | 1.172389 |
| C | 0.203189 | -2.500906 | 0.999900 |
| H | 0.138518 | -2.640991 | -0.089679 |
| C | 0.942053 | -3.707555 | 1.588708 |
| H | 1.017733 | -3.614043 | 2.679451 |
| H | 1.958226 | -3.793803 | 1.191640 |
| H | 0.400721 | -4.634903 | 1.370811 |
| C | -1.238745 | -2.479459 | 1.519164 |
| H | -1.762048 | -3.375195 | 1.166790 |
| H | -1.788992 | -1.602718 | 1.163876 |
| H | -1.287264 | -2.497226 | 2.612305 |
| C | 2.323206 | -1.356625 | 0.396341 |
| H | 2.212816 | -1.652692 | -0.644172 |
| C | 3.545151 | -1.140575 | 0.874733 |
| H | 4.404172 | -1.264750 | 0.216811 |
| C | 3.808978 | -0.723158 | 2.283366 |
| H | 2.193456 | 1.470902 | -0.492850 |
| H | 4.428199 | -1.487675 | 2.784346 |
| C | 2.562774 | -0.497432 | 3.094668 |
| C | 2.759984 | 0.190923 | 4.433344 |
| H | 1.851075 | 0.085553 | 5.032519 |
| C | 2.966737 | 1.700733 | 4.208703 |
| H | 3.885149 | 1.902767 | 3.647559 |
| H | 3.039565 | 2.213273 | 5.175470 |
| H | 2.124927 | 2.124594 | 3.650468 |

| | | | |
|---|---|---|---|
| C | 3.915859 | -0.403573 | 5.246387 |
| H | 4.881863 | -0.229094 | 4.761029 |
| H | 3.795211 | -1.484180 | 5.385354 |
| H | 3.956103 | 0.063445 | 6.236155 |
| C | 1.354592 | -0.818123 | 2.596403 |
| C | -4.305849 | -0.736271 | 3.490495 |
| H | -3.416635 | -0.915552 | 2.873382 |
| C | -5.502696 | -0.613932 | 2.544353 |
| H | -6.452055 | -0.581550 | 3.090756 |
| H | -5.537801 | -1.485431 | 1.882838 |
| H | -5.427448 | 0.281538 | 1.920141 |
| C | -4.490491 | -1.941212 | 4.426441 |
| H | -3.611820 | -2.101739 | 5.057896 |
| H | -4.656911 | -2.853621 | 3.843005 |
| H | -5.357336 | -1.788767 | 5.079975 |
| H | 1.322544 | 2.537952 | 0.426628 |
| H | 3.797877 | 2.164284 | 1.097530 |
| N | 4.807403 | 2.290353 | 1.036252 |
| H | 5.071409 | 1.859019 | 0.155572 |
| H | 4.972122 | 3.285507 | 0.927272 |
| H | 4.429672 | 0.185388 | 2.290826 |

# Crystalographic Details

Data were collected on a single crystal X-ray diffractometer equipped with a CPAD detector (Bruker Photon-II), a TXS rotating anode with MoKα radiation (λ = 0.71073 Å) and a Helios optic using the APEX4 software package.[16] The crystal was fixed on the top of a kapton micro sampler with perfluorinated ether and transferred to the diffractometer, and frozen under a stream of cold nitrogen. A matrix scan was used to determine the initial lattice parameters. Reflections were corrected for Lorentz and polarisation effects, scan speed, and background using SAINT.[17] Absorption correction, including odd and even ordered spherical harmonics was performed using SADABS.[18] Space group assignment was based upon systematic absences, E statistics, and successful refinement of the structure. The structures were solved using SHELXT with the aid of successive difference Fourier maps and were refined against all data using SHELXL in conjunction with SHELXLE.[18-20] Hydrogen atoms (except on heteroatoms) were calculated in ideal positions as follows: Methyl hydrogen atoms were refined as part of rigid rotating groups, with a C–H distance of 0.98 Å and Uiso(H) = 1.5·$U_{eq}$(C). Non-methyl H atoms were placed in calculated positions and refined using a riding model with methylene, aromatic, and other C–H distances of 0.99 Å, 0.95 Å, and 1.00 Å, respectively, and Uiso(H) = 1.2·$U_{eq}$(C). Non-hydrogen atoms were refined with anisotropic displacement parameters. Full-matrix least-squares refinements were carried out by minimizing $\Sigma w(F_o^2-F_c^2)^2$ with the SHELXL weighting scheme. Neutral atom scattering factors for all atoms and anomalous dispersion corrections for the non-hydrogen atoms were taken from International Tables for Crystallography.[21] Images of the crystal structure were generated with Mercury and PLATON.[22, 23] Deposition Number 2377544 contains the supplementary crystallographic data for this paper. These data are provided free of charge by the joint Cambridge Crystallographic Data Centre and Fachinformationszentrum Karlsruhe Access Structures service www.ccdc.cam.ac.uk/structures.

Compound **2** (CCDC = 2377544)

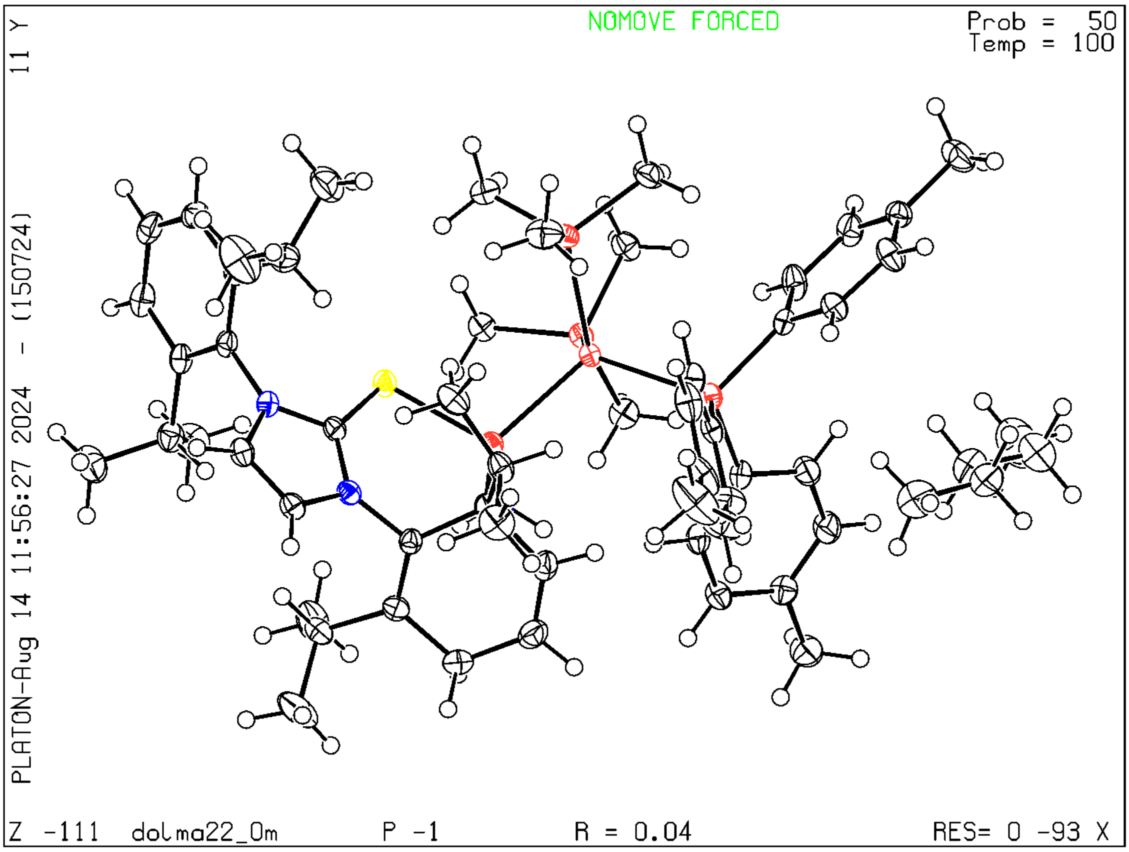


**Supplementary Figure 11:** PLATON generated image of compound **2**.

Diffractometer operator J.Y. Liu
scanspeed 8s per frame dx 41 mm
1779 frames measured in 6 data sets
phi-scans with delta_phi = 0.5
omega-scans with delta_omega = 0.5
shutterless mode

*Crystal data*

| | |
|---|---|
| $C_{54}H_{78}N_3PSi_5 \cdot C_5H_{12}$ | $F(000) = 1100$ |
| $M_r = 1012.76$ | |
| Triclinic, *P* | $D_x = 1.117$ Mg m$^{-3}$ |
| Hall symbol: -P 1 | |
| $a = 13.1621$ (7) Å | Mo *K*α radiation, λ = 0.71073 Å |
| $b = 13.9425$ (8) Å | Cell parameters from 9737 reflections |
| $c = 17.0349$ (10) Å | θ = 2.4–25.7° |
| α = 93.904 (2)° | μ = 0.18 mm$^{-1}$ |

| | |
|---|---|
| $\beta = 102.733$ (2)° | $T = 100$ K |
| $\gamma = 96.997$ (2)° | Fragment, colorless |
| $V = 3012.0$ (3) Å$^3$ | 0.23 × 0.19 × 0.18 mm |
| $Z = 2$ | |

*Data collection*

| | |
|---|---|
| Bruker Photon CMOS diffractometer | 11227 independent reflections |
| Radiation source: TXS rotating anode | 8755 reflections with $I > 2\sigma(I)$ |
| Helios optic monochromator | $R_{int} = 0.099$ |
| Detector resolution: 16 pixels mm$^{-1}$ | $\theta_{max} = 25.7°$, $\theta_{min} = 2.3°$ |
| phi– and ω–rotation scans | $h = -16$   16 |
| Absorption correction: multi-scan *SADABS* 2016/2, Bruker, 2016 | $k = -17$   17 |
| $T_{min} = 0.639$, $T_{max} = 0.745$ | $l = -20$   20 |
| 69338 measured reflections | |

*Refinement*

| | |
|---|---|
| Refinement on $F^2$ | Secondary atom site location: difference Fourier map |
| Least-squares matrix: full | Hydrogen site location: mixed |
| $R[F^2 > 2\sigma(F^2)] = 0.043$ | H atoms treated by a mixture of independent and constrained refinement |
| $wR(F^2) = 0.114$ | W = $1/[\Sigma^2(FO^2) + (0.0561P)^2 + 7.2754P]$ WHERE $P = (FO^2 + 2FC^2)/3$ |
| $S = 0.74$ | $(\Delta/\sigma)_{max} = 0.001$ |
| 11227 reflections | $\Delta\rho_{max} = 0.38$ e Å$^{-3}$ |
| 638 parameters | $\Delta\rho_{min} = -0.25$ e Å$^{-3}$ |
| 0 restraints | Extinction correction: - |
| 0 constraints | Extinction coefficient: - |
| Primary atom site location: iterative | |

# References


1. M. Muhr, P. Heiß, M. Schütz, R. Bühler, C. Gemel, M. H. Linden, H. B. Linden and R. A. Fischer, *Dalton Trans.*, 2021, **50**, 9031-9036.
2. M. E. Doleschal, A. Kostenko, J. Y. Liu and S. Inoue, *Nat. Chem.*, DOI: 10.1038/s41557-024-01618-6.
3. F. Neese, *WIREs Computational Molecular Science*, 2022, **12**, e1606.
4. J. W. Furness, A. D. Kaplan, J. Ning, J. P. Perdew and J. Sun, *The Journal of Physical Chemistry Letters*, 2020, **11**, 9248-9248.
5. J. W. Furness, A. D. Kaplan, J. Ning, J. P. Perdew and J. Sun, *The Journal of Physical Chemistry Letters*, 2020, **11**, 8208-8215.
6. H. Kruse and S. Grimme, *J. Chem. Phys.*, 2012, **136**.
7. E. Caldeweyher, C. Bannwarth and S. Grimme, *J. Chem. Phys.*, 2017, **147**.
8. E. Caldeweyher, S. Ehlert, A. Hansen, H. Neugebauer, S. Spicher, C. Bannwarth and S. Grimme, *J. Chem. Phys.*, 2019, **150**.
9. E. Caldeweyher, J.-M. Mewes, S. Ehlert and S. Grimme, *Physical Chemistry Chemical Physics*, 2020, **22**, 8499-8512.
10. S. Grimme, A. Hansen, S. Ehlert and J.-M. Mewes, *J. Chem. Phys.*, 2021, **154**.
11. A. V. Marenich, C. J. Cramer and D. G. Truhlar, *The Journal of Physical Chemistry B*, 2009, **113**, 6378-6396.
12. Y. Zhao and D. G. Truhlar, *The Journal of Physical Chemistry A*, 2005, **109**, 5656-5667.
13. F. Weigend and R. Ahlrichs, *Physical Chemistry Chemical Physics*, 2005, **7**, 3297-3305.
14. F. Weigend, *Physical Chemistry Chemical Physics*, 2006, **8**, 1057-1065.
15. A. Hellweg, C. Hättig, S. Höfener and W. Klopper, *Theoretical Chemistry Accounts*, 2007, **117**, 587-597.
16. *Bruker AXS Inc. APEX suite of crystallographic software; Madison, Wisconsin, USA*, 2021.
17. *SAINT, Version 8.40A and SADABS, Version 2016/2, Bruker AXS Inc., Madison, Wisconsin, USA*, 2016/2019.
18. G. Sheldrick, *Acta Crystallographica Section A*, 2015, **71**, 3-8.
19. C. B. Hubschle, G. M. Sheldrick and B. Dittrich, *Journal of Applied Crystallography*, 2011, **44**, 1281-1284.
20. G. Sheldrick, *Acta Crystallographica Section C*, 2015, **71**, 3-8.
21. *International Tables for Crystallography, Vol. C (Ed.: A. J. Wilson), Kluwer Academic Publishers, Dordrecht, The Netherlands, Tables 6.1.1.4 (pp. 500–502), 4.2.6.8 (pp. 219–222), and 4.2.4.2 (pp. 193–199).* 1992.
22. C. F. Macrae, I. J. Bruno, J. A. Chisholm, P. R. Edgington, P. McCabe, E. Pidcock, L. Rodriguez-Monge, R. Taylor, J. van de Streek and P. A. Wood, *Journal of Applied Crystallography*, 2008, **41**, 466-470.
23. A. Spek, *Acta Crystallographica Section D*, 2009, **65**, 148-155.
